# The Swi-Snf chromatin remodeling complex mediates gene repression through metabolic control

**DOI:** 10.1093/nar/gkad711

**Published:** 2023-08-31

**Authors:** Michael C Church, Andrew Price, Hua Li, Jerry L Workman

**Affiliations:** Stowers Institute for Medical Research, Kansas City, MO 64110, USA; Stowers Institute for Medical Research, Kansas City, MO 64110, USA; Stowers Institute for Medical Research, Kansas City, MO 64110, USA; Stowers Institute for Medical Research, Kansas City, MO 64110, USA

## Abstract

In eukaryotes, ATP-dependent chromatin remodelers regulate gene expression in response to nutritional and metabolic stimuli. However, altered transcription of metabolic genes may have significant indirect consequences which are currently poorly understood. In this study, we use genetic and molecular approaches to uncover a role for the remodeler Swi-Snf as a critical regulator of metabolism. We find that *snfΔ* mutants display a cysteine-deficient phenotype, despite growth in nutrient-rich media. This correlates with widespread perturbations in sulfur metabolic gene transcription, including global redistribution of the sulfur-sensing transcription factor Met4. Our findings show how a chromatin remodeler can have a significant impact on a whole metabolic pathway by directly regulating an important gene subset and demonstrate an emerging role for chromatin remodeling complexes as decisive factors in metabolic control.

## Graphical Abstract

**Graphical Abstract:**
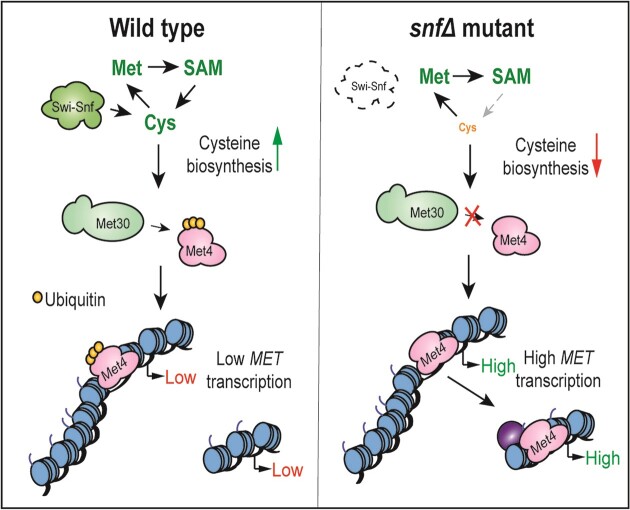

## INTRODUCTION

Metabolic perturbations are commonly found in cancer. The tendency for tumor cells to favor aerobic glycolysis over respiration (the ‘Warburg effect’) was an early clue that metabolic reprogramming occurs in transformed cells (1,2). Impaired respiration can be due in part to altered transcription of glycolytic enzymes under the control of the oncoprotein c-Myc (3). This biases glycolysis towards lactate production and thus can reduce aerobic respiration in cancer cells. However, altered respiration is not the only metabolic perturbation found in cancer as many tumor cells also show altered amino acid biosynthesis, in particular relating to methionine metabolism (4). Such cells are said to be ‘addicted to methionine’, as they require large amounts of the amino acid to synthesize S-Adenosyl Methionine (SAM/AdoMet) which is a cofactor required to carry out methylation reactions in the cell (5). Another metabolic signature of many tumor cells is that they require cysteine as an essential amino acid, in contrast to healthy cells which can synthesize cysteine from dietary methionine (6).

In addition to metabolic abnormalities, transcriptional patterns are altered in cancer cells and many transcriptional regulators are known to be oncogenes or tumor suppressors. One example is SWI/SNF, a conserved chromatin remodeling complex found to be mutated in > 20% of all cancers (7). Chromatin itself consists of repeating arrays of nucleosomes, which are a DNA-protein complex generally considered to be an impediment to gene activation (8). However, nucleosomes can be enzymatically modified or remodeled to regulate gene transcription. SWI/SNF displaces/evicts nucleosomes in an ATP-dependent manner and was first described in *Saccharomyces cerevisiae* (Swi-Snf in *S. cerevisiae*) where it was found to remodel chromatin and facilitate binding of transcription factors to nucleosomal DNA (9–14). By promoting a more open chromatin conformation upstream of transcription start sites (TSSs) and facilitating transcription factor binding, SWI/SNF can positively influence gene transcription. Yeast and human SWI/SNF are large complexes that contain multiple subunits, including the catalytic ATPase (Snf2/SMARCA2/SMARCA4) and other subunits which are essential for the structure/activity of the complex (15–19). Several human SWI/SNF subunits have been shown to be involved in cancer, with mutations in some being strongly correlated with specific malignancies, such as is the case with INI1/SMARCB1/SNF5 and rhabdoid tumors (17–18,20). In yeast, the complex is closely associated with activation of stress-response genes, particularly those involved in mating type switching and carbon source utilization (21,22).

In contrast to its association with gene activation, loss of Swi-Snf in *S. cerevisiae* leads to activation of several metabolic genes under repressing conditions, particularly those involved in synthesis of the amino acids methionine and cysteine (16,23). These genes (known as *MET* genes) are ordinarily repressed during growth in the presence of methionine and other sulfur metabolites but are activated by the transcription factor Met4 upon sulfur starvation (24–27). *MET* gene-containing pathways are involved in amino acid biosynthesis, redox homeostasis, cell cycle progression and cell signaling (28). As transcription of metabolism genes responds to cellular nutrition, the activation of *MET* genes in *snfΔ* mutants could hint at a metabolic imbalance in these cells caused by loss of Swi-Snf. Therefore, Swi-Snf may have a novel, direct role in maintaining levels of important metabolites via an unknown mechanism. Evidence for a conserved role for Swi-Snf in regulating genes involved in sulfur amino acid metabolism has been found in ovarian cancer cell lines, where the human SWI/SNF complex was shown to be essential for activation of a gene encoding a cysteine transporter (29).

Here, we identify Swi-Snf as a regulator of a key point in sulfur metabolism, the loss of which leads to impaired cysteine biosynthesis during growth in rich media. This defect is sensed by the transcription factor Met4, whose global recruitment becomes altered in response to loss of Swi-Snf, in a manner resembling that seen in starved cells. Met4 is activated and recruited to several sulfur metabolism genes, leading to their activation under repressive conditions. Together, this demonstrates that Swi-Snf has a more prominent role in metabolic regulation than previously appreciated, both through its direct regulation of genes required for cysteine biosynthesis and the indirect consequences of Swi-Snf inactivity on metabolic transcription.

## MATERIALS AND METHODS

### Reagents

Antibodies: anti-Myc (Millipore 05-724), β-Actin (Abcam ab8224), HA-peroxidase (Sigma Aldrich 12013819001), V5 (Thermo Fisher Scientific R960-25), GST (Santa Cruz sc-459), Rpb1 (BioLegend 664906). Metabolite assays were carried out using a Bridge-It® SAM fluorescence assay (Mediomics 1-1-1003B), or a Bridge-It® l-Methionine (L-Met) Fluorescence Assay (Mediomics 1-1-1005B).

### Biological resources

All strains and plasmids used are listed in Supplementary Tables S1 and S2, respectively. For analysis of amino acid starvation, cells were grown to mid-log phase (OD_600_ = 0.6–0.8) at 30°C in media containing yeast extract with peptone supplemented with 2% dextrose (YPD), subjected to centrifugation and cell pellets were washed twice in sterile ddH_2_O. Pellets were then resuspended in 1 ml sterile ddH_2_O and split between Complete Synthetic Medium (CSM) with/without methionine/cysteine (CSM-Met) or any amino acids (SD). CSM refers to BD Difco Yeast Nitrogen Base without amino acids (BD 291920) with CSM/ CSM-Met mix (Sunrise Science 1001-100)/(Sunrise Science 1019-010) and 2% glucose unless otherwise stated.

### Statistical analyses

Details of high-throughput data analysis can be found in Supplementary Materials and Methods. Details of statistical analysis of data can be found in figure legends. In all cases error bars represent standard deviation (SD). Significance was defined based on a Student's T-test in each case. When comparing different strains, unpaired parametric tests were used. When comparing treated/untreated conditions within the same sample, paired parametric tests were used. Graphpad Prism (9.3.1) was used for statistical calculations concerning qPCR and cell viability data.

### Western blot

Whole cell lysate was prepared as described previously (30) and applied to a NuPAGE Bis-Tris 3–8% gradient gel (Thermo Fisher Scientific EA0375BOX) and run at 150 V for 1 h. Proteins were transferred to a Immobilon®-P PVDF Membrane (Millipore IPVH00010) in 1× NuPAGE transfer buffer (Thermo Fisher Scientific NP0006) with 20% methanol for 90 min at 350 mA. Membranes were blocked for 30 min in 5% dried skimmed milk in TBST before being incubated with primary antibodies overnight at 4°C. For non-HRP-conjugated antibodies, membranes were washed in TBST and incubated with a secondary, HRP-conjugated antibody diluted 1:10 000 in 5% dried skimmed milk in TBST for 45 min at room temperature. Following incubation with HRP-conjugated antibodies, membranes were washed in TBST and TBS before being incubated for 5 min in Pierce™ ECL Plus Western Blotting Substrate (Thermo Fisher Scientific 32132 × 3) and development.

### Metabolite assays

SAM and methionine were measured, based on a previously-published protocol (31). Briefly, yeast were grown to mid-log phase (OD_600_ ∼0.6–0.8), and 20 OD units were collected and pelleted. Cells were washed twice in ddH_2_O and then resuspended in 100 μl 0.2% perchloric acid prior to incubation at room temperature for 1 hour. Samples were then centrifuged at 10 621 rcf for 5 min and supernatant was transferred to new tubes. 10 μl (SAM) or 5 μl (Met) sample (∼1–2 OD units) was used for each replicate. Fluorescence was measured using a Tecan Infinite 200 Pro plate reader.

### Anchor away

Proteins of interest were tagged using an FRB-GFP construct based on that used by Haruki *et al.* in the HHY221 background ((32), Supplementary Table S1). To deplete tagged proteins from nuclei, cells were grown to log phase (OD_600_ ∼0.5) at 30°C and incubated with rapamycin (TSZ Chem R1017) at a final concentration of 1 μg/ml for times indicated. To verify nuclear export of proteins, cells were analyzed by confocal microscopy using a Leica LSM-780 DS. To measure transcription, samples were taken before and after rapamycin treatment and processed for RT-qPCR or sequenced as described below.

### RNA preparation

Cells were grown to mid-log phase (OD_600_ ∼0.6–0.8) at 30°C and pelleted by centrifugation, washed once with DEPC-H_2_O, and either stored at −80°C or processed as follows:

For samples analyzed by RT-qPCR, RNA was prepared by hot phenol extraction as described in (33). 10 μg RNA was DNase-treated using RQ1 RNase-Free DNase (Promega). 1 μg of DNase-treated RNA was then used to generate cDNA using the Applied Biosystems High-Capacity RNA-to-cDNA Kit (ThermoFisher Scientific). This was analyzed using PerfeCTa SYBR Green Fastmix, Low ROX (QuantaBio 95074-05K) in an Applied Biosystems QuantStudio 5 thermocycler (Thermo Fisher Scientific). Two to six biological (as outlined in figure legends) and 3 technical replicates were analyzed for each experiment. Error bars for RT-qPCR data represent standard deviation of biological replicates. *P*-values were calculated using a paired (for comparing treatments within strains) or unpaired (when comparing different strains) Student's t-test using GraphPad Prism 9.0. All RT-qPCR primers used are listed in Supplementary Table S3.

For samples to be analyzed by sequencing, 3 OD units of cells were processed using the Qiagen RNeasy Mini Kit according to manufacturer's instructions. An on-column DNase I (Zymo Research E1010) digestion was carried out for 15 min at room temperature prior to elution and sequencing.

### Chromatin immunoprecipitation (ChIP)

ChIP was performed as described previously (33). Briefly, cells were grown to mid-log phase (OD_600_ ∼0.5–0.8), and crosslinked by incubating in 1% formaldehyde (Sigma Aldrich 252549-500ML) at room temperature for 15 min, before quenching in 125 mM glycine. Cell pellets were lysed using 400 μl acid-washed glass beads in lysis buffer (50 mM HEPES, 140 mM NaCl, 1 mM EDTA, 1% Triton X-100, 0.1% sodium deoxycholate). Lysate was sonicated (8–10 pulses at 20% power, 10s on, 30s on ice) using a Branson Sonicator to yield DNA fragments between 100 and 500 bp, before being clarified by centrifugation at 18000 rcf, 30 min at 4°C. Clarified lysate was divided into 10 or 40 OD_600_ unit aliquots, to be used for ChIP-qPCR or ChIP-seq, respectively. Clarified lysates were incubated with at 4°C after reserving a portion as an Input, which was treated with Pronase (Sigma 10165921001), and crosslinks reversed. Following antibody incubation, lysates were incubated with 30 μl magnetic Dynabeads (Thermo Fisher Scientific 10003D) for 2 h and washed before elution, Pronase treatment and reversal of crosslinks. DNA was purified using a QIAquick PCR Purification kit (Qiagen). IPs/Inputs were then either analyzed by qPCR or sequenced.

### High throughput sequencing

Sequencing libraries were prepared using High Throughput Library Prep Kit (KAPA Biosystems) or NEBNext Ultra II DNA Library Prep Kit for Illumina following the manufacturer's instructions. The library was sequenced on an Illumina HiSeq platform with paired-end reads of 75 bp for both ChIP-seq and RNA-seq. Whole-genome RNA-seq comparisons between mutants/wild type and Met4 ChIP cpm data, in addition to *MET*-regulon specific RNA-seq and Rpb1 ChIP cpm data in Met4-AA strains can be found in Supplementary File S2.

## RESULTS

### Loss of Swi-Snf results in *MET* gene activation in rich medium

**Figure 1.**
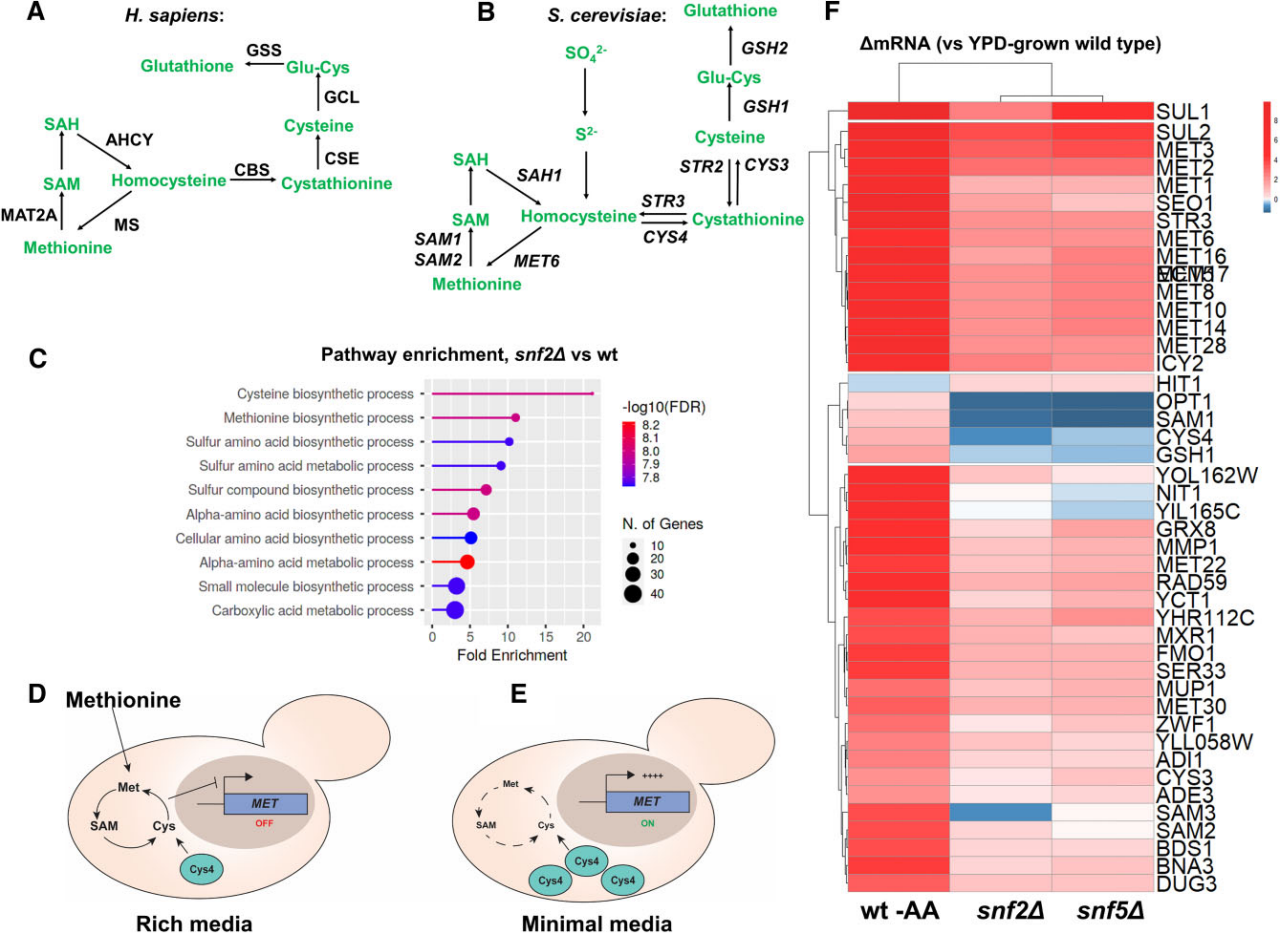
Loss of Swi-Snf results in *MET* gene activation in rich medium. (**A**) The human methionine biosynthetic pathway, adapted from (34). (**B**) The *S. cerevisiae* methionine biosynthetic pathway, adapted from (35). (**C**) GO term analysis of all DE-upregulated transcripts in YPD-grown *snf2Δ* mutant vs wild type (wt) cells, analyzed using ShinyGO (54). (**D**, **E**) Model for sulfur-mediated repression of *MET* gene transcription in yeast, showing increased production of sulfur metabolism enzymes (Cys4) during starvation. (**F**) Heatmap representing log_2_ fold-change vs wild type of *MET* regulon gene transcript levels in SD-grown wild type (wt SD) and YPD-grown *snf2Δ* and *snf5Δ* mutants from an RNA-seq experiment.

Swi-Snf is well known as an activator of gene transcription, hence the fact that loss of Swi-Snf led to activation of sulfur metabolism (*MET*) genes in *S. cerevisiae* was puzzling. Additionally, SWI/SNF is an important factor in human disease and its role as a metabolic regulator deserves further study. *S. cerevisiae* possesses most sulfur metabolic enzymes/pathways found in mammals, including the enzymes cystathionine beta synthase (CBS; Cys4 in yeast) and glutathione synthetase (GSS; Gsh2 in yeast), making it an attractive model to study these pathways (Figures 1A, B, Supplementary Figure S1C) (34,35).

To monitor the effect of Swi-Snf loss on *MET* gene transcription, we performed RNA sequencing (RNA-seq) in rich medium (YPD)-grown *snf2Δ* and *snf5Δ* mutants, comparing total transcript levels to wild type cells (Supplementary Figure S1A) (16,23). An unbiased pathway enrichment analysis revealed that *MET* gene-containing pathways are some of the most highly enriched among upregulated transcripts in *snfΔ* mutants vs wild type cells (Figure 1C and Supplementary figure S1B) (16,23). Several genes involved in sulfur metabolism share a common regulatory network known as the *MET* regulon, and this overlaps with genes in the pathways shown in Figures 1C and S1B, though the genes *SAH1*, *STR2* and *GSH2* are not included in the *MET* regulon (26). *MET* genes are generally activated in response to sulfur metabolite starvation, and repressed during growth in rich media (Figure 1D, E) (24). Remarkably, when the *MET* regulon gene transcription was specifically analyzed, it was found that many *MET* genes were activated in YPD-grown *snfΔ* mutants, compared to wild type (Figure 1F). These genes were selected for analysis as they had been previously found to share a common activator (26). Interestingly, the most highly-induced *MET* genes in the *snfΔ* mutants also tended to be highly induced during amino acid starvation in wild type cells (wt SD) suggesting that these mutants were exhibiting a starvation phenotype (Figure 1F, compare wt SD to *snf2Δ* & *snf5Δ*).

Because *snfΔ* mutants had elevated *MET* transcription, we hypothesized that these cells could have reduced levels of some sulfur metabolites compared to wild type cells. One possibility was that loss of Swi-Snf resulted in a methionine uptake defect leading to low intracellular methionine, which would explain the transcriptional effect. To test this, methionine was measured in wild type vs *snf2Δ* and *snf5Δ* cells (Supplementary Figure S1D). *snfΔ* mutants exhibited a noticeably elevated methionine concentration vs wt, demonstrating that these mutants do not have a defect in methionine biosynthesis or uptake in YPD. To confirm that our method could detect reduced levels of methionine in our strains, we also starved cells of amino acids (SD medium) and measured methionine (Supplementary Figure S1D). We next measured S-adenosyl methionine (SAM/AdoMet), whose availability can be sensed by the cell as a means of discerning sulfur status (24). Alterations in SAM levels can affect histone methylation and *MET* gene expression (36,37). To investigate whether *snfΔ* mutants had a SAM deficiency, we measured SAM levels in wild type, *snf2Δ*, and *snf5Δ* mutant cells (Supplementary Figure S1E). It was found that *snfΔ* mutants had wild-type SAM levels, and therefore SAM deficiency cannot explain the observed *MET* gene induction in *snfΔ* mutants.

These data show that surprisingly, loss of Swi-Snf in YPD-grown cells correlates with a transcriptional phenotype resembling starvation, suggesting altered sulfur metabolism in these cells. However, this cannot be explained by a failure of mutants to synthesize SAM, or to use methionine available in growth media.

### Exogenous cysteine cures the transcriptional defect in *snfΔ* mutants

**Figure 2.**
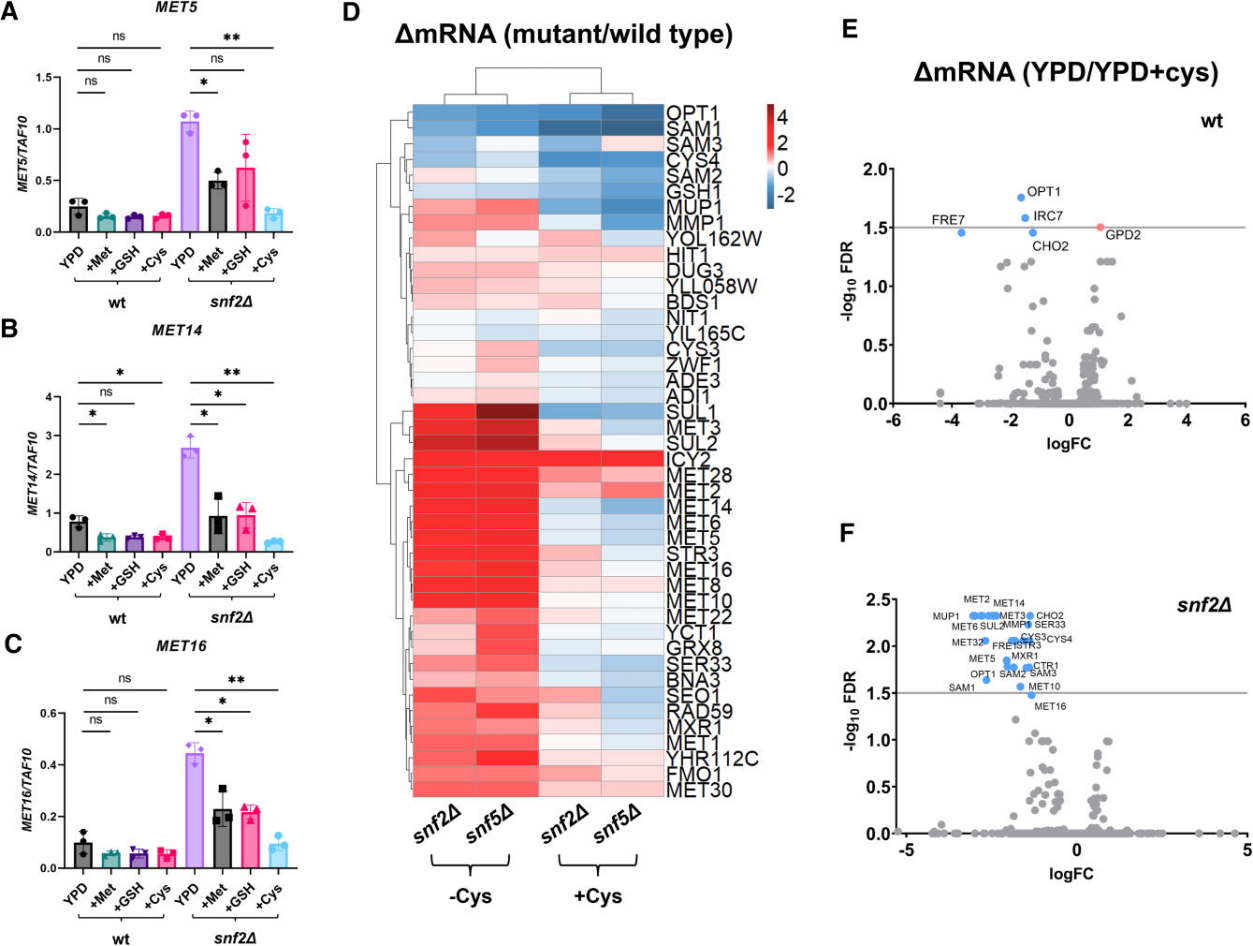
Exogenous cysteine cures the transcriptional defect in *snfΔ* mutants. RT-qPCR data showing (**A**) *MET5*, (**B**) *MET14* and (**C**) *MET16* transcription in wild type (wt) and *snf2Δ* strains after incubation in YPD, YPD containing 3 mM methionine (YPD + Met), 3 mM GSH (YPD + GSH) or 3 mM cysteine (YPD + Cys). Transcript levels normalized to *TAF10*. Error bars represent standard deviation from three independent experiments. *P* values indicated by asterisks, with a *P* value ≤0.05 being considered statistically significant (1 asterisk) and *P* ≤ 0.01 being represented by two asterisks. (**D**) Heatmap representing log_2_ fold-change versus wild type transcript levels in YPD-grown *snf2Δ* and *snf5Δ* mutants without (−Cys) or with (+Cys) the addition of 3 mM cysteine from an RNA-seq experiment. The -Cys condition is the same RNA seq data shown in Figure 1F. (**E**) Volcano plot using RNA-seq data, analyzing total transcription in wild type cells, comparing –Cys and + Cys conditions within a single strain. (**F**) Volcano plot using RNA-seq data, analyzing total transcription in *snf2Δ* cells, comparing −Cys and +Cys conditions within a single strain. Both (E) and (F), use a 1.5-fold –log_10_FDR cutoff for differential expression.

The transcription data for *snfΔ* mutants grown in rich medium were similar to what would be expected in cells starved of sulfur. This was a puzzling result but hinted that Swi-Snf was required for synthesis of an important sulfur-containing metabolite. We hypothesized that if we could provide the correct metabolite exogenously, it may compensate for the deficiency and rescue *MET* transcription in YPD-grown *snfΔ* mutants. Growth in the absence or presence of 3 mM methionine, GSH or cysteine revealed that exogenous methionine and GSH led to a significant reduction in transcription of several *MET* genes in *snfΔ* mutants compared to wild type (Figures 2A–C and S2B–D). However, under these conditions *MET* transcription was still substantially higher than in wild type cells. Strikingly, incubation in the presence of exogenous cysteine rescued *MET* transcription to a greater degree than either methionine or GSH, resulting in wild type-level transcription at *MET5, MET14* and *MET16* (Figures 2A–C and S2B–D).

Due to its instability, it is difficult to accurately measure intracellular cysteine, but we hypothesized that if *snfΔ* mutants had a cysteine deficiency, most of the *MET* regulon should be affected. We therefore grew cells with (+Cys) or without (−Cys, data from Figure 1F) exogenous cysteine and measured transcription by RNA-seq. We found that *MET* regulon transcript levels in *snfΔ* mutants were almost entirely restored to wild type levels after 30 min’ growth in the presence of cysteine (Figure 2D).

We next wished to determine whether cysteine supplementation was affecting *MET* transcription specifically, or altering global transcription generally. Surprisingly, there were relatively few genes affected in wild type cells in +Cys samples compared to −Cys (Figure 2E). However, specific repression of many *MET* genes in a *snf2Δ* mutant was apparent in +Cys conditions compared to −Cys (Figure 2F). GO term analysis also showed that all pathways significantly repressed in *snfΔ* mutants during growth in 3 mM cysteine are involved in sulfur metabolism (Supplementary Figure S2A). This showed that cysteine could specifically resolve the transcriptional defect in *MET* genes caused by loss of Swi-Snf and was not resulting in widespread transcription effects in wild type cells. This also showed that unexpectedly, *snfΔ* mutants had a cysteine starvation phenotype during growth in rich media and that this was likely related to the elevated *MET* gene transcription in this background. However, we were still unable to determine why these cells had a phenotype similar to cysteine-starved cells.

### Defective cysteine biosynthesis is the cause of elevated *MET* transcription in *snfΔ* mutants

**Figure 3.**
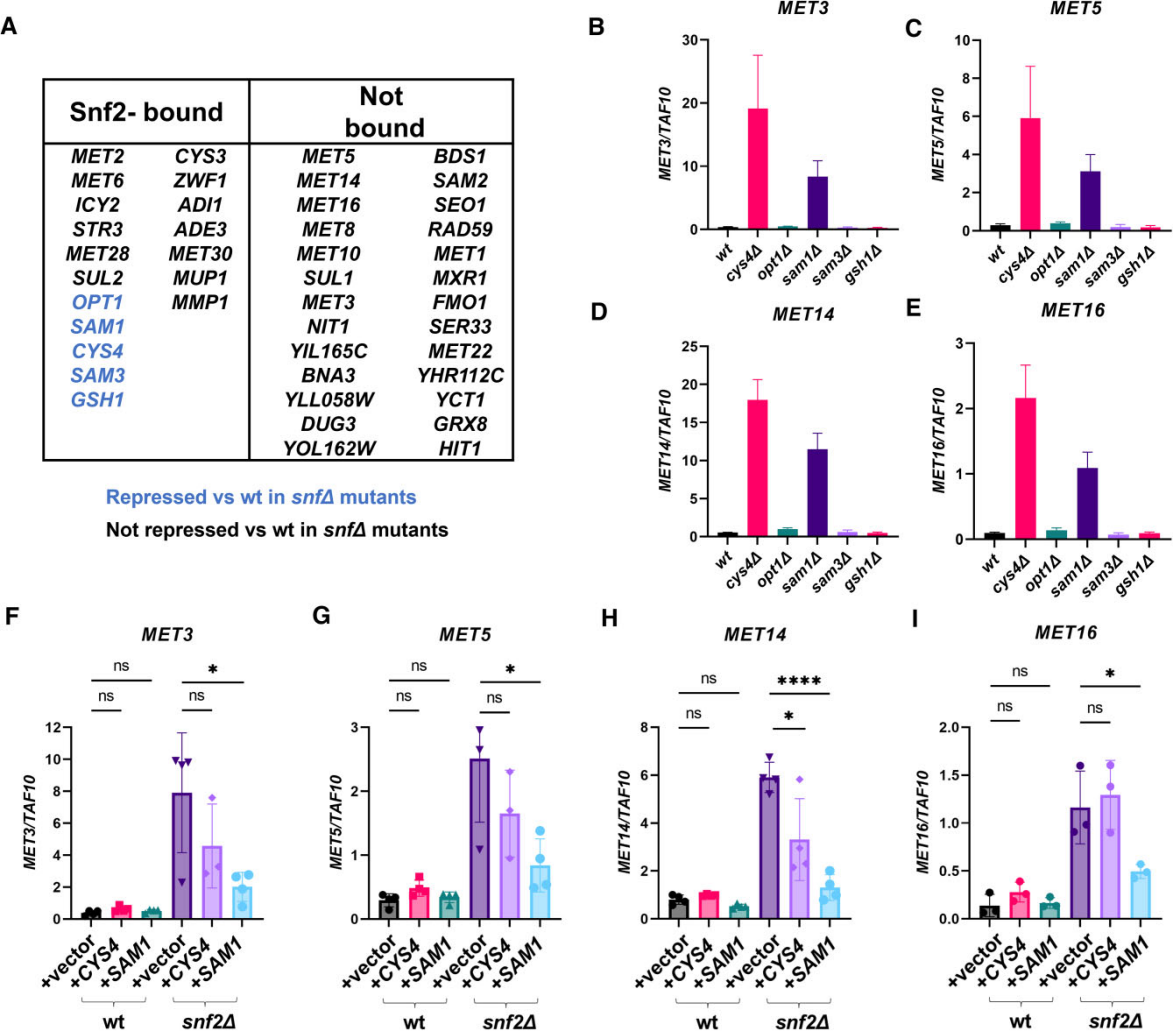
Defective cysteine biosynthesis is the cause of elevated *MET* transcription in *snfΔ* mutants. (**A**) ChIP data from Dutta *et al.* showing Snf2 occupancy at *MET* regulon promoters (18 out of 1372 total promoters identified (38)). Genes in blue are significantly repressed in *snf2Δ* mutants grown in YPD compared to wild type cells. RT-qPCR data showing transcription of (**B**) *MET3*, (**C**) *MET5*, (**D**) *MET14* and (**E**) *MET16* in wild type (wt) cells and strains lacking *MET* genes whose transcription is repressed vs wild type in YPD-grown *snfΔ* mutants, based on RNA-seq data (**F–I**) RT-qPCR data showing *MET* transcription in CSM-Ura grown wild type and *snf2Δ* mutants containing an empty vector (pRS416), or overexpressing *SAM1* or *CYS4*. Transcript levels normalized to *TAF10*. Error bars represent standard deviation of 2–6 (A–D) or 4 (F–I) independent experiments. *P* values indicated by asterisks, with a *P* value ≤0.05 being considered statistically significant (one asterisk) and *P* ≤0.0001 being represented by four asterisks.

We had established that *snfΔ* mutants exhibited a cysteine starvation phenotype, and that this correlated with activation of the *MET* regulon under repressing conditions, but we had not identified the cause of this apparent starvation. We reasoned that the most likely cause was repression of one or more genes required for cysteine biosynthesis in *snfΔ* mutants. Biosynthesis of cysteine in *S. cerevisiae* first involves synthesis of SAM from methionine by Sam1/Sam2. SAM is used as a cofactor in methylation reactions, where its methyl group is removed yielding S-adenosyl homocysteine (SAH), which is used to synthesize homocysteine. Homocysteine is then used to synthesize cysteine via cystathionine by Cys4 and Cys3, respectively (Figure 1B) (24,35). If Swi-Snf directly regulated genes involved in cysteine biosynthesis, then it should be detectable at such gene promoters under nutrient-rich conditions. We therefore used previously published Snf2 ChIP data from YPD-grown cells, and found that Snf2 was detected at ∼40% of *MET* gene promoters, and this occupancy correlates with higher transcription in YPD-grown cells vs promoters not bound by Swi-Snf (Figures 3A and S3B) (38). Moreover, several Snf2-bound genes were also repressed in *snfΔ* mutants, based on our RNA-seq data (Figure 3A, blue genes), indicating that Swi-Snf is a direct activator of these genes. A number of the Swi-Snf-activated genes could be required for cysteine biosynthesis. To determine whether dysregulation of any of these genes could explain the *snfΔ* mutant phenotype, we measured *MET* gene transcription by RT-qPCR in mutants (Figure 3B–E). We found that while most deletions had no effect on *MET* gene transcription, loss of *CYS4* or *SAM1* led to noticeable de-repression of all genes studied*. cys4Δ* and *sam1Δ* mutants have cysteine synthesis defects, impairing the ability of cells to synthesize cysteine from methionine and therefore these genes were good candidates for the cause of cysteine deficiency in *snfΔ* mutants (36,39,40).

As *S. cerevisiae* contains two SAM synthetases (*SAM1* and *SAM2*), we wanted to test whether loss of *SAM2* would lead to a similar phenotype to loss of *SAM1*, and so we monitored *MET* transcription in both *sam1Δ* and *sam2Δ* mutants. Strikingly, while a *sam1Δ* mutant showed de-repression of *MET* transcription (Supplementary Figure S3G–J), loss of *SAM2* did not lead to a similar phenomenon. Loss of either gene also differentially affected transcription of the other, in a manner similar to what was described previously (Supplementary Figure S3L, M) (41). Interestingly, loss of either *SAM1* or *SAM2* led to repression of *CYS4*, to different extents (Supplementary Figure S3K).

Our data confirmed that *snfΔ* mutants shared a *MET* de-repression phenotype with *cys4Δ* and *sam1Δ* mutants, but to determine the direct contribution of *CYS4* or *SAM1* loss in a *snfΔ* mutant, we constructed vectors containing a V5- or HA-tagged Cys4 or Sam1, respectively, both under the control of a *TEF1* promoter for a high level of constitutive expression. These constructs were transformed into wild type and *snf2Δ* mutant cells, and their expression was confirmed before *MET* transcription was measured (Figures 3F–I, S3A, C, D). Overexpression of either gene had little effect on wild type cells, but overexpression of *SAM1* in a *snf2Δ* mutant significantly reduced the level of *MET3*, *MET5*, *MET14* and *MET16* transcription, (Figure 3F–I, compare *snf2Δ* + vector to *snf2Δ* + *SAM1*). Overexpression of *CYS4* was found to significantly reduce *MET14* transcription in a *snf2Δ* mutant, and *MET3* and *MET5* transcript levels were reproducibly reduced compared to a vector control (Figure 3F–H, compare *snf2Δ* + vector to *snf2Δ* + *CYS4*). In the case of *MET16*, despite a significant rescue in *snf2Δ* mutants overexpressing *SAM1*, overexpression of *CYS4* did not affect transcript levels compared to the vector (Figure 3I). This suggests that in *snfΔ* mutants, loss of *SAM1* transcription may be the dominant factor in impairing cysteine biosynthesis, with *CYS4* also contributing.

Importantly, these data support a role for Swi-Snf as a direct regulator of genes that are required for cysteine biosynthesis. We have shown that restoring transcription of these genes is sufficient to reduce *MET* transcription in *snfΔ* mutants. Therefore, we have identified the cause of the cysteine starvation phenotype characteristic of *snfΔ* mutants, which ultimately leads to widespread perturbations in metabolic gene transcription.

### Loss of Swi-Snf leads to activation and altered recruitment of Met4 in rich media

**Figure 4.**
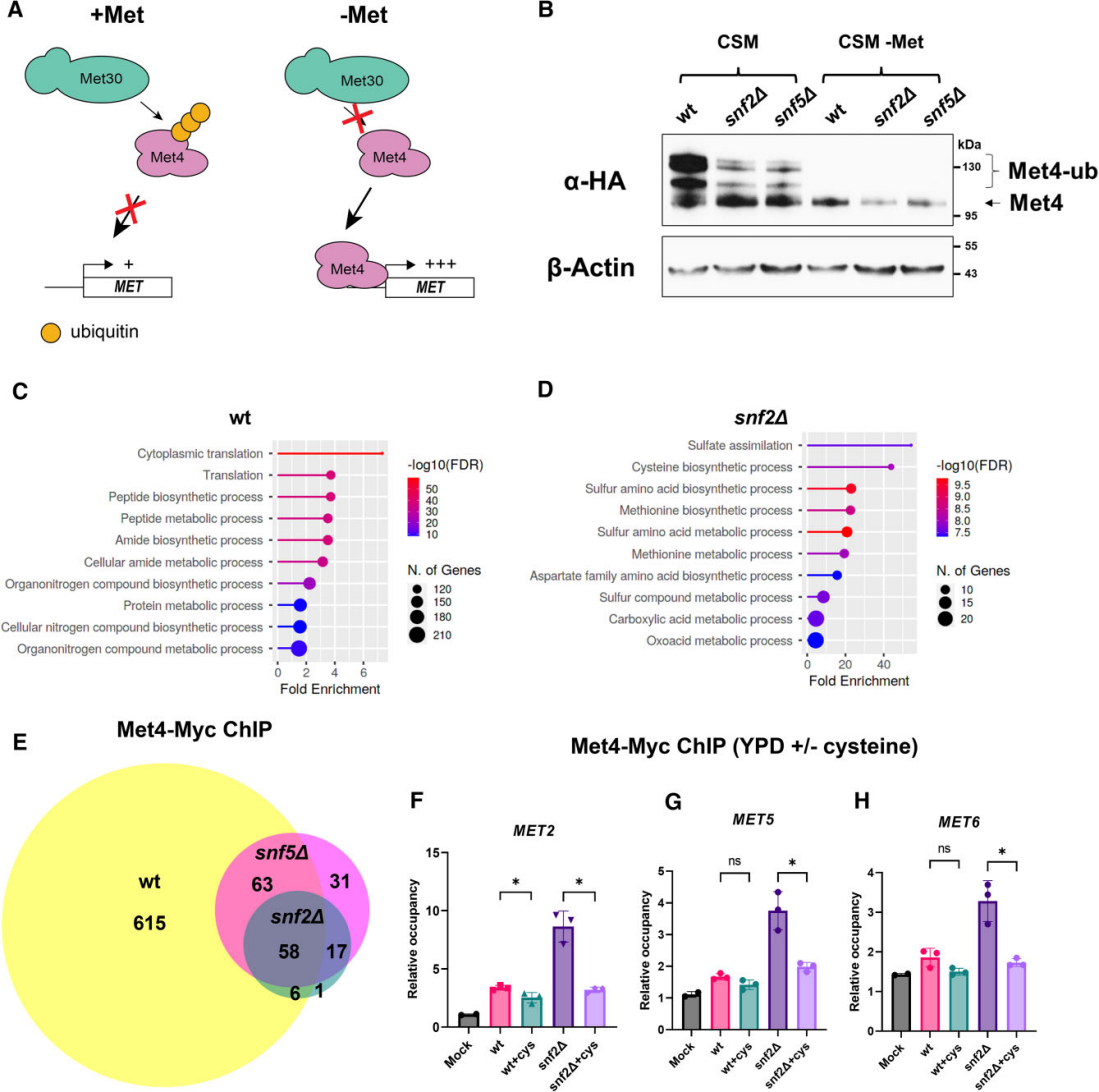
Loss of Swi-Snf leads to activation and global redistribution of Met4 in rich media. (**A**) Model depicting sulfur-mediated control of Met4 activity. (**B**) Western blot of HA-tagged Met4 in wild type (wt), *snf2Δ* and *snf5Δ* mutant cells grown in media with (CSM) and without (CSM-Met) methionine. Active (Met4) and inactive (Met4-ub) Met4 species are indicated. Representative blot shown from three independent experiments. (**C**) GO term analysis (ShinyGO) for genes occupied by Met4-13Myc in YPD-grown wild type cells, as determined by IDR analysis. (**D**) GO term analysis for genes occupied by Met4-13Myc in YPD-grown *snf2Δ* mutant cells, as determined by IDR analysis. (**E**) Venn diagram of all Met4-13Myc peaks in wild type, *snf2Δ* and *snf5Δ* mutants (55,56). ChIP data shown represent two independent experiments. (F–H) Met4-Myc ChIP carried out in wild type and *snf2Δ* mutant cells grown in YPD with or without exogenous cysteine addition at the promoters of (**F**) *MET2*, (**G**) *MET5* and (**H**) *MET6*. Error bars represent standard deviation of three independent experiments. *P* values indicated by asterisks, with a *P* value ≤0.05 being considered statistically significant (one asterisk).

Met4 is the transcription factor responsible for activation of the *MET* regulon and hence many genes involved in sulfur metabolism in *S. cerevisiae*, with roles in heavy metal/oxidative stress protection and cell cycle progression (42,43). In the presence of sulfur metabolites, Met4 is normally ubiquitinated (Met4-ub) by the SCF^Met30^ ligase, potentially inhibiting the Met4 transactivation domains, whereas sulfur starvation results in deubiquitination of Met4 (Figure 4A, (25,44–46)). This ‘active’ species of Met4 can then be recruited to its target promotors via interaction with DNA-binding partners where it promotes *MET* gene transcription (26,47). We hypothesized that if loss of Swi-Snf resulted in cysteine deficiency in rich media, then this could result in altered Met4 ubiquitination. To test this, we grew wild type and *snfΔ* mutant cells in YPD and analyzed 3XHA-tagged Met4 (Met4-HA) from whole cell lysate by western blot in each background. In wild type cells grown in YPD, the majority of Met4 protein is of the higher molecular weight species, as would be expected for Met4-ub (Figure S4A lane 1) (44). However, loss of *SNF2* or *SNF5* resulted in a dramatic increase in the level of lower molecular weight Met4, which corresponds to the active species present in starved cells (Figure 4B lanes 2&3 versus lanes 4–6). To confirm that Met4 was ubiquitinated in wild type cells, ubiquitin was GST-tagged in a Met4-HA background and a coimmunoprecipitation was performed (Figure S4B).

We next wanted to determine whether this alteration in Met4 modification correlated with alteration of its genomic recruitment. We therefore performed ChIP-seq on 13XMyc-tagged Met4 (Met4-Myc) in wild type & *snfΔ* mutant cells. Comparison of pathway enrichment analysis data from wild type and *snfΔ* mutants grown in YPD showed that while Met4-Myc peaks are overrepresented at genes involved in translation & growth in wild type cells, these terms were not found in the *snf2Δ* mutant analysis, where instead pathways involved in sulfur/methionine/cysteine metabolism were enriched (Figure 4C, D). Interestingly, loss of Swi-Snf and activation of Met4 resulted in far fewer peaks in both *snfΔ* mutants compared to wild type cells (Figure 4E). This is in agreement with previous work that showed that during growth in the presence of methionine, Met4 occupied many loci despite remaining in its ubiquitinated state (26,48). However, increased Met4 binding was observed at several *MET* genes (including *MET2, MET5* & *MET6*) in *snfΔ* mutants compared to wild type cells (Figure 4F–H). In agreement with data shown above, incubation in the presence of exogenous cysteine both reduced the amount of deubiquitinated Met4 and eliminated the increased *MET* promoter binding observed in a *snf2Δ* background (Supplementary Figures S4E and 4F–H).

Although these results supported a role for Swi-Snf in maintaining sulfur homeostasis, it was possible that *snfΔ* mutants were simply defective for activation of genes encoding the SCF^Met30^ ligase responsible for inactivating Met4 during growth in rich media. To test this, transcription of the components of SCF^Met30^ was analyzed (Supplementary Figure S4G). It was found that loss of Swi-Snf did not reduce SCF^Met30^ gene transcription, and in the case of two genes (*MET30* and *CDC34*), a *snf2Δ* mutant displayed significantly higher transcript levels compared to wild type. The fact that *snfΔ* mutants show reduced Met4 ubiquitination despite higher *MET30* transcription may be due to post-translational regulation of Met30 activity (49). *MET4* transcript levels were slightly elevated in the *snf2Δ* mutant compared to wild type, though less than *MET30* (Supplementary Figure S4G). Transcript levels of the gene encoding the Met4-interacting transcription factor Cbf1 were also unchanged in mutant cells (Supplementary Figure S4F).

These data show that upon loss of Swi-Snf, Met4 is activated in a manner consistent with sulfur starvation. This also results in global redistribution of Met4 in *snfΔ* mutants, more specifically occupying promoters of genes involved in sulfur metabolism. Furthermore, *snfΔ* mutants can sense sulfur appropriately, and in these strains Met4 appears to be responding to the cysteine biosynthesis defect described above, resulting in its altered activity.

### Met4 occupancy changes in *snfΔ* mutants correlate with *MET* gene activation and a starvation response

**Figure 5.**
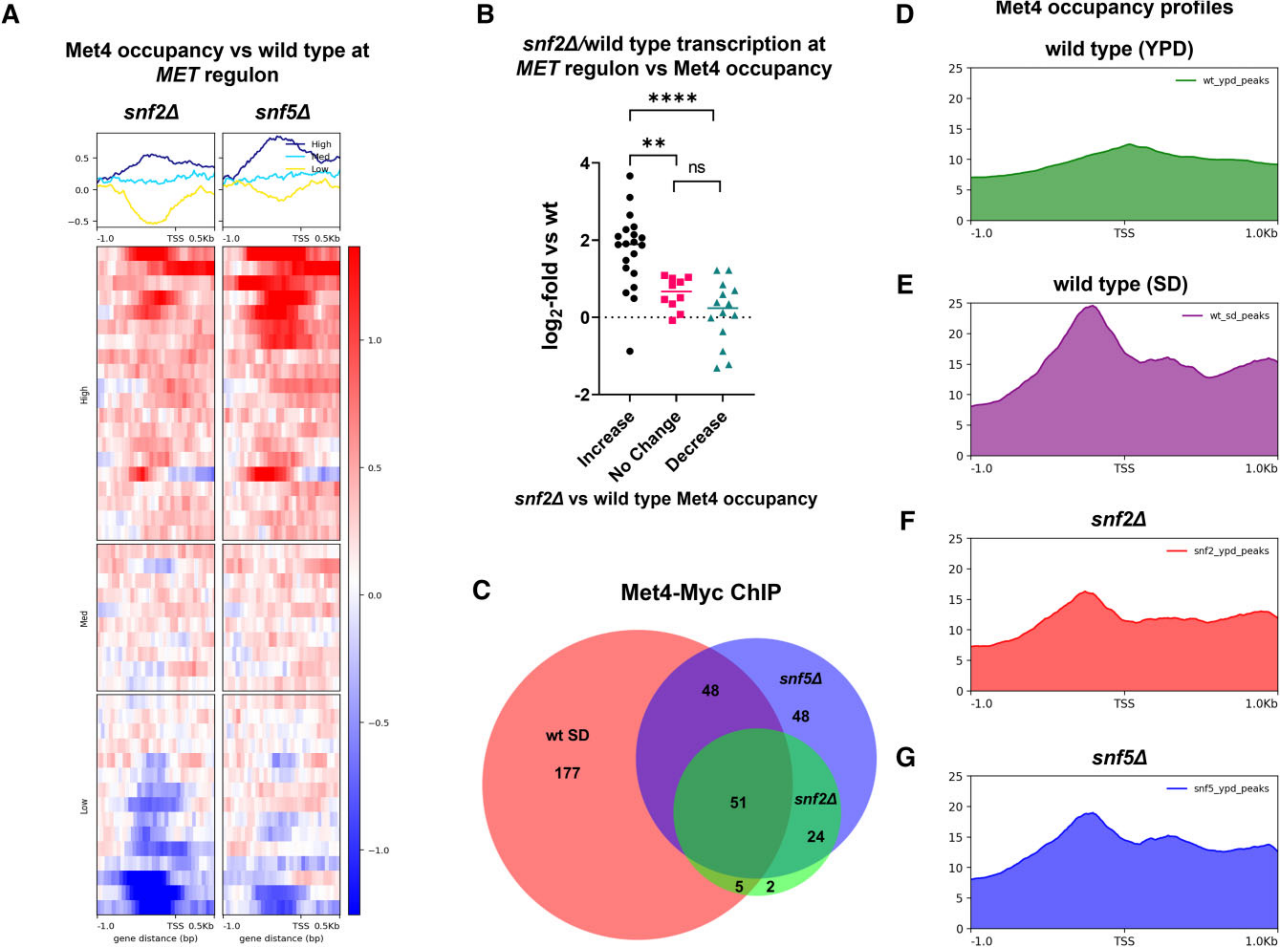
Met4 occupancy changes in *snfΔ* mutants correlate with *MET* gene activation and a starvation response. (**A**) Heatmap/profiles of Met4-Myc binding upstream of *MET* regulon genes in YPD-grown *snfΔ* mutants compared to wild type, separated into groups based on increased (High) unchanged (Mid) or decreased (Low) binding compared to wild type cells. (**B**) RNA-seq data comparing *MET* gene transcription in a *snf2Δ* mutant versus wild type cells, grouped by whether Met4-Myc occupancy increased, decreased or remained unchanged at the respective gene promoters based on ChIP-seq data. (**C**) Venn diagram of Met4-Myc peaks as determined by IDR in SD-grown wild type (wt SD) and YPD-grown *snf2Δ* and *snf5Δ* mutants (55) (D–G) profile of Met4-Myc occupancy ±1 kb relative to TSS in (**D**) YPD-grown wild type, (**E**) SD-grown wild type, (**F**) a YPD-grown *snf2Δ* mutant and (**G**) a YPD-grown *snf5Δ* mutant. In each case profiles were generated based on genes with Met4-Myc peaks as determined by IDR. *P* values indicated by asterisks, with a *P* value ≤0.01 being considered statistically significant (two asterisks) and *P* ≤ 0.0001 being represented by four asterisks.

Our data indicated that upon loss of Swi-Snf, Met4 was modified in a manner consistent with sulfur starvation (Figure 4B) and that its recruitment patterns were altered compared to wild type cells (Figure 4C–H). We next chose to specifically investigate Met4 occupancy at *MET* gene promoters, and found that in *snfΔ* mutants, Met4 levels increased at many promoters compared to wild type cells (Figure 5A, Supplementary File S1). However, there were several genes where Met4 levels remained constant or were reduced compared to wild type cells (Figure 5A, ‘Mid’ and ‘Low’). Throughout the *MET* regulon, changes in Met4 occupancy correlated to transcriptional changes (Figures 5B, S5A and S5D), however this correlation was much stronger at genes where Met4 levels increased in *snfΔ* mutants compared to wild type (Figure 5B).

As transcription patterns in *snfΔ* mutants resembled a wild type strain starved of amino acids (Figure 1F), we next wanted to compare Met4 recruitment in *snfΔ* mutants to starved cells, and so wild type cells were grown under these conditions (SD medium) for 30 min and Met4-13Myc occupancy was monitored by ChIP-seq (Figure 5C). It was found that starvation significantly reduced the number of Met4 peaks identified compared to YPD-grown cells (compare Figures 4E to 5C). GO-term analysis also showed that in starved wild type cells, Met4 peaks were overrepresented at genes involved in sulfur metabolism in contrast to what was seen in YPD-grown cells (compare Figures 4C and S5B). This more narrowly-focused binding to promoters of genes involved in metabolism is similar to what was observed in *snfΔ* mutants, and Met4 peaks in mutants overlapped a higher percentage of starved wild type peaks compared to YPD-grown cells (compare Figures 4E to 5C). The Met4 occupancy patterns at genes in the *MET* regulon observed in YPD-grown cells were altered similarly in starved wild type cells and *snfΔ* mutants grown in YPD, though the former showed stronger Met4 recruitment and did not show Met4 loss at some promoters as seen in *snfΔ* mutant cells (Supplementary Figure S5C).

As the many Met4-Myc peaks in YPD-grown wild type cells were unexpected because these cells had largely ubiquitinated Met4, we next wished to know where these peaks were actually located relative to the TSS. To test this, we monitored the occupancy profile of Met4-Myc bound to the TSS ±1 kb of all genes with verified peaks. Surprisingly, we found that YPD-grown wild type cells showed relatively low levels of enrichment at gene promoters on average, whereas SD-grown wild type and *snfΔ* mutant cells displayed defined peaks upstream of the TSS (Figure 5D–F). By measuring the ratio of Met4 occupancy upstream of the TSS to that found in the ORF, we determined that in YPD-grown wild type cells Met4 was more likely to localize to the gene body compared to starved or *snfΔ* mutant cells (Supplementary Figure S5E). This showed that not only were Met4-Myc peaks more prominently detected at genes involved in sulfur metabolism, but Met4 was more likely to be recruited to promoters in starved or *snfΔ* mutant cells compared to YPD-grown wild type.

These data suggest that in *snfΔ* mutants, Met4 occupancy increases at promoters of *MET* regulon genes compared to wild type cells and this recruitment is moderately correlated with transcriptional activation. Additionally, *snfΔ* mutants show similar patterns of Met4 recruitment to SD-grown wild type cells, both within the *MET* regulon and globally whereas in wild type cells grown in YPD, Met4 binding is not as strictly limited to gene promoter regions.

### Met4 is responsible for increased expression of *MET* transcription in the absence of Swi-Snf

We observed altered Met4 modification and occupancy at several *MET* gene promoters in *snfΔ* mutants (Figures 4 and 5). However, while Met4 was found to be recruited to *MET* genes in YPD-grown *snfΔ* mutants, this did not necessarily mean that this recruitment was the cause of elevated *MET* transcription. This is an important distinction, as it has been shown that Met4 may occupy some *MET* genes even in rich media (26,48) (Supplementary Figure S4D). To test whether Met4 was responsible for *MET* gene activation in *snfΔ* mutants, we generated a strain that could conditionally deplete nuclear Met4 via the anchor away method (32). This results in nuclear depletion of an FRB-tagged target protein upon incubation with rapamycin. Conditional Met4 loss is preferable to a steady-state *met4Δ* mutant, as stable *MET4* loss is known to frequently result in suppressor mutations that may interfere with gene regulation (39).

**Figure 6.**
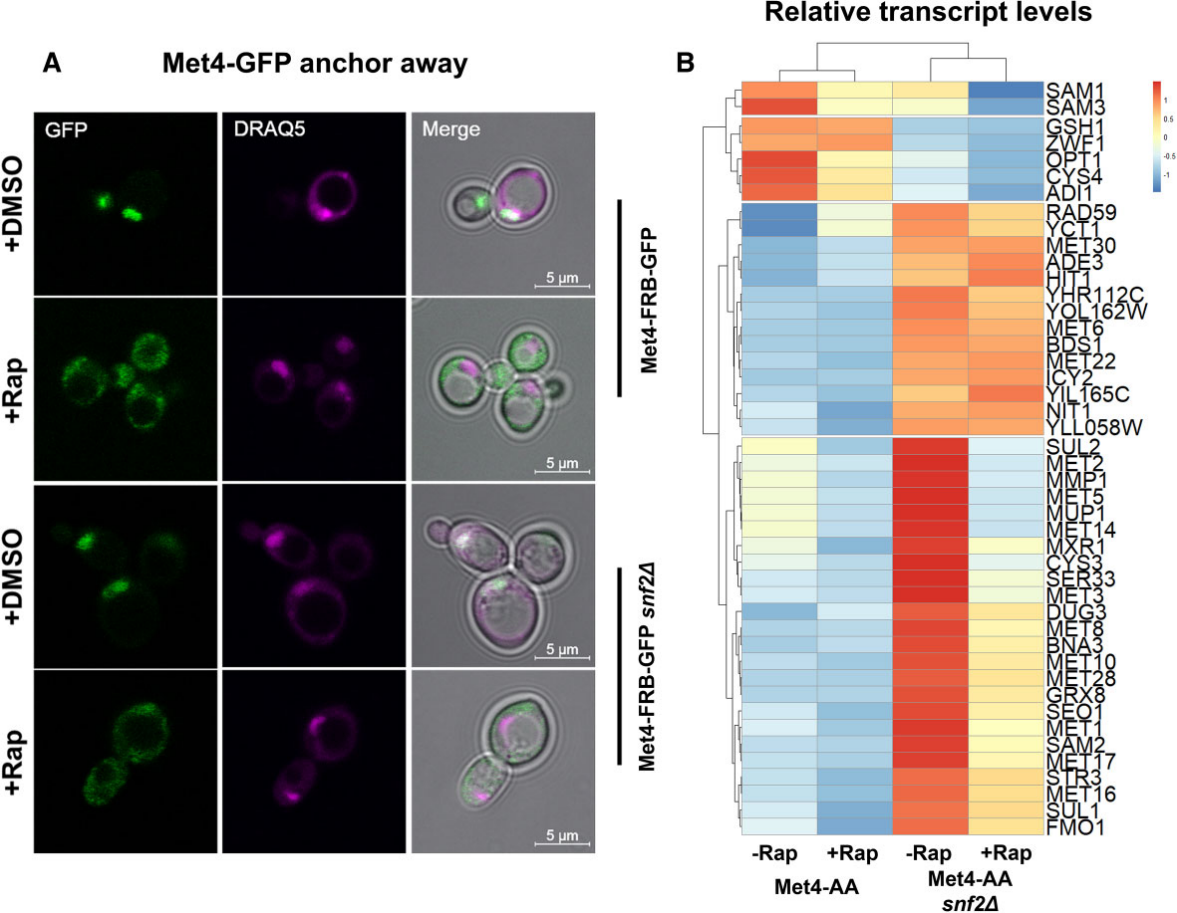
Active Met4 is responsible for increased expression of *MET* transcription in the absence of Swi-Snf. (**A**) Comparison of Met4-FRB-GFP localization between wild type and *snf2Δ* strains following incubation for 1 h in 1 μg/ml Rapamycin (+Rap) or an equivalent volume of DMSO (+DMSO). DRAQ5 staining of double-stranded DNA was used to visualize nuclei. (**B**) RNA-seq data showing *MET* transcription in wild type (Met4-AA) or *snf2Δ* (Met4-AA *snf2Δ*) strains.

To verify that Met4 was depleted from cell nuclei upon rapamycin treatment, a Met4-FRB-GFP (Met4-AA) strain was used and monitored by confocal microscopy in both *snf2Δ* mutant and wild type backgrounds following 60 min’ incubation in YPD with either rapamycin or DMSO (Figure 6A). While Met4-FRB-GFP is fully nuclear in the presence of DMSO (DMSO+), in rapamycin-treated samples (Rap+), Met4-FRB-GFP is lost from the nucleus both in wild type and *snf2Δ* mutant backgrounds (Figure 6A). A Met4-GFP strain lacking the FRB epitope was also monitored, and in these strains rapamycin addition did not affect Met4-GFP localization (Supplementary Figure S6A). Addition of cysteine to cell cultures using the anchor away strain background also affected *MET* gene transcription in a manner similar to other strains used (Supplementary Figure S6C, D).

Having established that Met4 could be depleted from nuclei, we wished to know if loss of Met4 in a *snf2Δ* mutant could affect *MET* gene de-repression in this strain. If *MET* genes were activated in a *snf2Δ* mutant due to increased Met4 activity in this background compared to wild type, we would expect that *MET* genes with elevated transcription in the mutant should show reduced transcription upon Met4 loss. We therefore measured transcription of the *MET* regulon by RNA-seq before and after 90 min’ incubation in the presence of rapamycin in both Met4-AA wild type and *snf2Δ* mutant backgrounds (Figure 6B). These data showed that loss of Met4 in wild type cells grown in rich media had little impact on transcription of the *MET* genes de-repressed in *snfΔ* mutants. In contrast to the wild type Met4-AA cells, loss of Met4 in the absence of *SNF2* (Met4-AA *snf2Δ*) decreased the de-repression of many *MET* genes, particularly those most highly activated in *snf2Δ* mutants vs wild type cells (Figure 6B). This striking result shows that Met4 activity is critical for the elevated transcription of many *MET* genes in *snfΔ* mutants.

To confirm that the observed effects were due to direct influence of Met4 on transcription rather than a post-transcriptional effect, occupancy of an RNA polymerase II (Pol II) subunit (Rpb1/Rpo21) was monitored by ChIP-seq at *MET* gene ORFs (Supplementary Figure S6B). It was found that Pol II occupancy increased at many *MET* genes in the *snfΔ* mutants, and that loss of Met4 reduced this occupancy in a manner similar to what was observed in the RNA seq data. Additionally, the subset of genes that were most sensitive to Met4 loss overlapped with those with increased Met4 recruitment in *snf2Δ* mutants based on Met4-Myc ChIP data shown in Figure 5A (Supplementary Figures S6B and S6E). Similarly, the group of genes with reduced Pol II occupancy in *snf2Δ* mutants overlapped with genes that showed Met4 loss in this strain compared to wild type cells (Supplementary Figures S6B and S6F).

These data demonstrate that upon loss of Swi-Snf, Met4 is recruited to genes in the *MET* regulon specifically in response to perceived cysteine deficiency, though the relationship between recruitment and activation is complex. However, once activated/recruited, Met4 is indispensable for de-repression of the majority of *MET* genes whose elevated expression is characteristic of *snfΔ* mutants grown under repressing conditions. This implies that as Met4 is crucial for the activation of much of the *MET* regulon in *snfΔ* mutants, controlling the level of cysteine biosynthesis appears to be the key role of Swi-Snf in exerting an effect on the *MET* regulon.

## DISCUSSION

We aimed to investigate a potential relationship between chromatin remodelers and sulfur metabolism based on findings which showed that loss of the remodeler Swi-Snf resulted in aberrant transcription of metabolic genes under repressing conditions (16,23). Both sulfur metabolism and the SWI/SNF complex are frequently altered in tumor cells, and we wished to explore the relationship between these factors. We used *S. cerevisiae* as a model to establish the basic interactions between Swi-Snf and sulfur metabolic pathways (the *MET* regulon) as a whole, rather than initially focusing on individual genes. Here, we have demonstrated that by directly regulating a crucial point in sulfur metabolism, the chromatin remodeler Swi-Snf can indirectly control a wider body of metabolic gene expression. This is a similar concept to what was described for cancer cells with respiration defects. c-Myc was found to activate *LDH-A*, which encodes an enzyme responsible for the conversion of pyruvate to lactate, and it was found that reducing *LDH-A* expression in a c-Myc-transformed cancer model could impair tumor cell growth (3). Overproduction of *LDH-A* in transformed cells was associated with a bias towards lactate production from pyruvate. To generate ATP from glucose, cells must ultimately commit their stores of pyruvate either to the TCA cycle, or to produce lactate. By overexpressing the *LDH-A* gene, cancer cells can bias this decision in favor of lactate production. Similarly, in a *snfΔ* mutant, reduced transcription of *SAM1* and *CYS4* biases sulfur metabolism towards methionine and away from cysteine synthesis.

Initially, we found many *MET* genes were activated in *snfΔ* mutants under repressing conditions (Figure 1F). Although initial experiments used the methionine auxotroph BY4741 strain, the most important findings were repeated in methionine prototrophs, supporting the original results (Figures 6, S6, S7). Previous studies had noted that several *MET* genes were de-repressed in *snfΔ* mutants, but the cause was unknown (16,23). We found that *snfΔ* mutants resemble starved cells, and incubation in the presence of exogenous cysteine cured the elevated *MET* transcription phenotype (Figure 2A–D). High levels of methionine and GSH also affected *MET* transcription (Figures 2A–C and S2B–D). Our data showed that additional loss of *CYS4* in a *snf2Δ* mutant abolished the ability of methionine to restore wild type levels of *MET* transcription, confirming that cysteine is the ultimate metabolite affecting *MET* transcription in these strains (Supplementary Figure S2E–G) (27). Mounting evidence suggests that cysteine is the primary signal sensed by Met4, which is modified in a manner similar to starved cells in *snfΔ* mutants (Figures 4B and S4A) (25,27,44).

The mechanism by which cysteine starvation occurs was found to be via repression of genes required for cysteine biosynthesis, namely *SAM1* and *CYS4*. Mutation of either gene led to elevated *MET* expression, similar to *snfΔ* mutants (Figure 3B–E) (36). While *SAM1* overexpression led to a more dramatic decrease in *MET* transcription in a *snf2Δ* mutant, *CYS4* was also shown to be a contributor to the phenotype (Figure 3F–I). These experiments were performed under nutrient-rich conditions, but analyzing *SAM1* and *CYS4* transcription in starved cells revealed a role for Swi-Snf in activating these genes under *MET* de-repressing conditions (Supplementary Figure S3E, F). The relative importance of *SAM1* compared to *SAM2* in this context is not fully known, though *SAM1* transcript levels appeared to be much higher under these conditions and increased expression of *SAM2* may not compensate in a *sam1Δ* mutant, despite the apparent redundancy of these enzymes (Supplementary Figure S3L, M).

Loss of Swi-Snf was also found to be correlated with altered modification of the activator of *MET* transcription, Met4 (Figures 4B, S4A). We propose that Met4 reacted to a nutritional deficiency rather than a signaling defect upon loss of Swi-Snf, as Met4 PTM patterns in *snfΔ* mutants responded to loss of methionine/cysteine in a manner similar to wild type cells (Figure 4B). In agreement with this result, ChIP data showed that Met4 occupancy was altered globally in *snfΔ* mutants, resulting in fewer peaks compared to YPD-grown wild type cells, but being more prominently recruited to genes involved in sulfur amino acid metabolism (Figure 4C–E). This reduction in peak number compared to a YPD-grown wild type was unexpected, but wild type cells grown in the absence of amino acids also display a reduction in peak number (compare Figure 5C to 4E). It was found that in both *snfΔ* mutants and SD-grown wild type cells, Met4-Myc was not only detected more prominently at metabolic genes, but was more focused upstream of TSS regions globally, with *snfΔ* mutants showing similar profiles to starved cells, at a slightly reduced scale (Figures 5D–G, S5E). In fact, in the case of both *MET* gene transcription and Met4 occupancy at *MET* promoters, *snfΔ* mutants tend to show an intermediate phenotype between YPD-grown and SD-grown wild type cells, displaying elevated transcription, altered Met4-ub levels and generally increased recruitment of Met4 to *MET* promoters (Figures 1F, 4B and 5A). However, in contrast to YPD or SD-grown wild type cells, *snfΔ* mutants show reduced Met4 binding both globally (Figure 5C) and specifically at several genes in the *MET* regulon (Figures 5A and S5C). This may suggest that Swi-Snf affects Met4 activity both indirectly through its influence on cysteine biosynthesis and directly by facilitating Met4 binding at some promoters.

Met4 occupancy specifically increased over the *MET* regulon in *snfΔ* mutants, and particularly over genes whose expression increased in these strains (Figure 5A,B). However, the presence of Met4 at several *MET* genes in wild type cells under repressing conditions may complicate these findings and suggests that this mechanism is more complex than currently understood (26,48). Similar levels of differentially-ubiquitinated Met4 bound to a promoter may have different effects on gene transcription, and the ability of these different species of protein to be recruited to target sites deserves further study. Even so, conditional depletion of Met4 via the anchor away technique significantly reduced activation of many *MET* genes in a *snf2Δ* mutant (Figure 6B). However, there is a subset of *MET* genes which are activated in response to loss of Swi-Snf, but do not appear to respond to Met4 loss. These genes are repressible by incubation in the presence of cysteine (Figure 2D), so we may speculate that they are redundantly activated in the absence of Swi-Snf in response to cysteine biosynthesis defects. It has been shown that several genes in the *MET* regulon are activated by transcription factors such as Yap1 and Gcn4, which may contribute to this phenomenon (50,51).

Our findings indicate that the elevated *MET* transcription in *snfΔ* mutants occurs following activation of Met4, which is not conserved in other non-yeast eukaryotes. However, work carried out on *Caenorhabditis elegans* has identified a factor similar in function to Met4, and also described differential regulation of genes involved in cysteine and methionine biosynthesis (52). This may indicate that while the proteins involved may not be direct orthologs, similar mechanisms have evolved to regulate sulfur metabolism transcription in many species. Mammalian SWI/SNF has been shown to be frequently mutated in cancer (7). Cancer cells with SWI/SNF mutations have also been shown to be especially sensitive to inhibition of GSH biosynthesis, demonstrating that SWI/SNF has a role in glutathione metabolism in humans (29). Recent work done in yeast has similarly suggested that maintaining redox balance, potentially through regulation of GSH levels is a conserved role of Swi-Snf (53).

**Figure 7.**
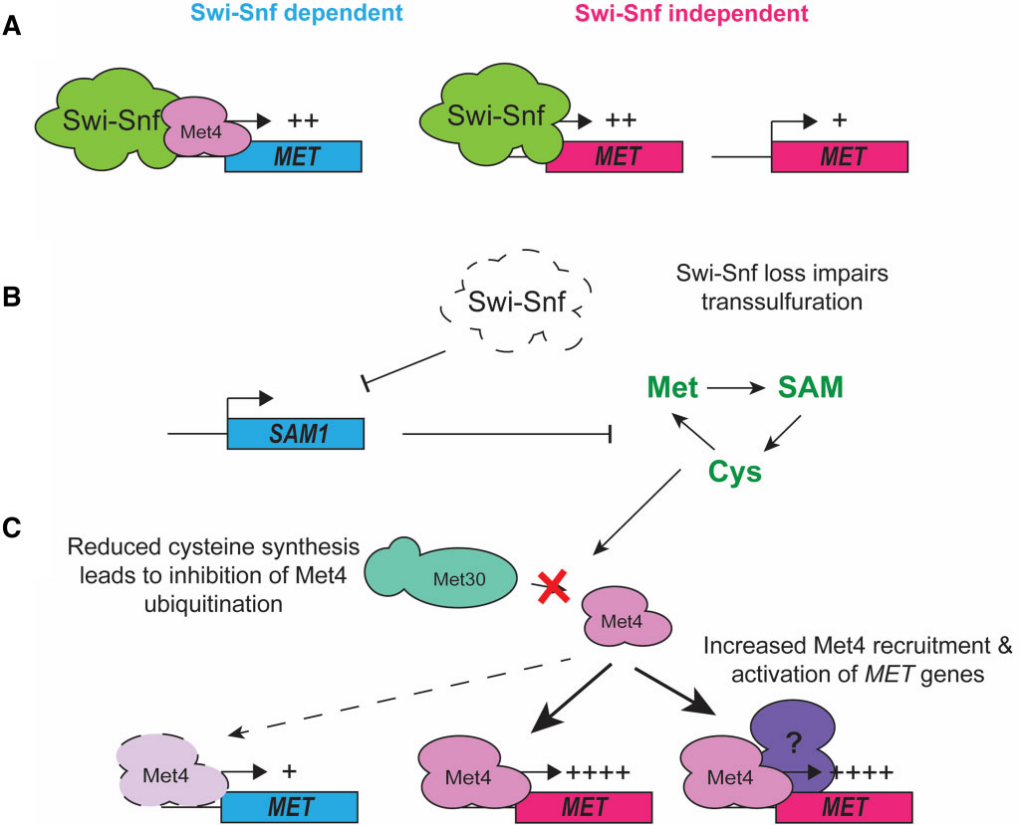
Model for Swi-Snf-mediated control of *MET* transcription in rich media. (**A**) During growth in rich media, Swi-Snf occupies both Swi-Snf-dependent and independent *MET* genes, with the former requiring both Swi-Snf and Met4 for full activity. (**B**) Loss of Swi-Snf results in repression of Swi-Snf-dependent *MET* genes, leading to a cysteine biosynthesis defect and reduced cysteine biosynthesis. (**C**) Cysteine starvation caused by Swi-Snf loss is sensed by cells, whereby Met4 is no longer ubiquitinated by SCF^Met30^, and can activate transcription of the *MET* regulon in cooperation with other transcriptional activators.

These data allow us to construct a model whereby in the presence of abundant nutrients, the *MET* regulon may be divided into Swi-Snf-dependent and independent genes. Based on Snf2 ChIP data from Dutta *et al*, Swi-Snf tends to be bound to highly expressed genes in both categories (Figures 7A, S5B) (38). Our own data suggest that one difference between Swi-Snf dependent and independent genes is Met4 activity during growth in rich media, with the latter showing no reduction in transcription in wild type cells following Met4-AA (Figure 5B). Upon loss of Swi-Snf, transcription of these Swi-Snf-dependent genes is reduced. Some of these genes (*SAM1*/*CYS4*) are critical to maintaining cysteine biosynthesis (Figure 7B). This reduction in cysteine biosynthesis is sensed by the cellular machinery, preventing ubiquitination of Met4, and active Met4 is recruited to its *MET* regulon targets, where it may activate the Swi-Snf independent genes (Figure 7C). This group includes genes that are dependent on Met4 alone for elevated transcription, and those which may be activated redundantly. Met4 may also be recruited to Swi-Snf dependent genes, but in the absence of Swi-Snf these genes are still repressed.

## Supplementary Material

gkad711_Supplemental_Files

## Data Availability

Original data underlying this manuscript can be accessed from the Stowers Original Data Repository at http://www.stowers.org/research/publications/libpb-1735. The datasets are available in the Gene Expression Omnibus (GEO) database under the accession number GSE197919.
