# Supplementary material for: The Swi-Snf chromatin remodeling complex mediates gene repression through metabolic control
Source: Nucleic Acids Res. 2023 Aug 31;51(19):10278–91. doi: 10.1093/nar/gkad711 (PMC10602859; doi:10.1093/nar/gkad711)
Supplement: gkad711_Supplemental_Files [file gkad711_supplemental_files.zip › MChurch Supplemental Figs R2.pdf]

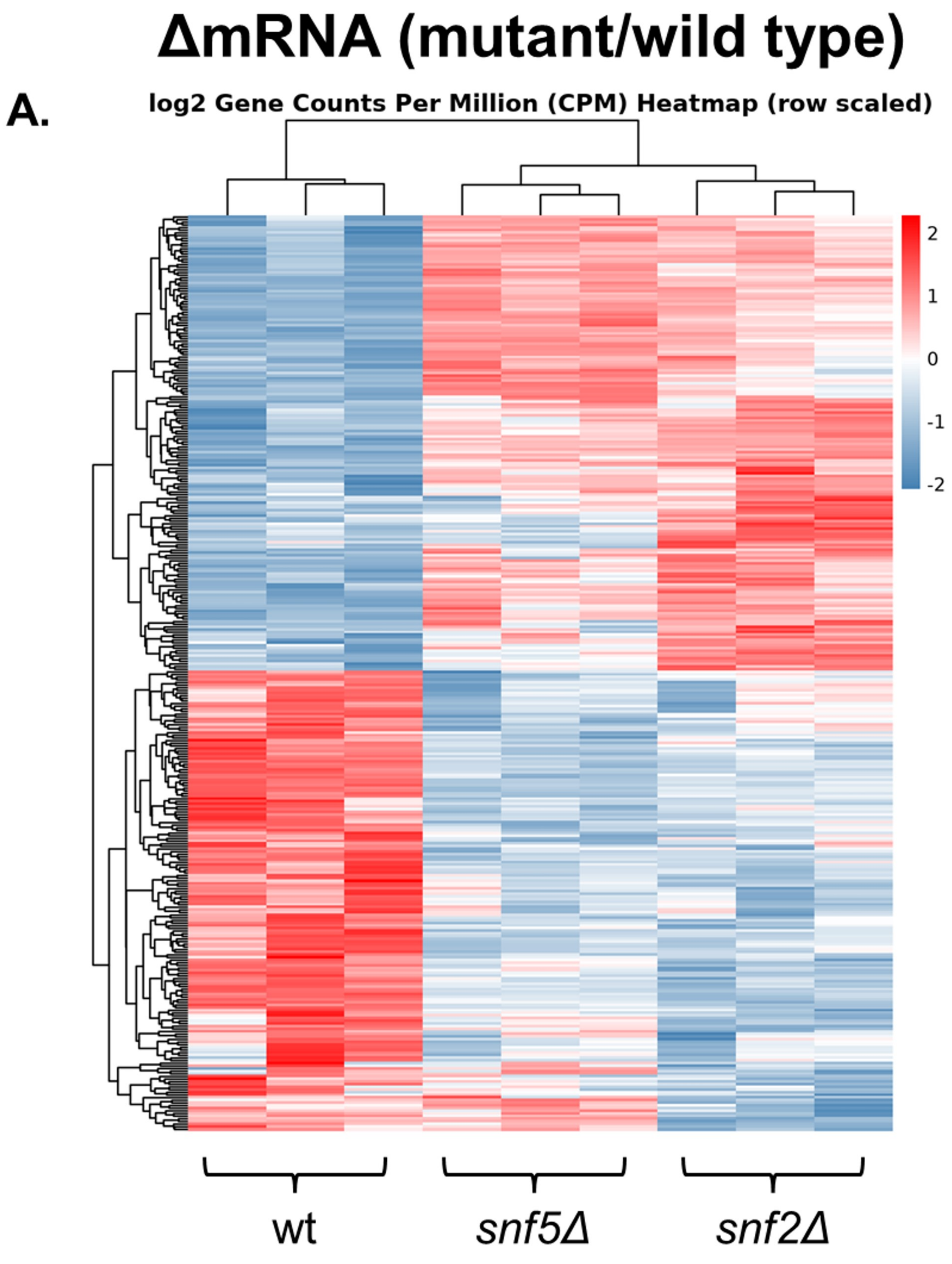

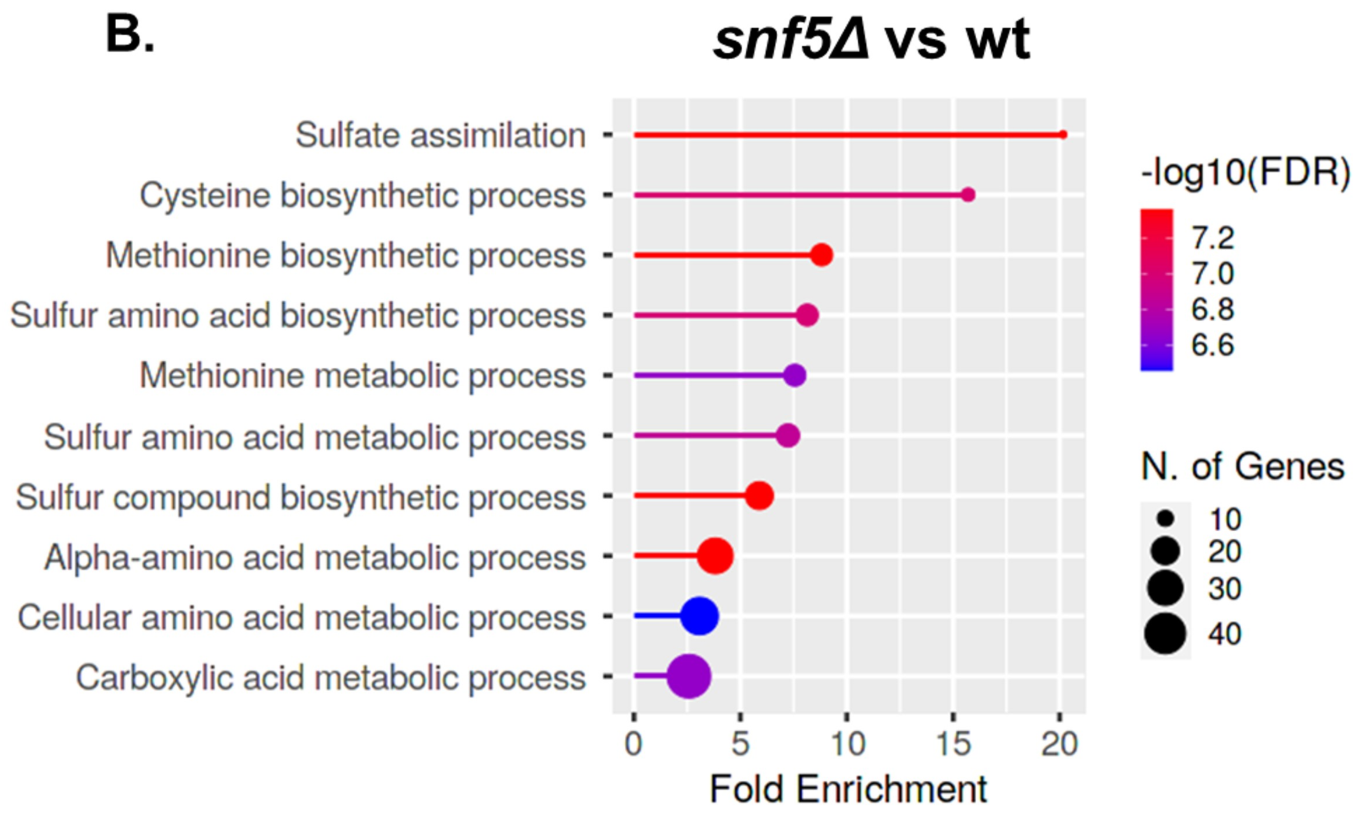

C.

Gene	Homolog	Disease	Reference
SAH1	AHCY	hyperhomocysteinemia	Visram M, et al. (2018)
CYS4	CBS	homocystinuria	Kruger WD and Cox DR (1994,1995); Mayfield JA, et al. (2012)
GSH2	GSS	Huntington's disease	Willingham S, et al. (2003)
ZWF1	G6PD	congenital nonspherocytic hemolytic anemia	Grabowska D, et al. (2004)

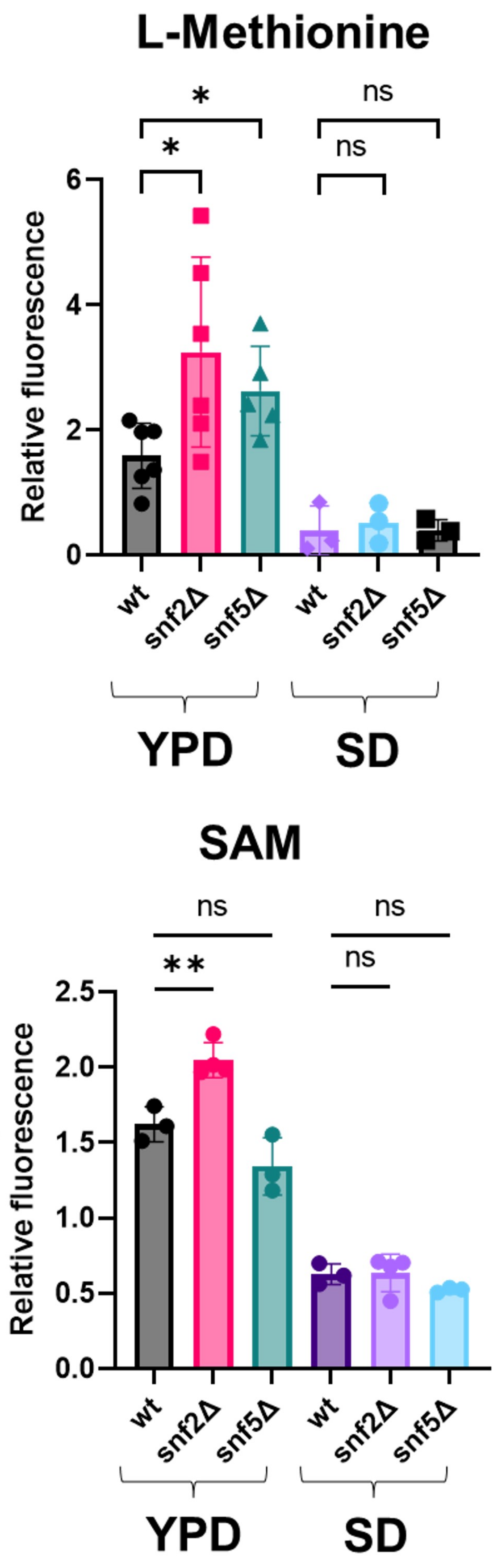

Figure S1 (A) log_2 cpm data from YPD-grown wild type (wt), *snf2Δ* and *snf5Δ* RNA-seq experiment. Transcripts found to be differentially-expressed vs wt shown. (B) GO term analysis of all DE-upregulated transcripts in YPD-grown *snfΔ* mutant vs wild type cells, analyzed using ShinyGO (Ge et al. 2020) (C) Sulfur metabolism genes in yeast and humans, and their role in disease (Kruger and Cox 1994; Kruger and Cox 1995; Willingham et al. 2003; Grabowska et al. 2004; Mayfield et al. 2012; Visram et al. 2018). (D) L-Methionine concentration in in YPD- and SD-grown wt vs snf*Δ* mutants. Error bars represent standard deviation from 5-6 independent experiments. (E) SAM concentration in in YPD-grown wt vs snf*Δ* mutants. Error bars represent standard deviation from 3 independent experiments. Concentration was plotted as relative fluorescence, calculated per manufacturer's instructions. p values indicated by asterisks, with a p value ≤ 0.05 being considered statistically significant (one asterisk) and p ≤ 0.01 being represented by two asterisks.

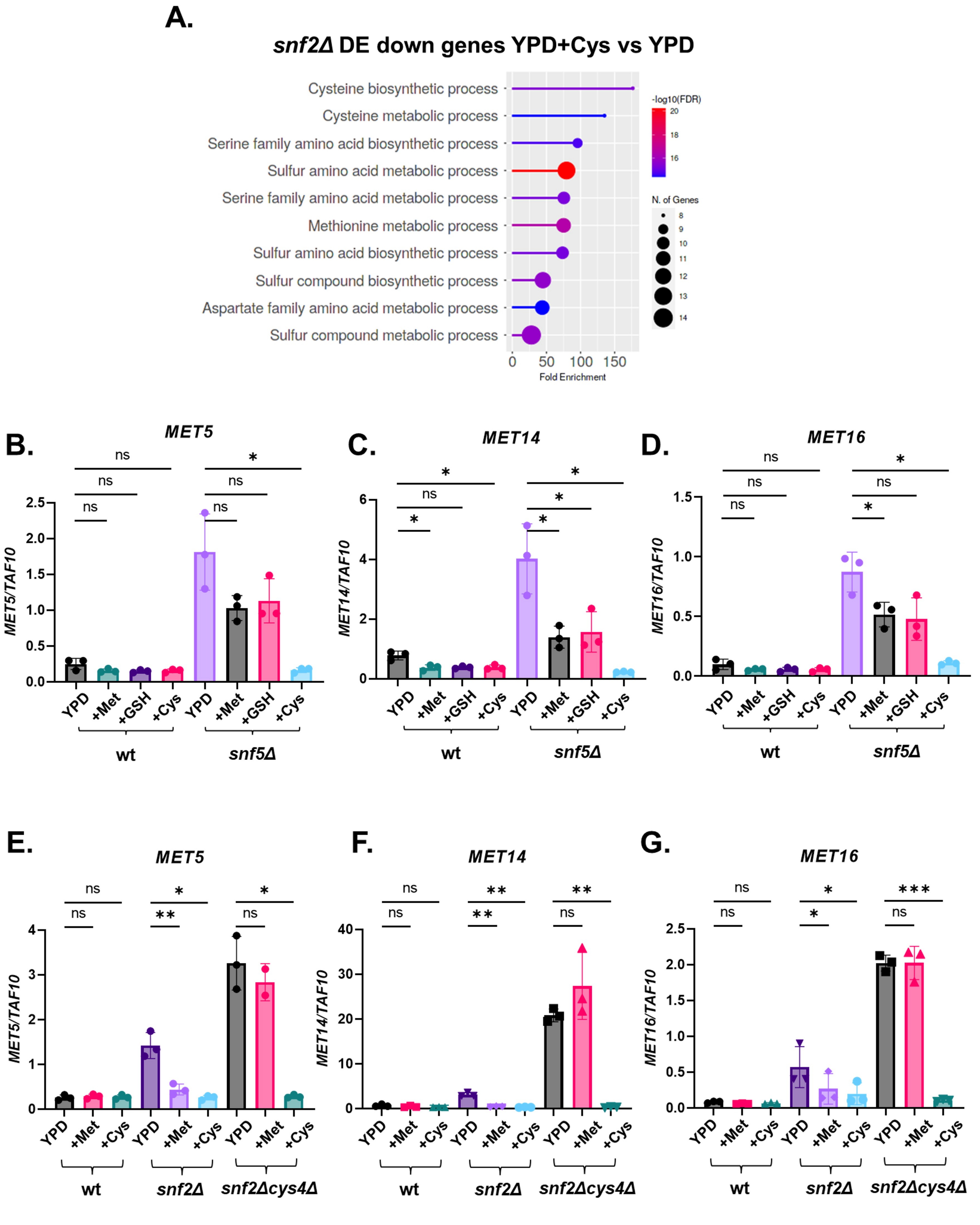

Figure S2 (A) GO term analysis of all DE- transcripts (down) in YPD- vs YPD+Cys-grown *snf2Δ* mutant cells, analyzed using ShinyGO (Ge et al. 2020) (B-D) RT-qPCR data showing (B) *MET5,* (C) *MET14* and (D) *MET16* transcription in wild type (wt) and *snf5Δ* strains after incubation in YPD, YPD containing 3 mM methionine (YPD + Met), 3 mM GSH (YPD + GSH) or 3 mM cysteine (YPD + Cys). (E-G) RT-qPCR data showing (B) *MET5,* (C) *MET14* and (D) *MET16* transcription in wild type (wt), *snf2Δ* and *snf2Δcys4Δ* strains after incubation in YPD, YPD containing 3 mM methionine (YPD + Met), or 3 mM cysteine (YPD + Cys). Transcript levels normalized to *TAF10*. Error bars represent standard deviation from 2-3 independent experiments. p values indicated by asterisks, with a p value ≤ 0.05 being considered statistically significant (one asterisk), p ≤ 0.01 being represented by two asterisks and p ≤ 0.001 being represented by three asterisks.

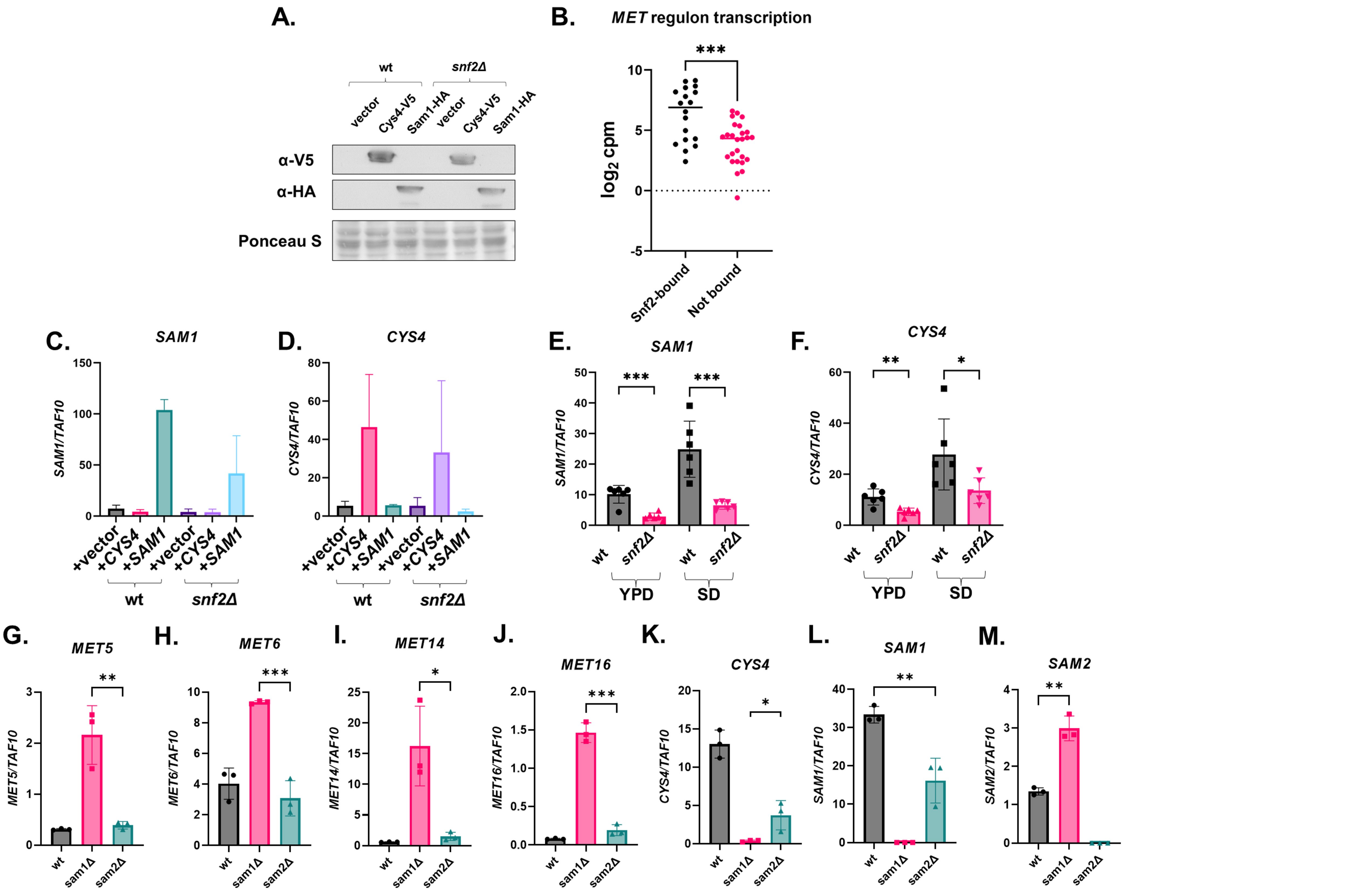

Figure S3 (A) Western blot of HA-tagged Sam1 & V5-tagged Cys4 in SC-Ura-grown wild type and *snf2Δ* mutant cells. Ponceau S used as a loading control. (B) log_2cpm transcription of *MET* genes from RNA seq data from this study, plotted and grouped by whether or not the gene promoter was bound or not bound by Swi-Snf, based on ChIP data from (Dutta et al. 2014). (C & D) RT-qPCR data showing (C) *SAM1* (D) or *CYS4* transcription in wild type (wt) and *snf2Δ* strains containing a pRS416 vector or plasmids overexpressing *CYS4* or *SAM1* using a *TEF1* promotor. (E) RT-qPCR data showing *SAM1* transcription in rich (YPD) or minimal (SD) media in wild type vs *snf2Δ* mutant cells (F) RT-qPCR data showing *CYS4* transcription in rich (YPD) or minimal (SD) media in wild type vs *snf2Δ* mutant cells Transcript levels normalized to *TAF10*. p values indicated by asterisks, with a p value ≤ 0.05 being considered statistically significant (one asterisk). (G-M) RT-qPCR data showing (G) *MET5,* (H) *MET6,* (I) *MET14,* (J) *MET16,* (K) *CYS4* (L) *SAM1* and (M) *SAM2* transcription in wild type (wt), *sam1Δ* and *sam2Δ* strains. Transcript levels normalized to *TAF10*. p values indicated by asterisks, with a p value ≤ 0.05 being considered statistically significant (one asterisk), p ≤ 0.01 being represented by two asterisks and p ≤ 0.001 being represented by three asterisks.

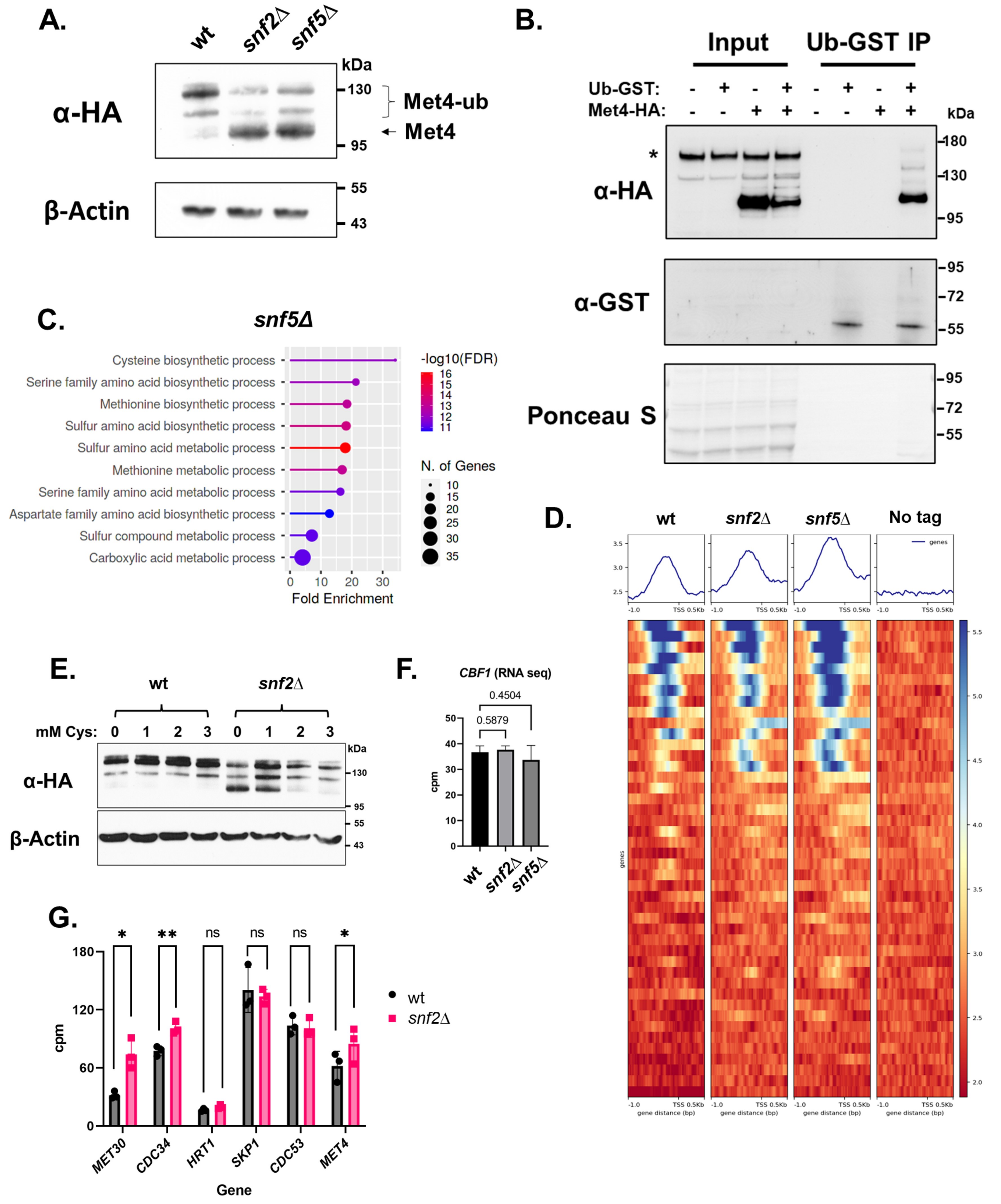

Figure S4 (A) Western blot of HA-tagged Met4 (Met4-HA) in wild type, *snf2Δ* and *snf5Δ* strains grown in YPD. β-Actin used as a loading control. (B) Western blot of Ub-GST pulldown in untagged, Ub-GST, Met4-3HA and Met4-3HA Ub-GST cells. (C) GO term analysis for genes occupied by Met4-13Myc in YPD-grown *snf5Δ* mutant cells, as determined by IDR analysis. (D) Heatmap and line plot of log2 Met4-13Myc occupancy -1000 bp/+500 bp relative to the TSS of *MET* regulon genes in wild type (wt), *snf2Δ* & *snf5Δ* strains, in addition to an untagged wild type (No tag). Heatmaps displaying the average distribution of reads around Transcriptional Start Sites (-1kb/+0.5kb) for each sample group were generated using deeptools (2.7.5) with WiggleTools (1.2) used to calculate the mean bigwig coverage across sample replicates. (E) Western blot of 3HA-tagged Met4 (Met4-HA) in wild type and *snf2Δ* mutants, grown either in YPD with no supplemental cysteine (0), or with 1, 2 or 3 mM cysteine added to media prior to 30 minutes' incubation. β-Actin used as a loading control.(F) RNA-seq data from YPD-grown wild type, *snf2Δ* and *snf5Δ* mutants monitoring transcript levels (cpm) of *CBF1* (G) RNA-seq data from YPD-grown wild type and *snf2Δ* mutants monitoring transcript levels (cpm) of *MET30*, *CDC34*, *HRT1*, *SKP1*, *CDC53* and *MET4*. p values indicated by asterisks, with a p value ≤ 0.05 being considered statistically significant (one asterisk) and p ≤ 0.01 being represented by two asterisks

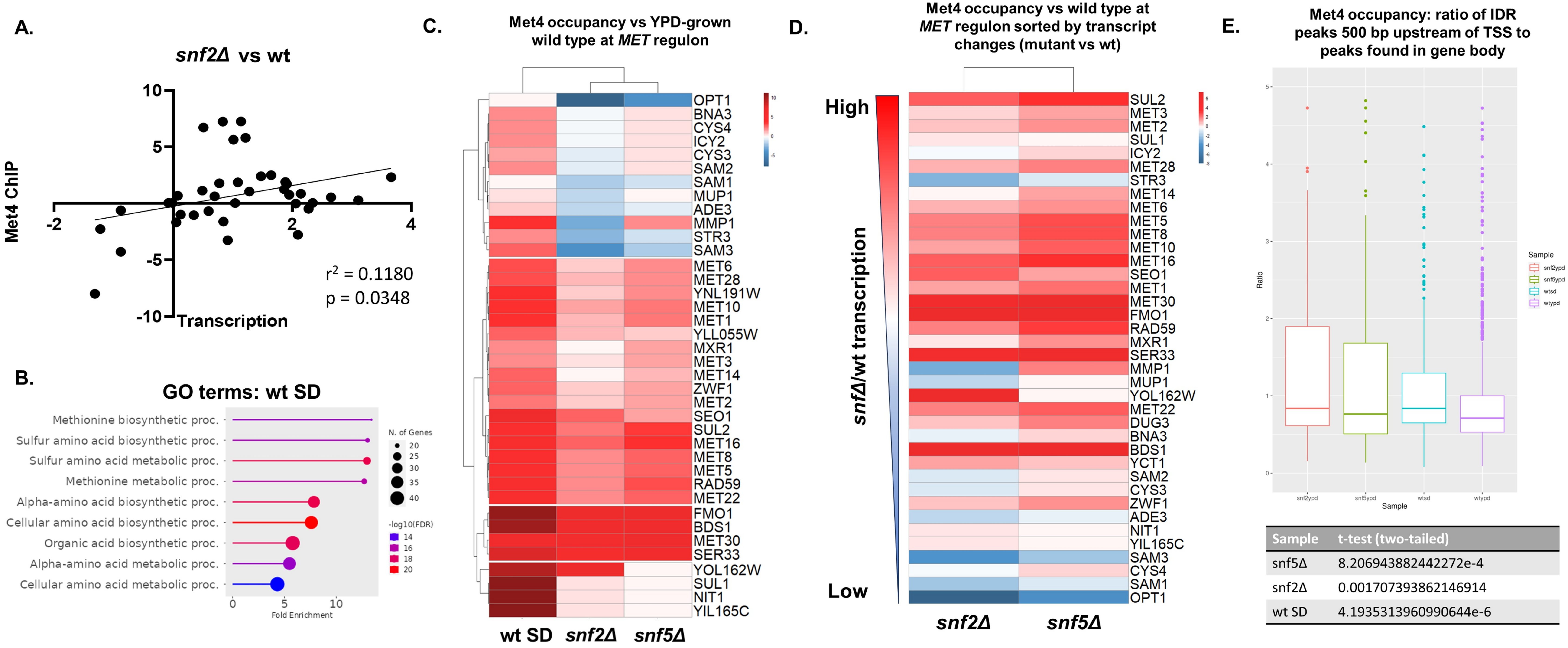

Sample	t-test (two-tailed)
snf5Δ	8.206943882442272e-4
snf2Δ	0.001707393862146914
wt SD	4.1935313960990644e-6

Figure S5 (A) Scatterplot of *snf2Δ* vs wild type (wt) comparing log2fc transcription data based on RNA-seq (x-axis) to Met4 occupancy changes (y-axis from Met4-Myc ChIP seq dataset using peaks called using DiffBind.. (B) GO term analysis for genes occupied by Met4-13Myc in SD-grown wild type cells, as determined by IDR analysis. (C) Heatmap representing log2 fold-change Met4-Myc occupancy vs wild type of *MET* regulon genes in SD-grown wild type and YPD-grown *snf2Δ* and *snf5Δ* mutants from a ChIP-seq experiment using peaks called using DiffBind. (D) Heatmap representing log2 fold-change Met4-Myc occupancy vs wild type of *MET* regulon genes in YPD-grown *snf2Δ* and *snf5Δ* mutants from a ChIP-seq experiment using peaks called using DiffBind, ordered based on transcriptional changes (RNA-seq) in mutants vs wild type. (E) Boxplot showing ratio of genome-wide Met4-Myc peak enrichment -500 bp upstream of TSS compared to enrichment in gene bodies for YPD and SD-grown wild type and YPD-grown *snf2Δ* and *snf5Δ* mutants based on peaks determined by IDR for each strain, with p-values for each comparison to YPD-grown wild type in the table underneath.

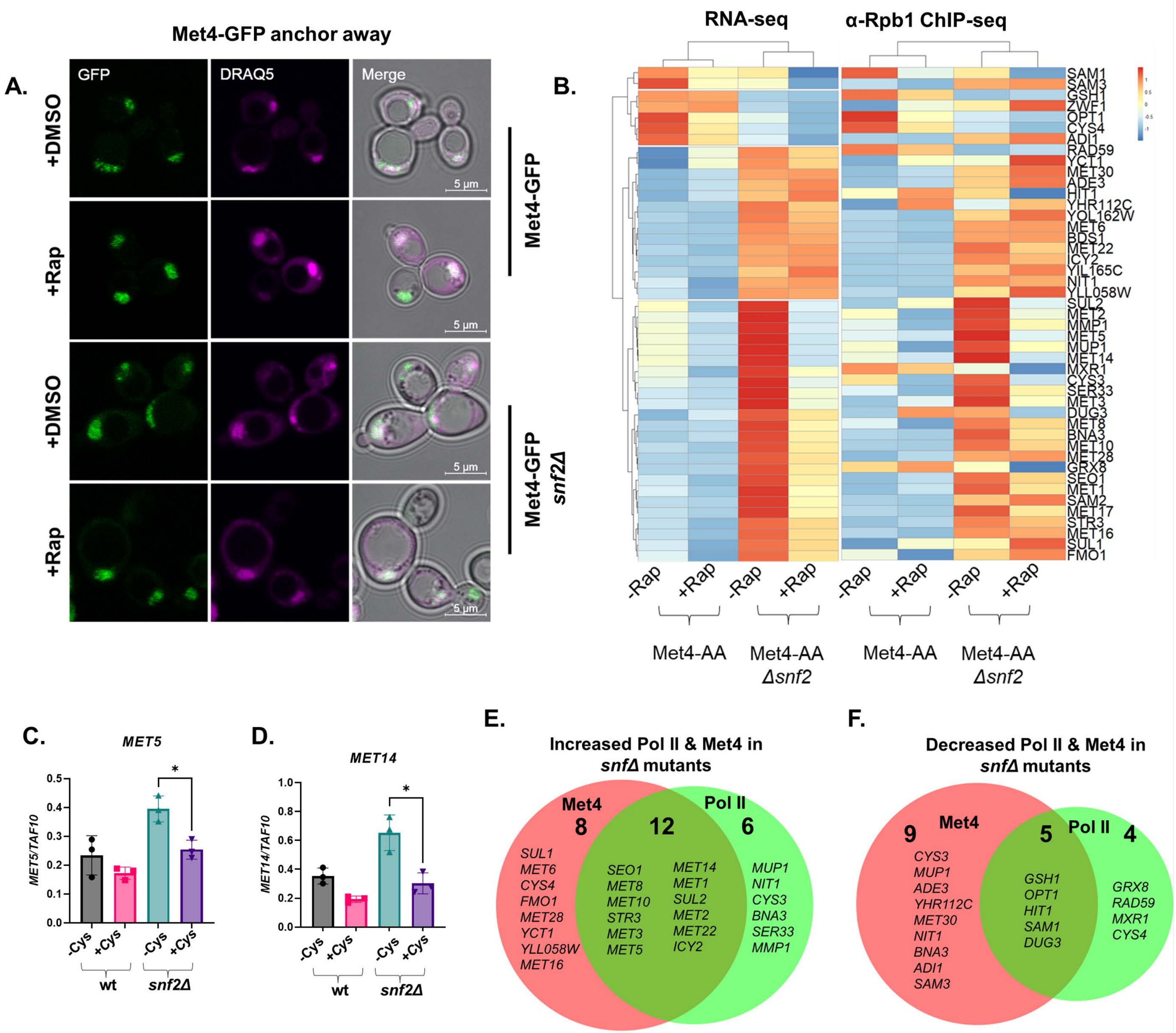

Figure S6 (A) Comparison of Met4-GFP localization between wild type and *snf2Δ* strains following incubation for 1h in 1 µg/mL Rapamycin (+Rap) or an equivalent volume of DMSO (+DMSO). (B) Heatmap of YPD-Met4-AA and Met4-AA *snf2Δ* strains without (-Rap) or with (+Rap) incubation in the presence of 1 µg/mL rapamycin incubation showing separately row-scaled transcript and Rpb1 levels at *MET* regulon genes, clustered based on RNA-seq data. (C-D) RT-qPCR data showing (C) *MET5* and (D) *MET14* transcription in wild type (wt) and *snf2Δ* strains in a HHY221 background after incubation in YPD or YPD with 3 mM cysteine (YPD + Cys). Transcript levels normalized to *TAF10*. Error bars represent standard deviation from three independent experiments. p values indicated by asterisks, with a p value ≤ 0.05 being considered statistically significant (one asterisk). (E) Venn diagram showing overlap between *MET* genes where Pol II levels increase in a *snf2Δ* mutant vs wild type in a Met4-dependent manner and those genes showing increased Met4-Myc occupancy in *snfΔ* mutants based on data from Fig. 5A. (F) Venn diagram showing overlap between *MET* genes where Pol II levels decrease in a *snf2Δ* mutant vs wild type and those genes showing decreased Met4-Myc occupancy in *snfΔ* mutants based on data from Fig. 5A.

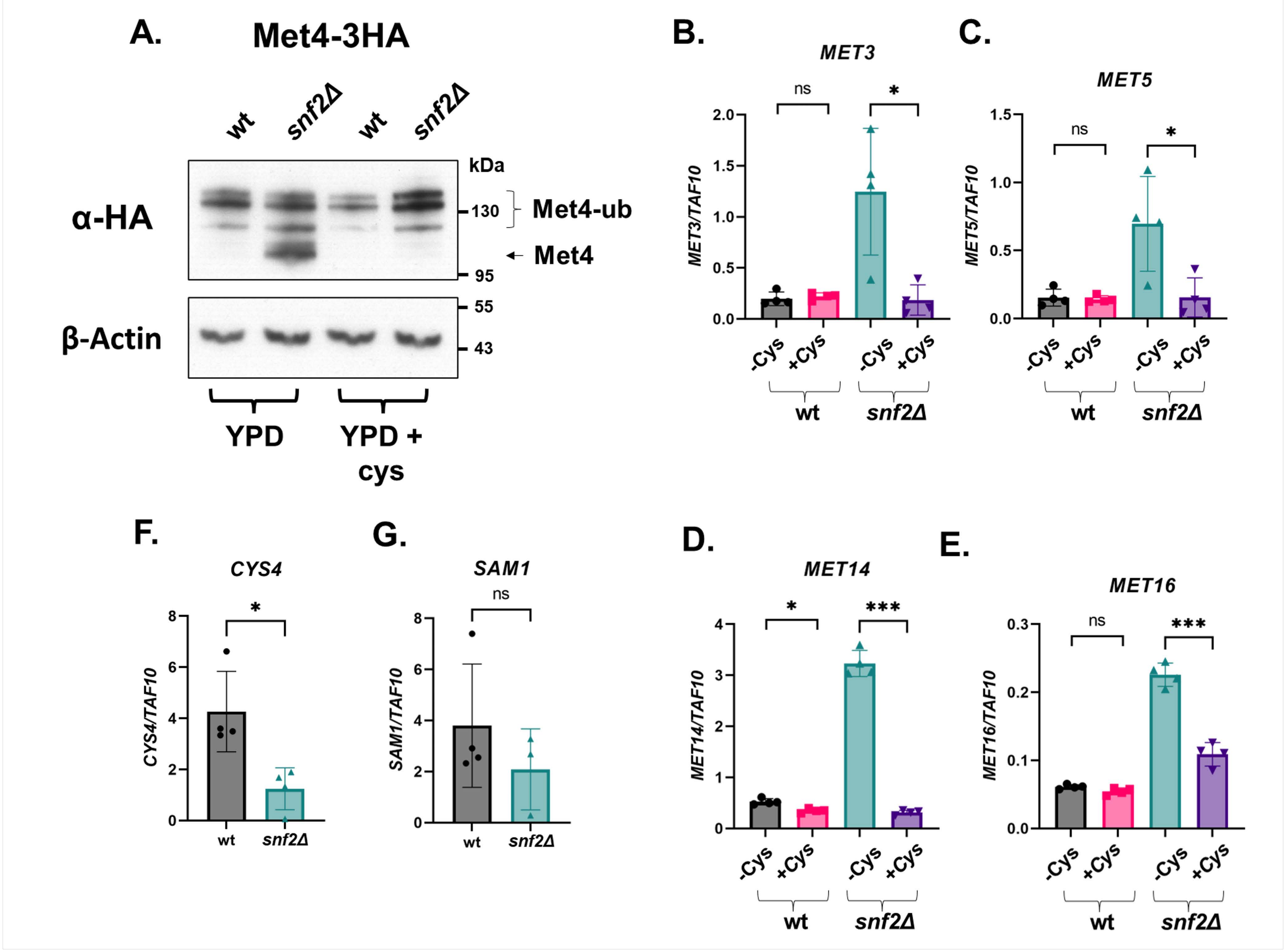

Figure S7 (A) Western blot of 3HA-tagged Met4 (Met4-HA) in wild type and *snf2Δ* mutants in a BY4742 background, grown either in YPD with no supplemental cysteine (YPD) or after incubation for 30 minutes in YPD with 3 mM cysteine (YPD+cys). β-Actin used as a loading control. (B-E) RT-qPCR data showing (B) *MET3*, (C) *MET5*, (D) *MET14*, and (E) *MET16* transcription in wild type (wt) and *snf2Δ* strains in a BY4742 background after incubation in YPD or YPD with 3 mM cysteine (YPD + Cys). (F) *CYS4* and (G) *SAM1* transcription in wild type (wt) and *snf2Δ* strains in a BY4742 background. Transcript levels normalized to *TAF10*. Error bars represent standard deviation from four independent experiments. p values indicated by asterisks, with a p value ≤ 0.05 being considered statistically significant (one asterisk) and p ≤ 0.001 being represented by three asterisks.
