# Supplementary material for: The Swi-Snf chromatin remodeling complex mediates gene repression through metabolic control
Source: Nucleic Acids Res. 2023 Aug 31;51(19):10278–91. doi: 10.1093/nar/gkad711 (PMC10602859; doi:10.1093/nar/gkad711)
Supplement: gkad711_Supplemental_Files [file gkad711_supplemental_files.zip › Supplemental_Methods.docx]

**Supplementary Materials and Methods**

**RNA-seq analysis**.

For RNA-seq analysis, raw reads were demultiplexed into FASTQ format allowing up to one mismatch using Illumina bcl2fastq2 v2.18. The fastq files were aligned to the sacCer3 assembly from UCSC with Ensembl 98 annotations using STAR (version STAR_2.7.3a) (Dobin et al., 2013). TPM expression values were generated using RSEM (version v1.3.0) [5]. edgeR (version 3.24.3 with R 3.6.1) was applied to perform differential expression analysis (Robinson et al., 2009). A cutoff of FDR less than 0.05 and absolute value of log2 fold change greater than 2 was used to identify genes with statistically significant differential expression between two conditions. Differentially expressed genes were uploaded to Shiny GO to find overrepresented Gene Ontology terms.

**ChIP-seq analysis**.

For the Met4-Myc ChIP-seq analysis, reads were aligned to yeast genome sacCer3 using bowtie2 (2.3.4.1) with default settings. ChIP tracks were created using rpm normalized bigwig files for visualization. Peaks were called using MACS2 (2.1.2) with the nomodel argument and the input files as baseline controls. For the Met4-Myc ChIP-seq data, MACS2 peaks were then analyzed with IDR (2.0.3) to generate a list of peaks statistically consistent across replicates for a given sample group. For the Met4 ChIP-seq data, DiffBind (2.10.0) was run on the bam files and MACS2 peaks of various samples to find regions of differential binding between sample groups. Statistically significant peaks were then intersected with gene coordinates using BEDTools (2.29.0). For each MET Regulon gene, a promoter score was generated by dividing the average bigwig coverage in a 500 base window immediately upstream of the TSS by the average coverage over the corresponding gene body. These averages were calculated using the bigWigAverageOverBed (version 4) function from ENCODE. A Student’s t-test was run to test for a significant difference in promoter score between each mutant and WT. Plots displaying the coverage profile around TSS were generated using Deeptools (3.5.1)

For the Rpb1 ChIP-seq analysis, bowtie2 (2.4.2) was used. RPM-normalized bigwig files were generated using Deeptools’ bamCoverage with binSize set to 1. An average coverage score for every gene was calculated from the average bigwig coverage across the gene body. This value was then averaged across replicates within each sample group.
