# Supplementary material for: The Swi-Snf chromatin remodeling complex mediates gene repression through metabolic control
Source: Nucleic Acids Res. 2023 Aug 31;51(19):10278–91. doi: 10.1093/nar/gkad711 (PMC10602859; doi:10.1093/nar/gkad711)
Supplement: gkad711_Supplemental_Files [file gkad711_supplemental_files.zip › Supplemental_Table_S1.doc.docx]

**Table S1: Strains used in this study**

| Strain | Genotype | Source |
| --- | --- | --- |
| BY4741 | *MATa his3Δ1 leu2Δ0 met17Δ0 ura3Δ0* | [1] |
| BY4742 | *MATα his3Δ1 leu2Δ0 lys2Δ0 ura3Δ0* | [1] |
| HHY221 | *W303 MATa TOR1-1 fpr1∆::loxP-LEU2-loxP rpl13a::RPL13A-2×FKBP12-loxP* | [2] |
| YMC103 | *MATa his3Δ1 leu2Δ0 met17Δ0 ura3Δ0 cys4Δ::URA3* | This study |
| YMC119 | *W303 MATa TOR1-1 fpr1∆::loxP-LEU2-loxP rpl13a::RPL13A-2×FKBP12-loxP snf2Δ::natMX6* | This study |
| YMC122 | *MATa his3Δ1 leu2Δ0 met17Δ0 ura3Δ0 +pRS416* | This study |
| YMC124 | *MATa his3Δ1 leu2Δ0 met17Δ0 ura3Δ0 snf2Δ::kan +pRS416* | This study |
| YMC126 | *MATa his3Δ1 leu2Δ0 met17Δ0 ura3Δ0 +pMC21* | This study |
| YMC130 | *MATa his3Δ1 leu2Δ0 met17Δ0 ura3Δ0 +pMC13* | This study |
| YMC135 | *MATa his3Δ1 leu2Δ0 met17Δ0 ura3Δ0 snf2Δ::kan +pMC13* | This study |
| YMC136 | *MATa his3Δ1 leu2Δ0 met17Δ0 ura3Δ0 snf2Δ::kan +pMC21* | This study |
| YMC138 | *W303 MATa TOR1-1 fpr1∆::loxP-LEU2-loxP rpl13a::RPL13A-2×FKBP12-loxP Met4-FRB-GFP::HIS3* | This study |
| YMC139 | *W303 MATa TOR1-1 fpr1∆::loxP-LEU2-loxP rpl13a::RPL13A-2×FKBP12-loxP Met4-GFP::HIS3* | This study |
| YMC140 | *W303 MATa TOR1-1 fpr1∆::loxP-LEU2-loxP rpl13a::RPL13A-2×FKBP12-loxP Met4-FRB-GFP::HIS3 snf2Δ::KanMX6* | This study |
| YMC81 | *MATa his3Δ1 leu2Δ0 met17Δ0 ura3Δ0 MET4-13XMyc::HIS* | This study |
| YMC88 | *MATa his3Δ1 leu2Δ0 met17Δ0 ura3Δ0 MET4-13XMyc::HIS snf5Δ::URA* | This study |
| YMC95 | *MATa his3Δ1 leu2Δ0 met17Δ0 ura3Δ0 MET4-3XHA::Kan* | This study |
| YMC96 | *MATa his3Δ1 leu2Δ0 met17Δ0 ura3Δ0 MET4-3XHA::Kan snf5Δ::URA* | This study |
| YMC98 | *MATa his3Δ1 leu2Δ0 met17Δ0 ura3Δ0 MET4-3XHA::Kan snf2Δ::URA* | This study |
| YMC99 | *MATa his3Δ1 leu2Δ0 met17Δ0 ura3Δ0 MET4-13XMyc::HIS snf2Δ::URA* | This study |
| YMC105 | *MATa his3Δ1 leu2Δ0 met17Δ0 ura3Δ0 snf2Δ::kan cys4Δ::URA* | This study |
| *gsh1Δ* | *MATa his3Δ1 leu2Δ0 met17Δ0 ura3Δ0 gsh1Δ::KanMX4* | Saccharomyces Genome Deletion Project (SGDP) |
| *opt1Δ* | *MATa his3Δ1 leu2Δ0 met17Δ0 ura3Δ0 opt1Δ::KanMX4* | Saccharomyces Genome Deletion Project (SGDP) |
| *sam1Δ* | *MATa his3Δ1 leu2Δ0 met17Δ0 ura3Δ0 sam1Δ::KanMX4* | Saccharomyces Genome Deletion Project (SGDP) |
| *sam3Δ* | *MATa his3Δ1 leu2Δ0 met17Δ0 ura3Δ0 sam3Δ::KanMX4* | Saccharomyces Genome Deletion Project (SGDP) |
| *snf2Δ* | *MATa his3Δ1 leu2Δ0 met17Δ0 ura3Δ0 snf2Δ::KanMX4* | Saccharomyces Genome Deletion Project (SGDP) |
| *snf5Δ* | *MATa his3Δ1 leu2Δ0 met17Δ0 ura3Δ0 snf5Δ::KanMX4* | Saccharomyces Genome Deletion Project (SGDP) |
| *sam2Δ* | *MATa his3Δ1 leu2Δ0 met17Δ0 ura3Δ0 sam2Δ::KanMX4* | Saccharomyces Genome Deletion Project (SGDP) |
| *YMC150* | *MATα his3Δ1 leu2Δ0 lys2Δ0 ura3Δ0 MET4-3HA::KanMX4* | This study |
| *YMC154* | *MATα his3Δ1 leu2Δ0 lys2Δ0 ura3Δ0 MET4-3HA::KanMX4 snf2Δ::URA* | This study |
| *YMC153* | *MATα his3Δ1 leu2Δ0 lys2Δ0 ura3Δ0 snf2Δ::URA* | This study |
| *YMC160* | *MATa his3Δ1 leu2Δ0 met17Δ0 ura3Δ0*  *UBI4-GST::HIS3MX6* | This Study |
| *YMC161* | *MATa his3Δ1 leu2Δ0 met17Δ0 ura3Δ0*  *UBI4-GST::HIS3MX6 MET4-3HA::KanMX4* | This Study |

1. Brachmann, C.B., et al., *Designer deletion strains derived from Saccharomyces cerevisiae S288C: a useful set of strains and plasmids for PCR-mediated gene disruption and other applications.* Yeast, 1998. **14**(2): p. 115-32.

2. Haruki, H., J. Nishikawa, and U.K. Laemmli, *The anchor-away technique: rapid, conditional establishment of yeast mutant phenotypes.* Mol Cell, 2008. **31**(6): p. 925-32.
