# Supplementary material for: The Swi-Snf chromatin remodeling complex mediates gene repression through metabolic control
Source: Nucleic Acids Res. 2023 Aug 31;51(19):10278–91. doi: 10.1093/nar/gkad711 (PMC10602859; doi:10.1093/nar/gkad711)
Supplement: gkad711_Supplemental_Files [file gkad711_supplemental_files.zip › Supplemental_Table_S2.docx]

**Table S2: Plasmids used in this study**

| Plasmid | Descripton | Source | Notes |
| --- | --- | --- | --- |
| pMC13 | pRS416 with XhoI-TEF1 promoter -BamHI-CYS4-V5-NotI | This Study | *CYS4* overexpression vector |
| pMC21 | pRS416 with XhoI-TEF1 promoter -BamHI-SAM1-HA-NotI | This Study | *SAM1* overexpression vector |
| pMC23 | pRS406 with NheI-FRB-GFP-AscI | This Study | Used for tagging AA-GFP strains |
| pRS416 | Commercially-available vector | (Sikorski and Hieter 1989) | Empty vector for overexpression experiments |
| pRS401 | Commercially-available vector | (Brachmann et al. 1998) | Used to restore *MET17* in BY4741 |
| pRS406 | Commercially-available vector | (Sikorski and Hieter 1989) | Used to generate *cys4::URA3* deletion |
| pFA6a-13Myc-His3MX6 | Commercially-available vector | (Longtine et al. 1998) | Used to Myc-tag Met4 |
| pFA6a-3HA-KanMX6 | Commercially-available vector | (Longtine et al. 1998) | Used to HA-tag Met4 |
| pFA6a-GST-His3MX6 | Commercially-available vector | (Longtine et al. 1998) | Used to GST-tag Ubi4 |
