# Supplementary material for: The Swi-Snf chromatin remodeling complex mediates gene repression through metabolic control
Source: Nucleic Acids Res. 2023 Aug 31;51(19):10278–91. doi: 10.1093/nar/gkad711 (PMC10602859; doi:10.1093/nar/gkad711)
Supplement: gkad711_Supplemental_Files [file gkad711_supplemental_files.zip › Supplemental_Table_S3.docx]

**Table S3: RT-qPCR primers used in this study**

| Name | Sequence | Notes |
| --- | --- | --- |
| TAF10 RT-F | ATATTCCAGGATCAGGTCTTCCGTAGC | From [1] |
| TAF10 RT-R | GTAGTCTTCTCATTCTGTTGATGTTGTTGTTG | From [1] |
| MET3 5'-F | TCGAGATTAGCAGACGGCAC |  |
| MET3 5'-R | AGGGCAATTCTTGTGTCTGGT |  |
| MET5_5'-F | CCTCTTGACGCTCCCACAAT |  |
| MET5_5'-R | GTGGCAACAGATTCTAGCCCT |  |
| MET14 5'-F | GGCATTGAGAAAACAGGACGG |  |
| MET14 5'-R | AACTGTTCTAGCGCACAGGC |  |
| Met16_5'-F | CACTTTGCACCATTTCCCACA |  |
| Met16_5'-R | CTGCCTCCGATTCACATCCA |  |
| CYS4RT-F | ATCGGCGCCATCAAAGGTTA |  |
| CYS4RT-R | GATTTCAGCACCCAGAGCCT |  |
| SAM1 5'-F | GTTTAGCCGAGGACCCTCAC |  |
| SAM1 5'-R | TCCAACTGTGCCTTGGTAGT |  |
| SAM2 5'-F | CGATTCTGCCAAGGGTTTCG |  |
| SAM2 5'-R | CCTTGGTCACCAGCACCTAA |  |
| MET2prom-F | ATTTCTTGCTATTGTTAGTGGCTCC | From [2] |
| MET2prom-R | CAACGAAGCGGAAGCTCATCTATT | From [2] |
| MET5prom-F | TTACCACCACACAGAGGCAG |  |
| MET5prom-R | TCGTCGCATCTCCATGACTC |  |
| MET6prom-F | AAGCAAGCATCTAAGAGCATTGAC | From [3] |
| MET6prom-R | TGAGTTCTCAAATCCTTACCGACC | From [3] |

1. Teste, M.A., et al., *Validation of reference genes for quantitative expression analysis by real-time RT-PCR in Saccharomyces cerevisiae.* BMC Mol Biol, 2009. **10**: p. 99.

2. Leroy, C., L. Cormier, and L. Kuras, *Independent recruitment of mediator and SAGA by the activator Met4.* Mol Cell Biol, 2006. **26**(8): p. 3149-63.

3. Ansari, S.A., et al., *Mediator, TATA-binding protein, and RNA polymerase II contribute to low histone occupancy at active gene promoters in yeast.* J Biol Chem, 2014. **289**(21): p. 14981-95.
