# Supplementary material for: The Swi-Snf chromatin remodeling complex mediates gene repression through metabolic control
Source: Nucleic Acids Res. 2023 Aug 31;51(19):10278–91. doi: 10.1093/nar/gkad711 (PMC10602859; doi:10.1093/nar/gkad711)

Supplementary file S1

Scale = 0-250

Additional images represent scale of 0-50

Wild type

snf2Δ

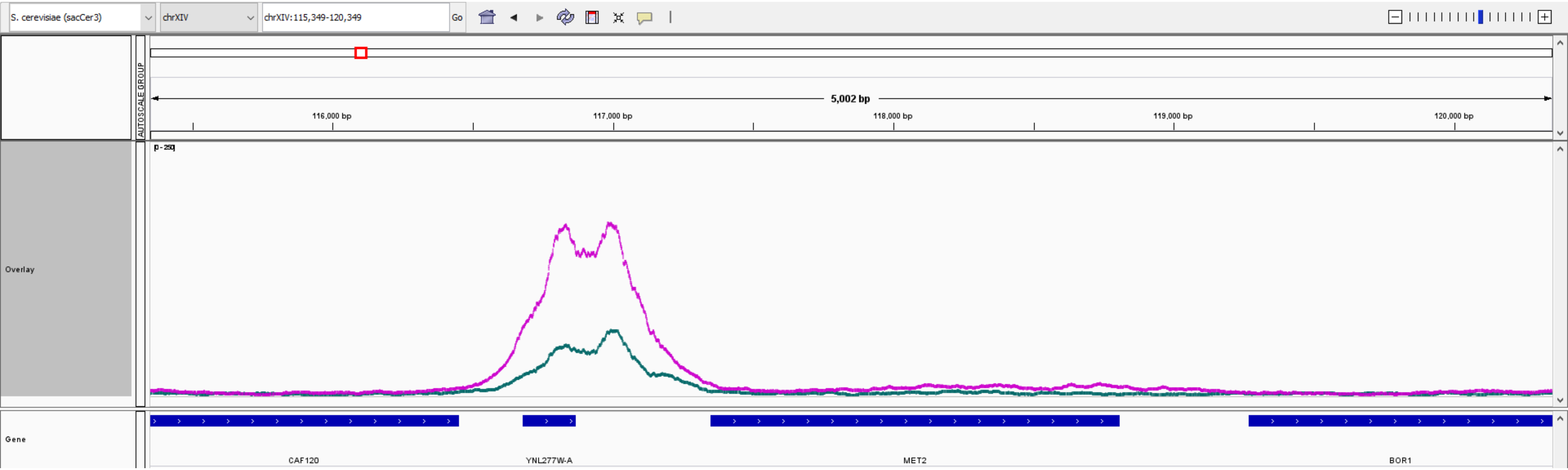

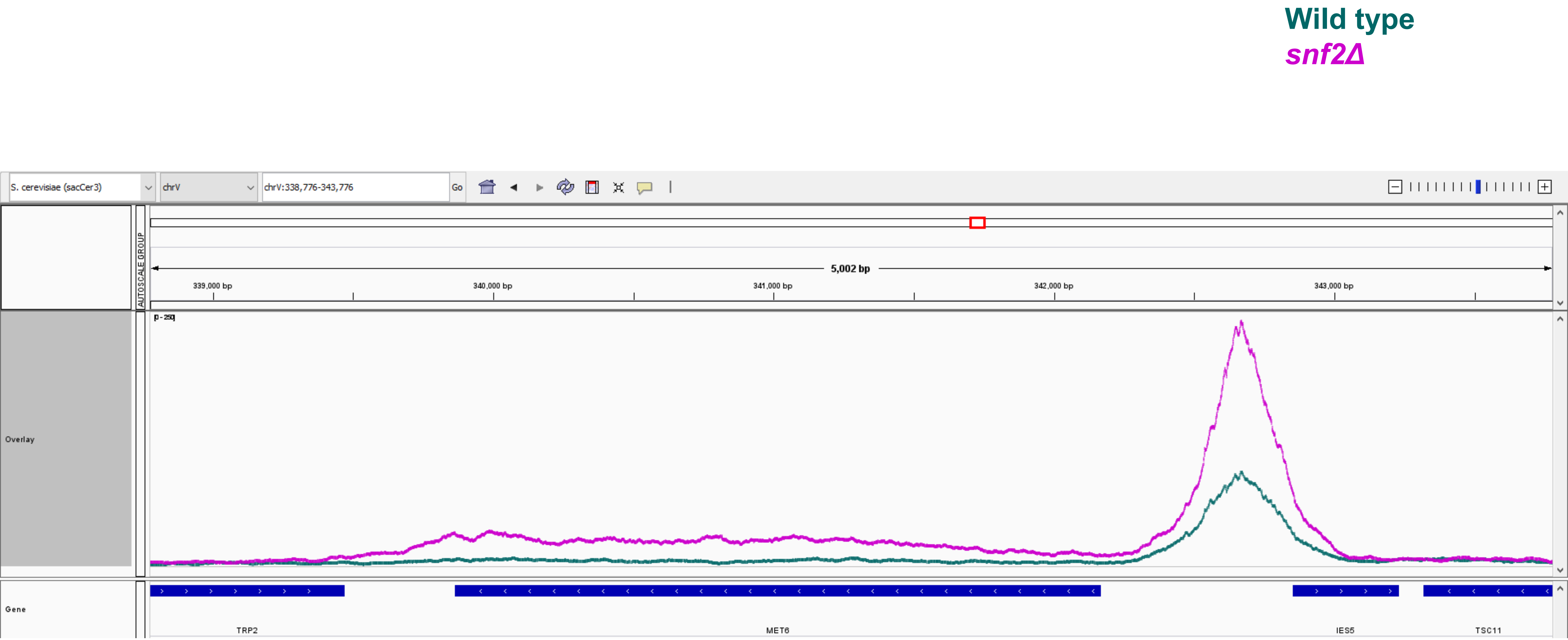
Wild type
snf2Δ
S. cerevisiae (sacCer3)
chrV
chrV:338,776-343,776
Go
AUTOSCALE GROUP
5,002 bp
339,000 bp
340,000 bp
341,000 bp
342,000 bp
343,000 bp
Overlay
Gene
TRP2
MET6
IES5
TSC11

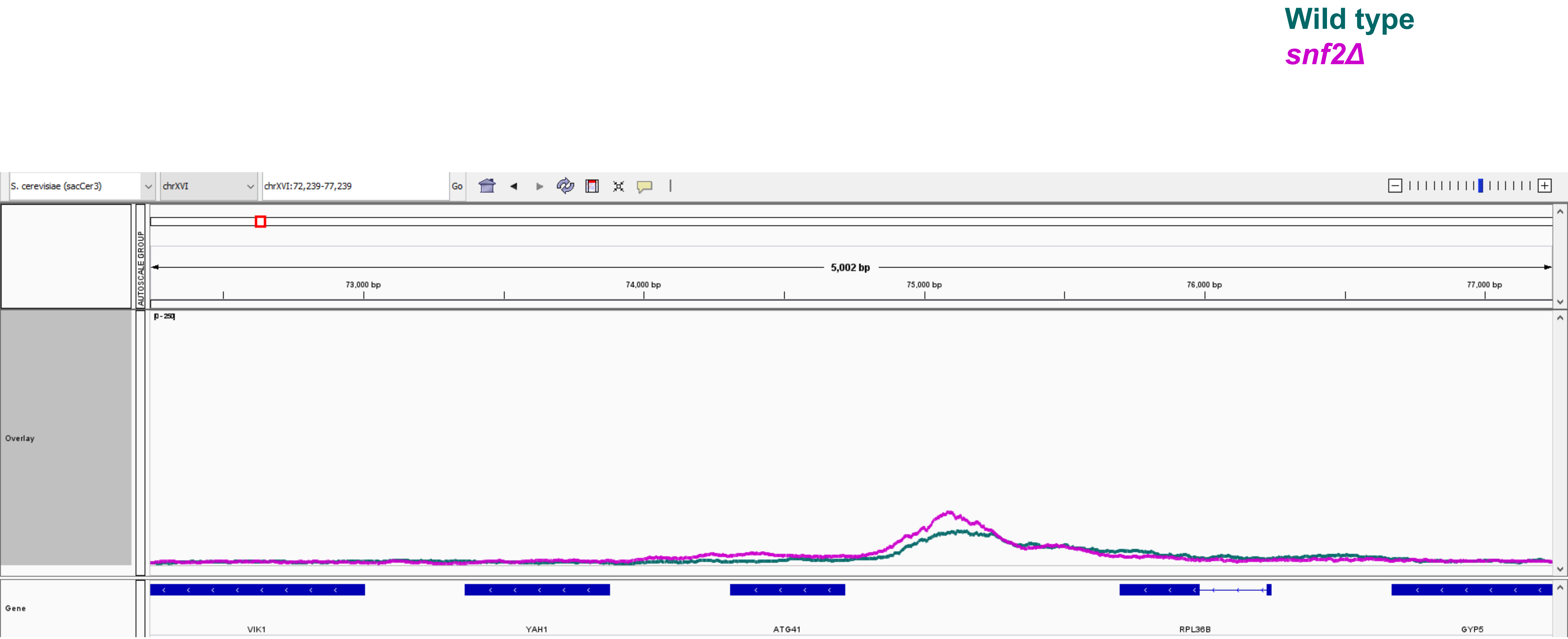
Wild type
snf2Δ
S. cerevisiae (sacCer3)
chrXVI
chrXVI:72,239-77,239
Go
AUTOSCALE GROUP
5,002 bp
73,000 bp
74,000 bp
75,000 bp
76,000 bp
77,000 bp
Overlay
Gene
VIK1
YAH1
ATG41
RPL36B
GYP5

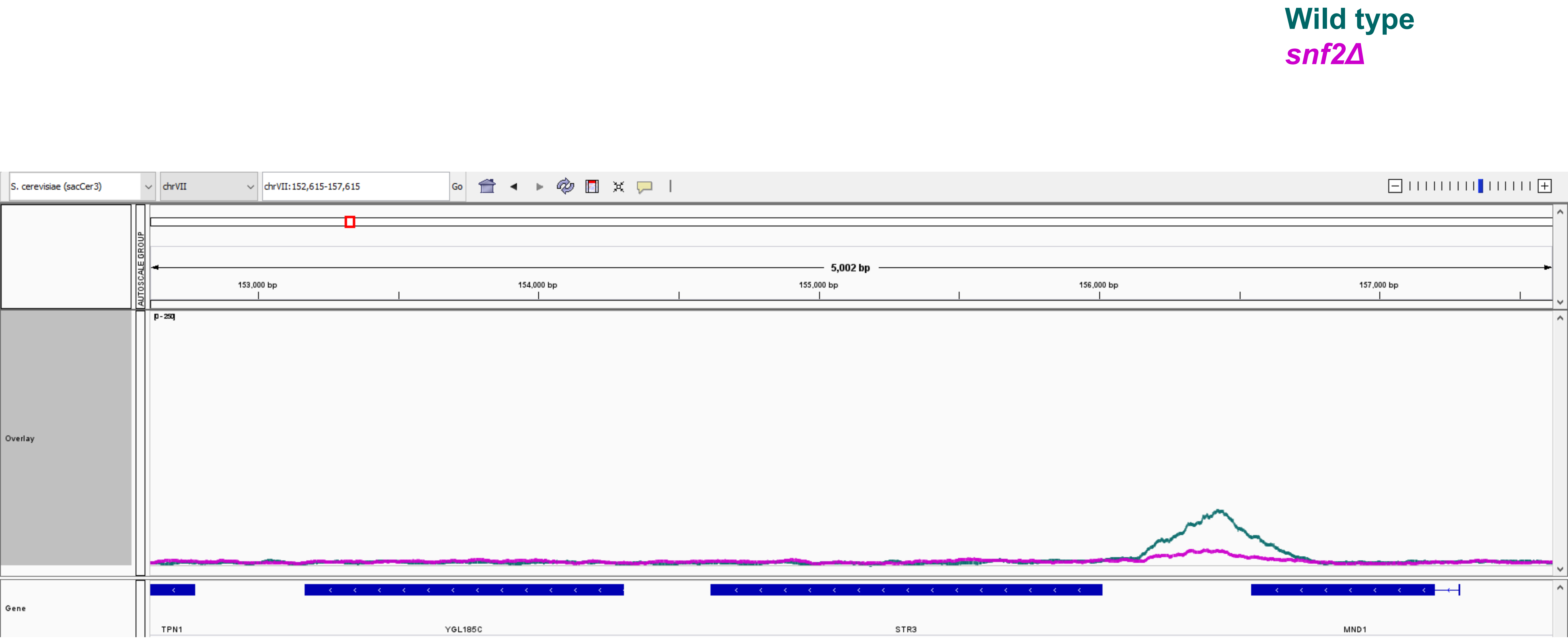

Wild type
snf2Δ
S. cerevisiae (sacCer3)
chrVII
chrVII:152,615-157,615
Go
5,002 bp
153,000 bp
154,000 bp
155,000 bp
156,000 bp
157,000 bp
AUTOSCALE GROUP
Overlay
Gene
TPN1
YGL185C
STR3
MND1

Wild type
snf2Δ

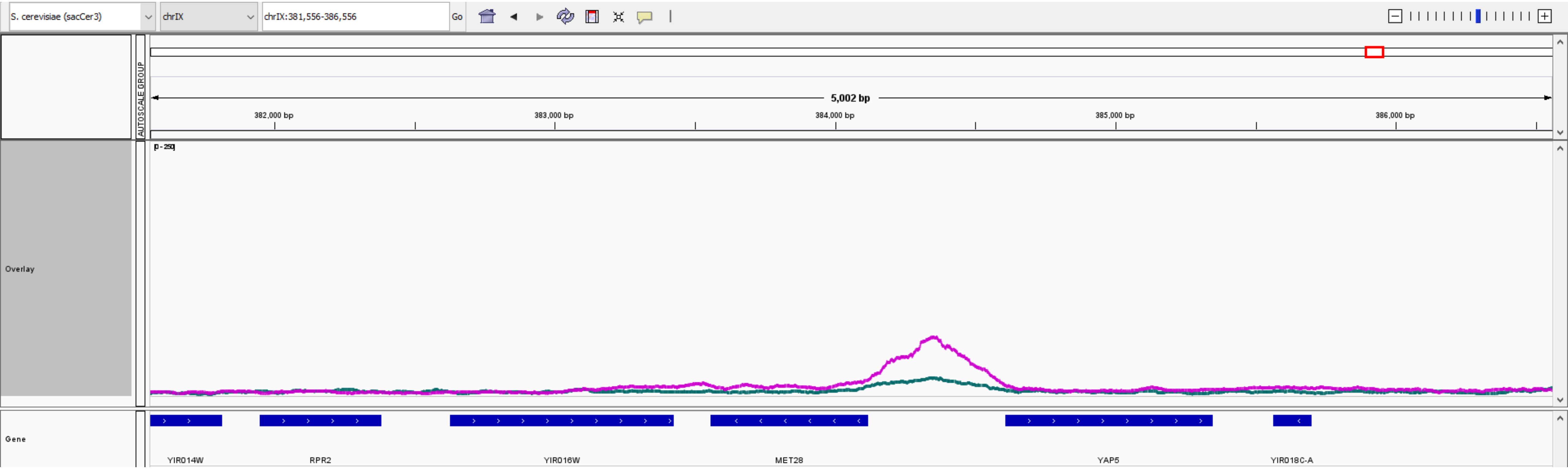

Wild type
snf2Δ

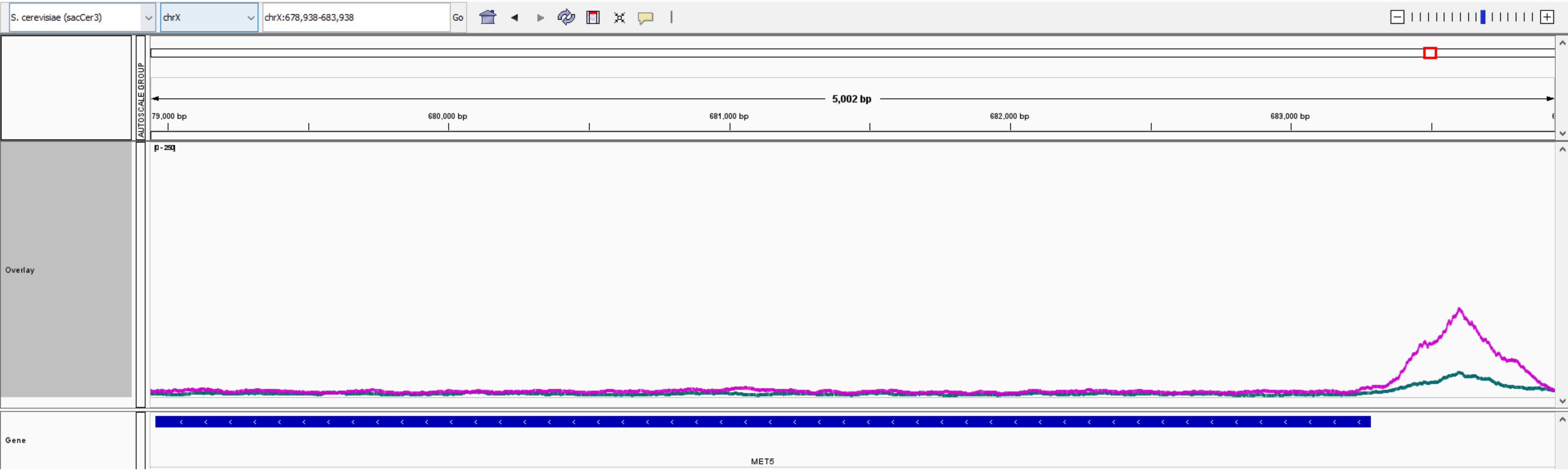

Wild type
snf2Δ

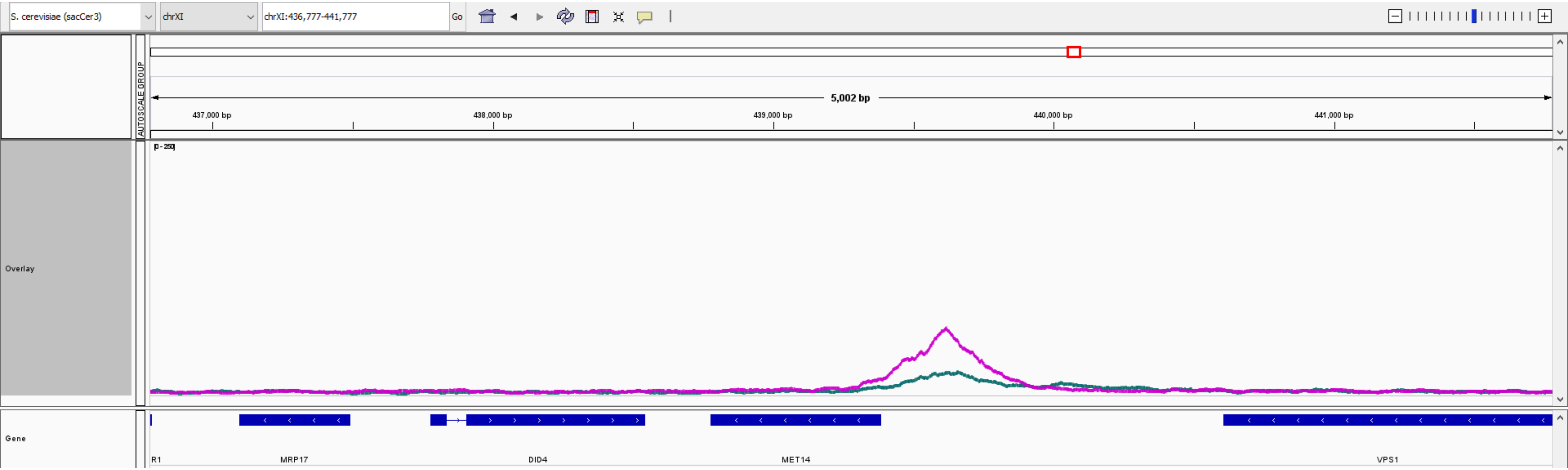

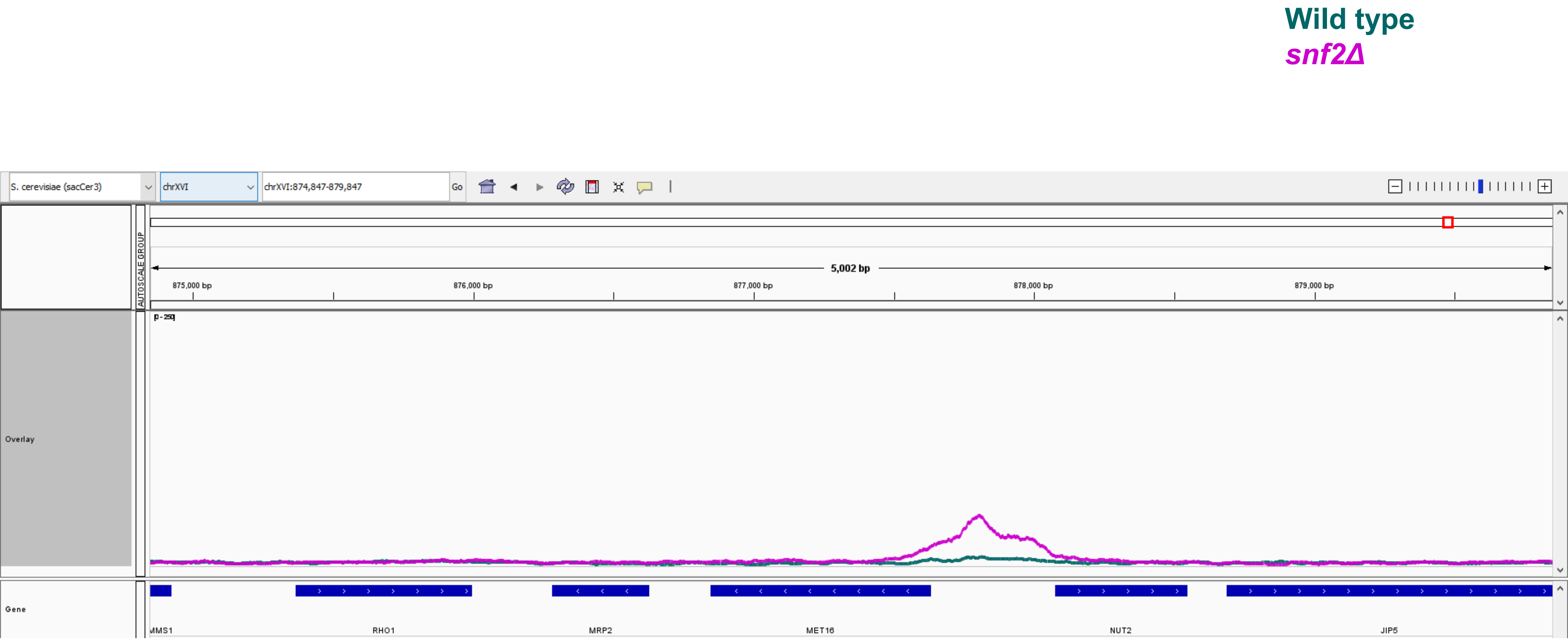
Wild type
snf2Δ
S. cerevisiae (sacCer3)
chrXVI
chrXVI:874,847-879,847
Go
5,002 bp
875,000 bp
876,000 bp
877,000 bp
878,000 bp
879,000 bp
AUTOSCALE GROUP
Overlay
Gene
MMS1
RHO1
MRP2
MET16
NUT2
JIP5

Wild type
snf2Δ

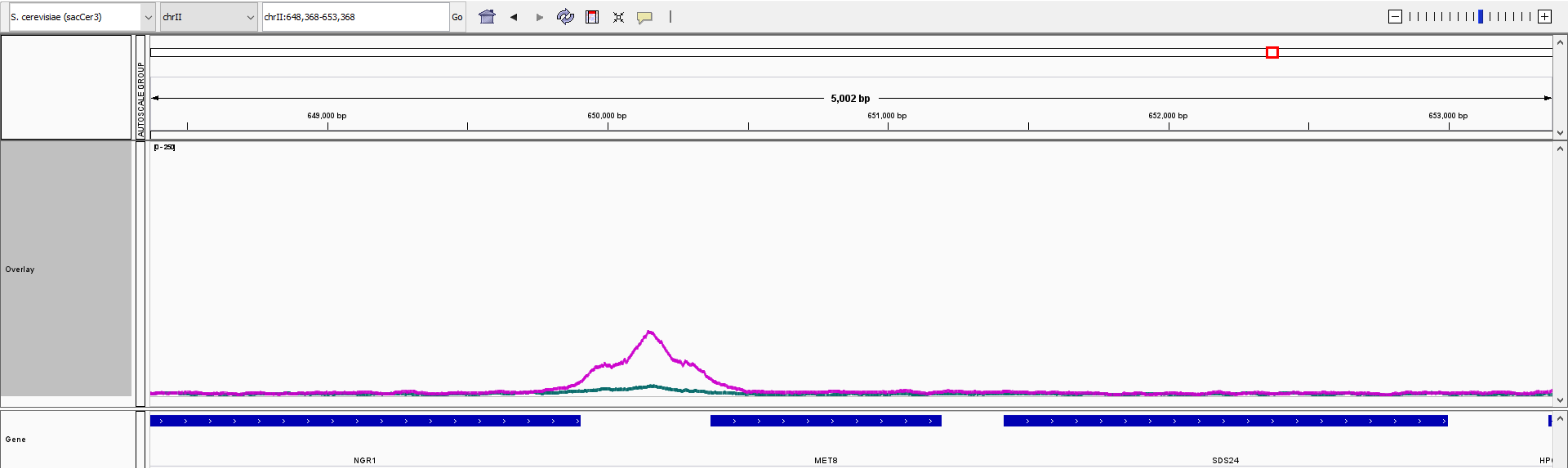

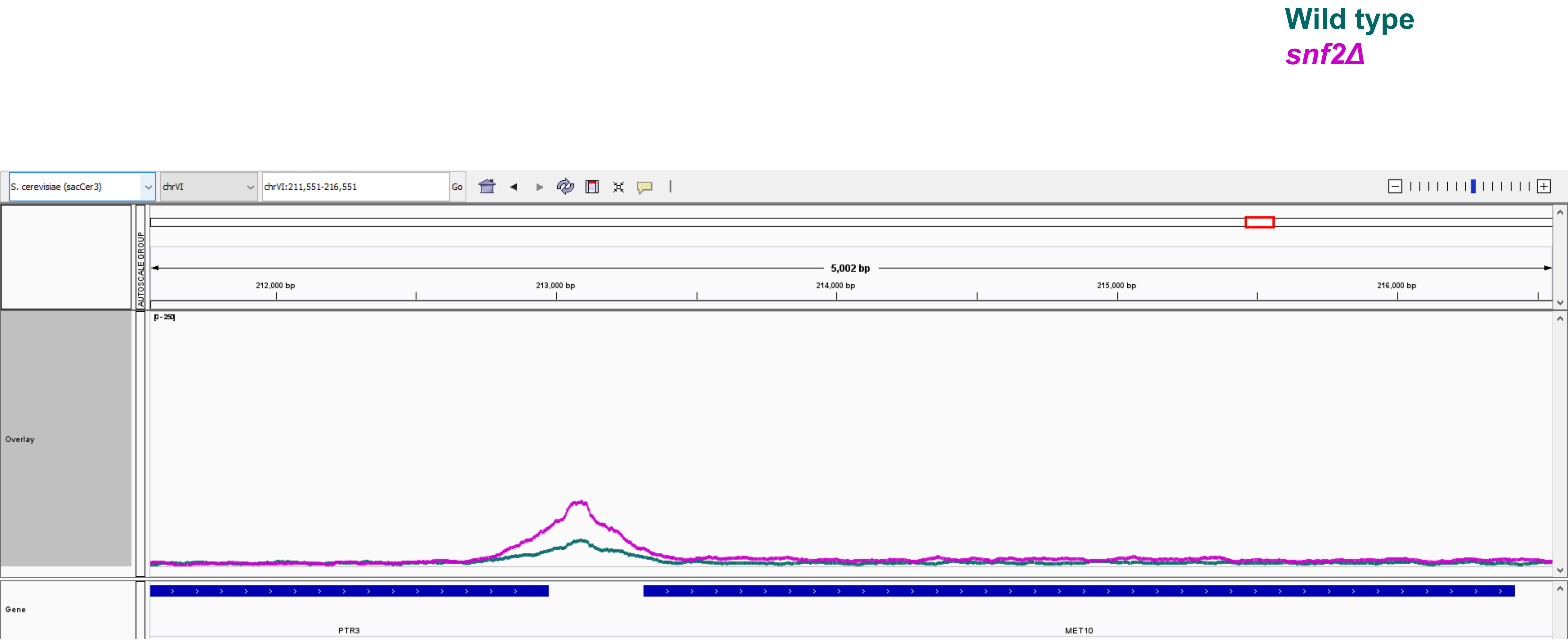
Wild type
snf2Δ
S. cerevisiae (sacCer3)
chrVI
chrVI:211,551-216,551
Go
AUTOSCALE GROUP
5,002 bp
212,000 bp
213,000 bp
214,000 bp
215,000 bp
216,000 bp
Overlay
Gene
PTR3
MET10

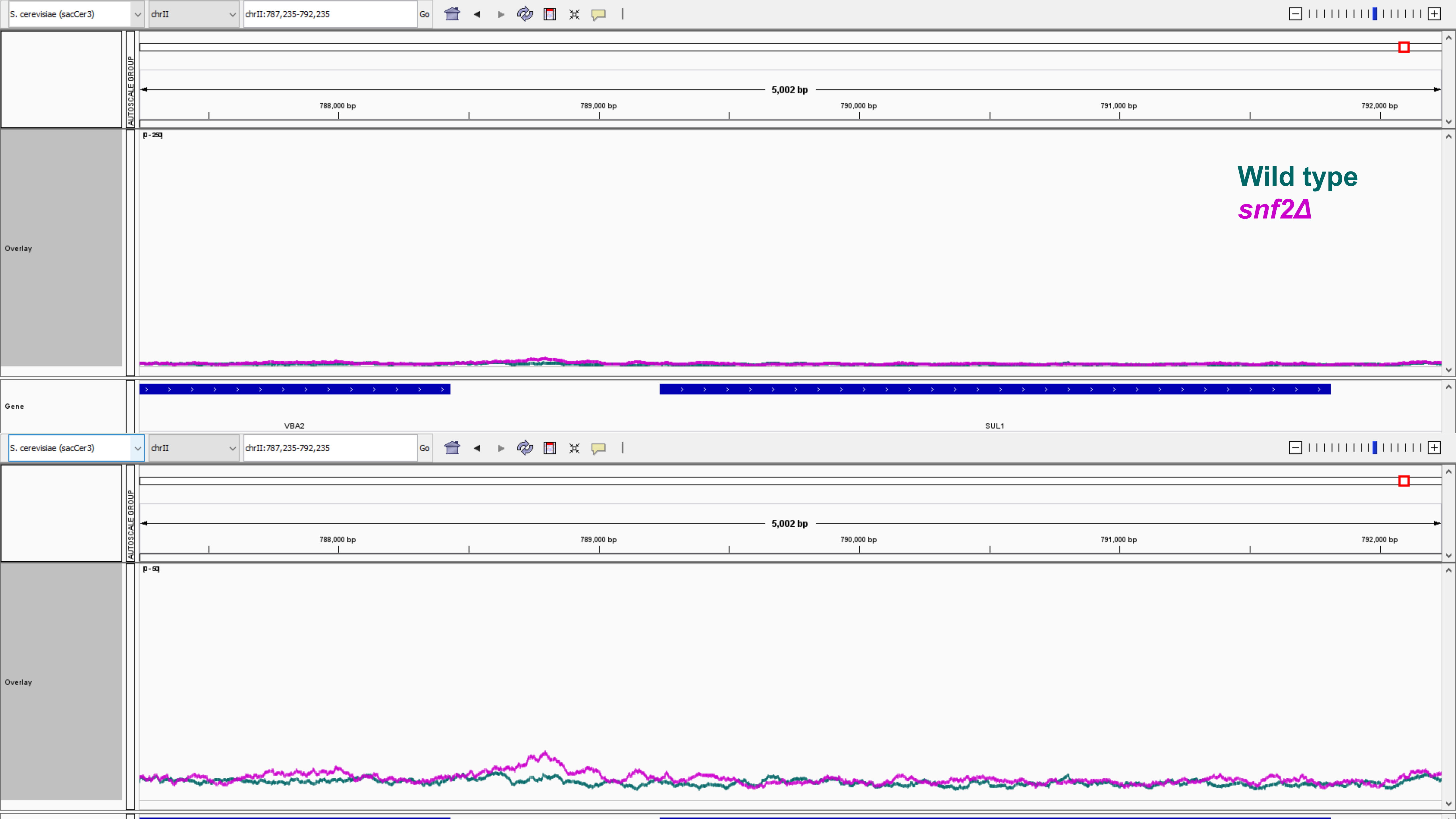
S. cerevisiae (sacCer3)
chrII
chrII:787,235-792,235
Go
AUTOSCALE GROUP
5,002 bp
788,000 bp
789,000 bp
790,000 bp
791,000 bp
792,000 bp
Overlay
Wild type
snf2Δ
Gene
VBA2
SUL1
S. cerevisiae (sacCer3)
chrII
chrII:787,235-792,235
Go
AUTOSCALE GROUP
5,002 bp
788,000 bp
789,000 bp
790,000 bp
791,000 bp
792,000 bp
Overlay

Wild type
snf2Δ

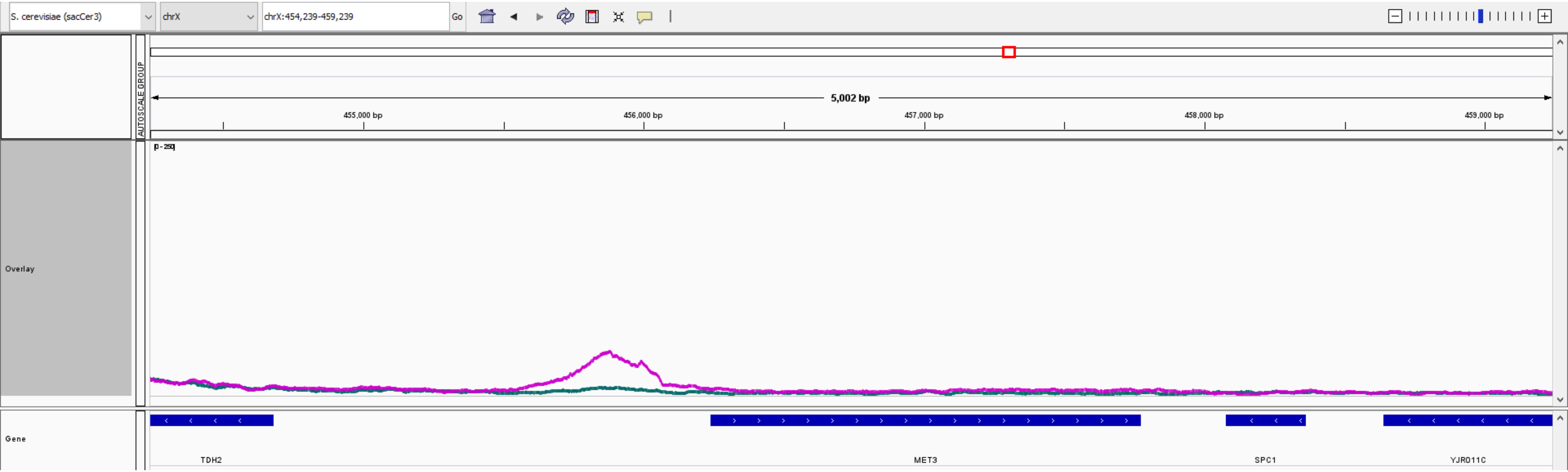

Wild type
snf2Δ

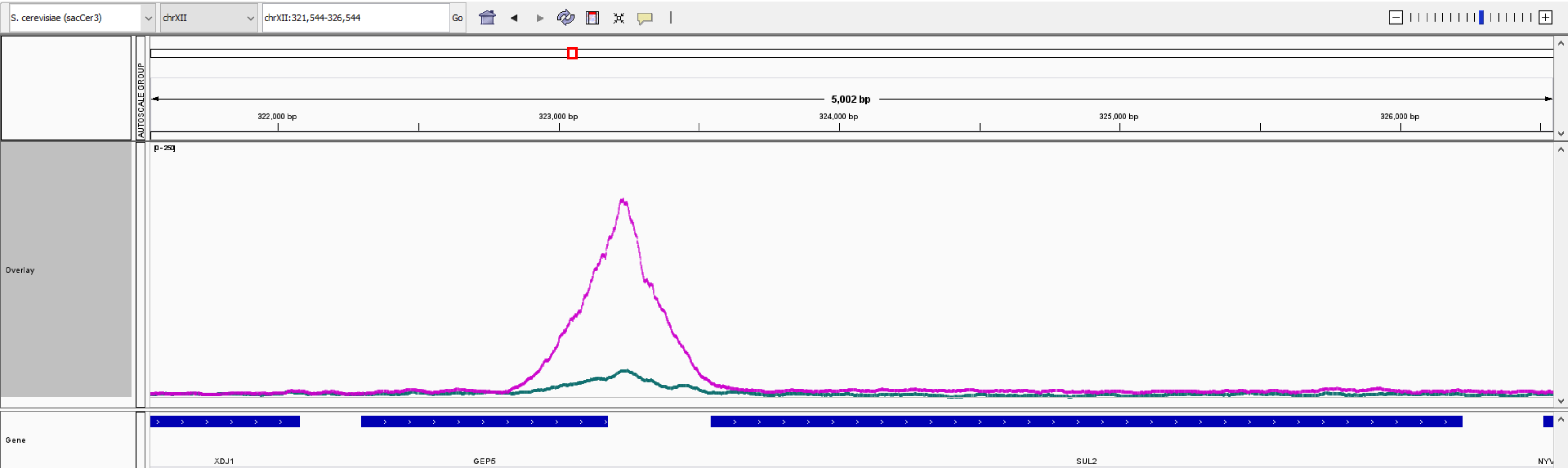

Wild type
snf2Δ

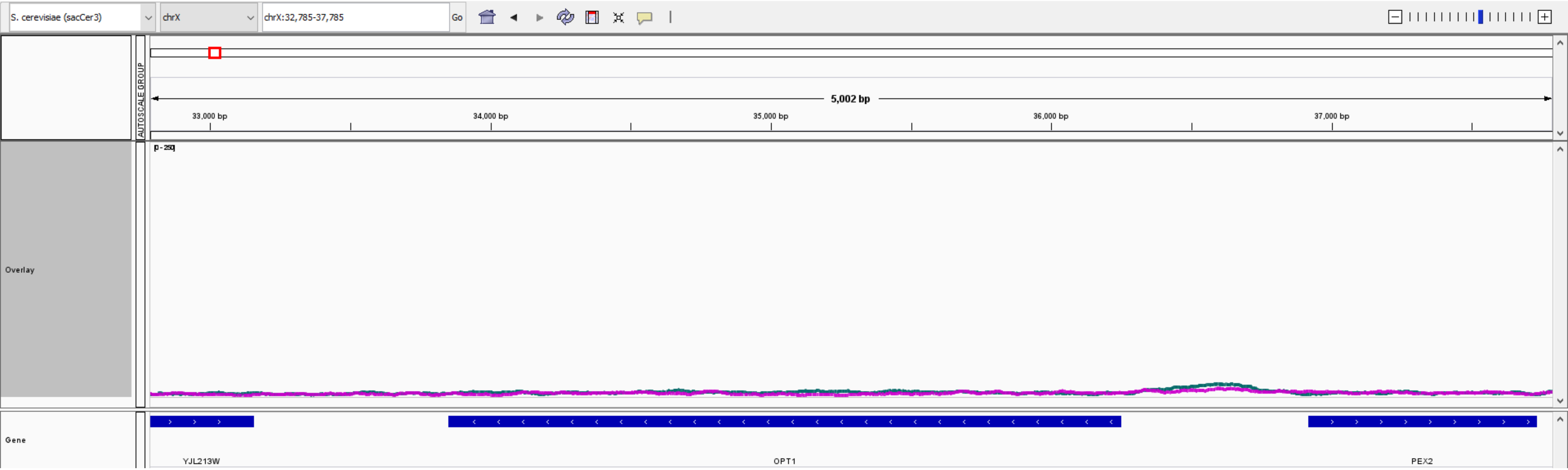

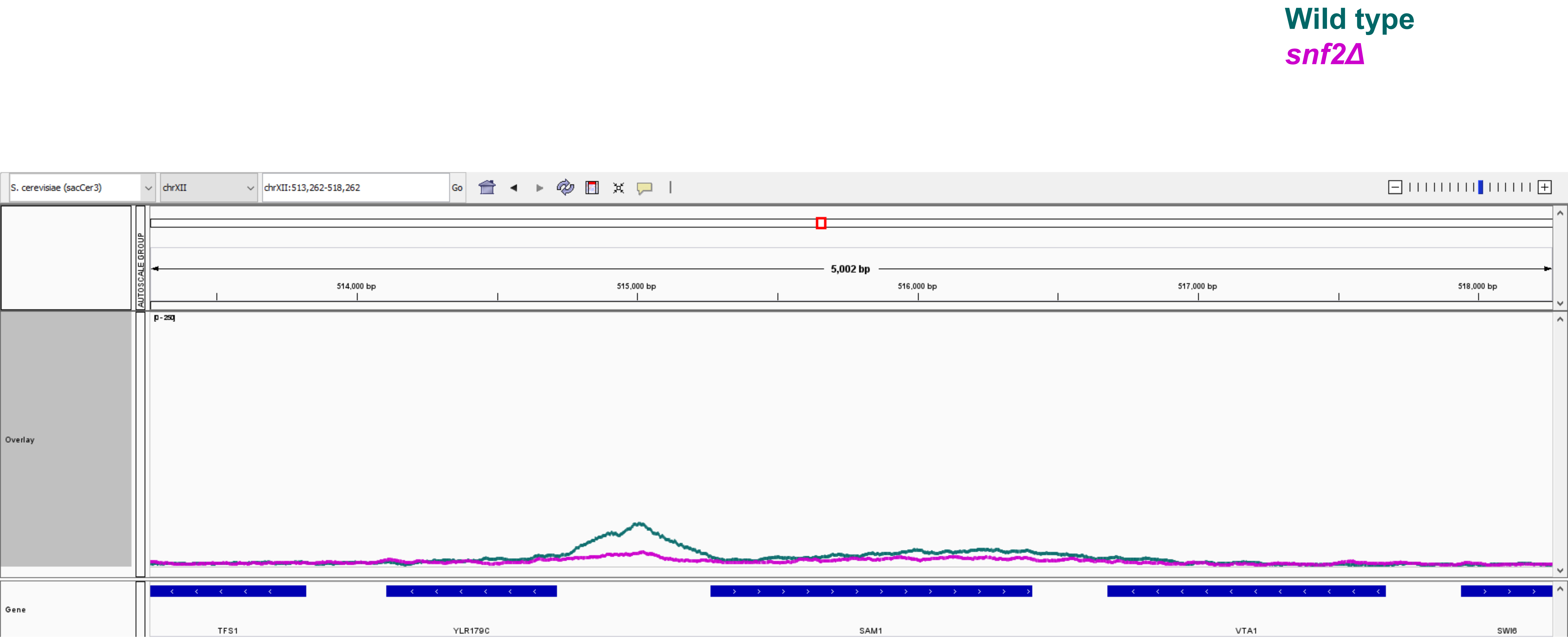

Wild type
snf2Δ
S. cerevisiae (sacCer3)
chrXII
chrXII:513,262-518,262
Go
5,002 bp
514,000 bp
515,000 bp
516,000 bp
517,000 bp
518,000 bp
AUTOSCALE GROUP
Overlay
Gene
TFS1
YLR179C
SAM1
VTA1
SWI6

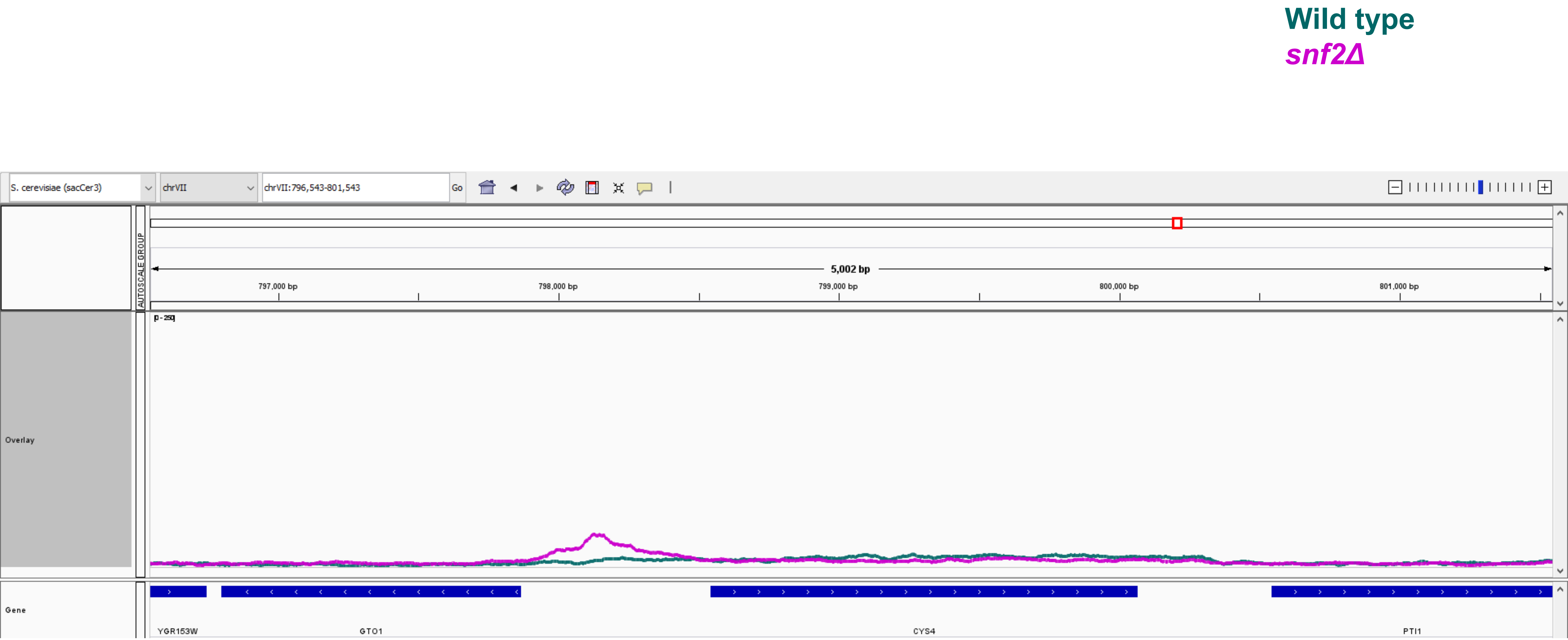

Wild type
snf2Δ
S. cerevisiae (sacCer3)
chrVII
chrVII:796,543-801,543
Go
5,002 bp
797,000 bp
798,000 bp
799,000 bp
800,000 bp
801,000 bp
AUTOSCALE GROUP
Overlay
Gene
YGR153W
GTO1
CYS4
PTI1

Wild type
snf2Δ

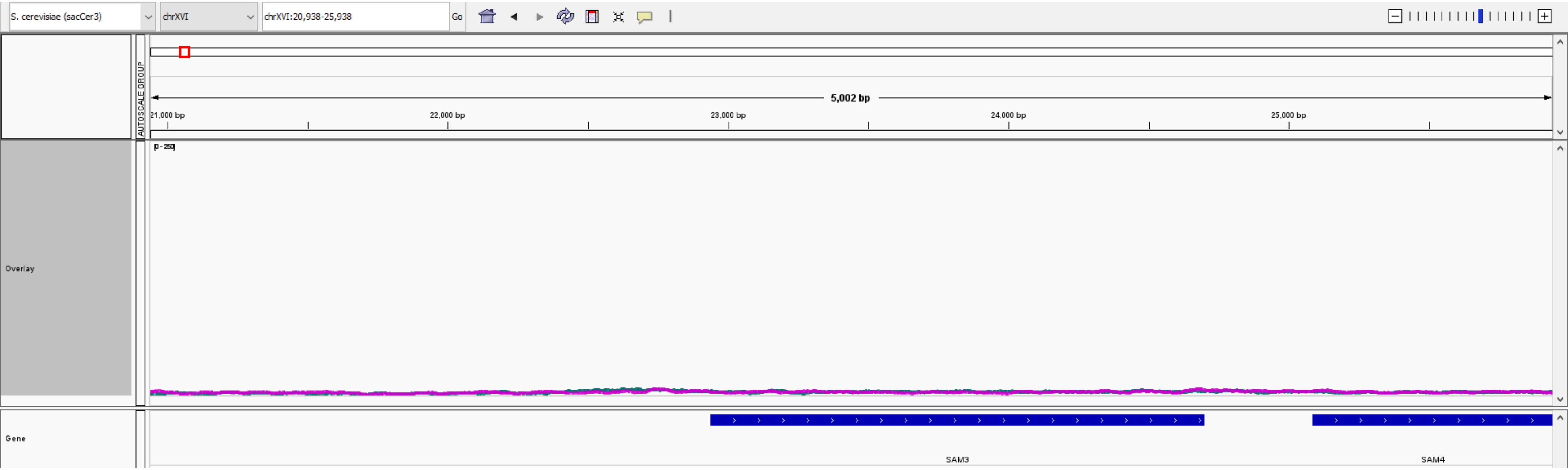

Wild type
snf2Δ

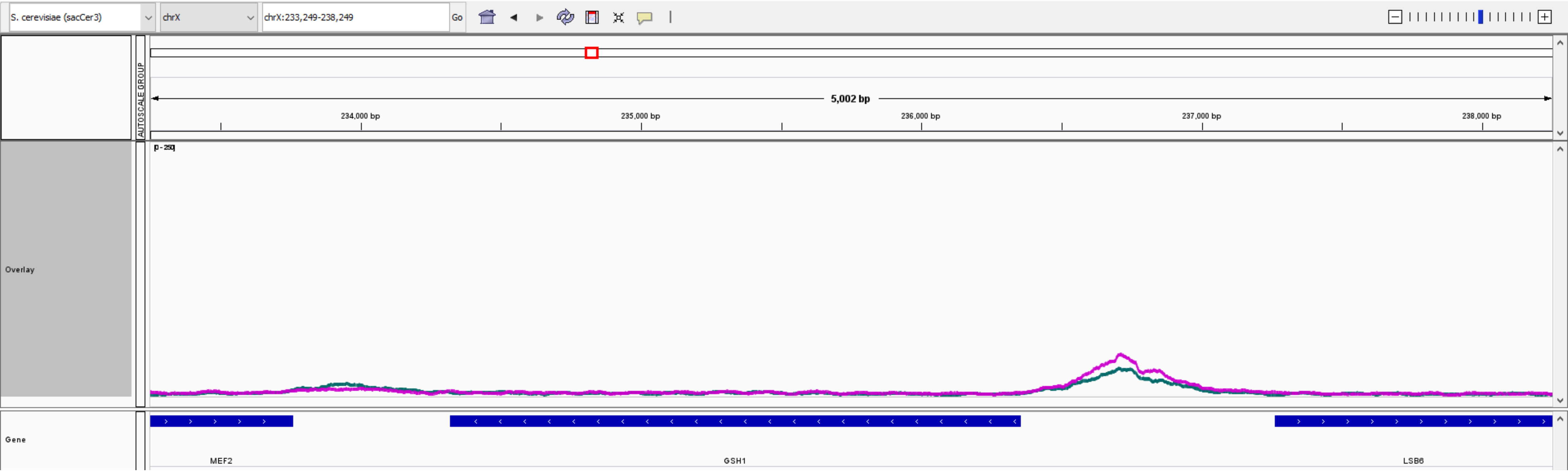

Wild type
snf2Δ

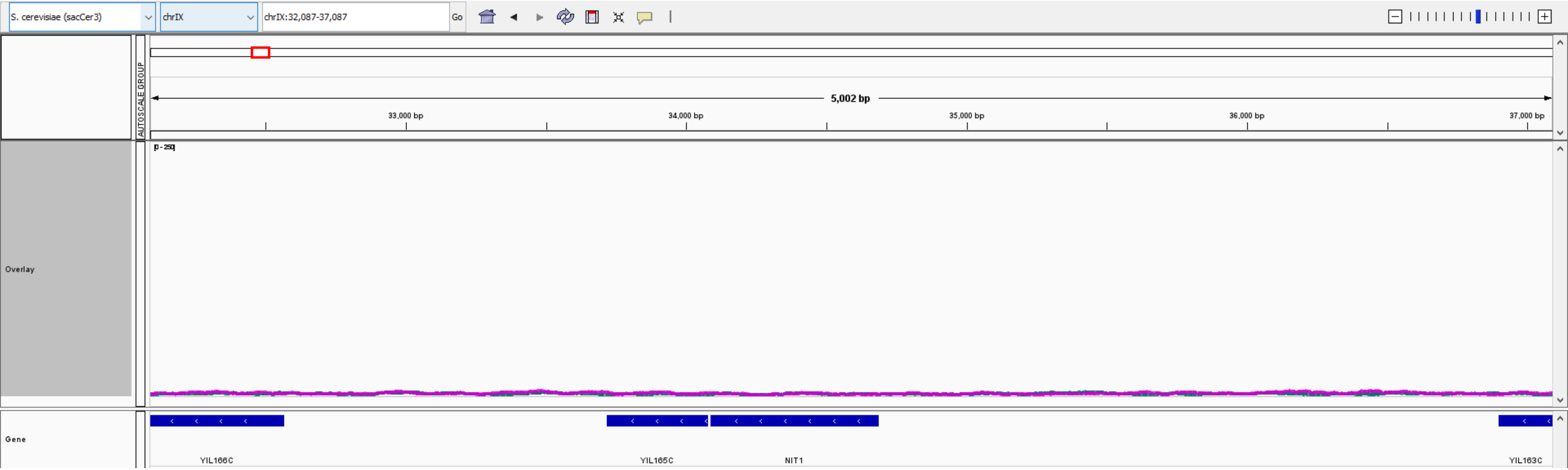

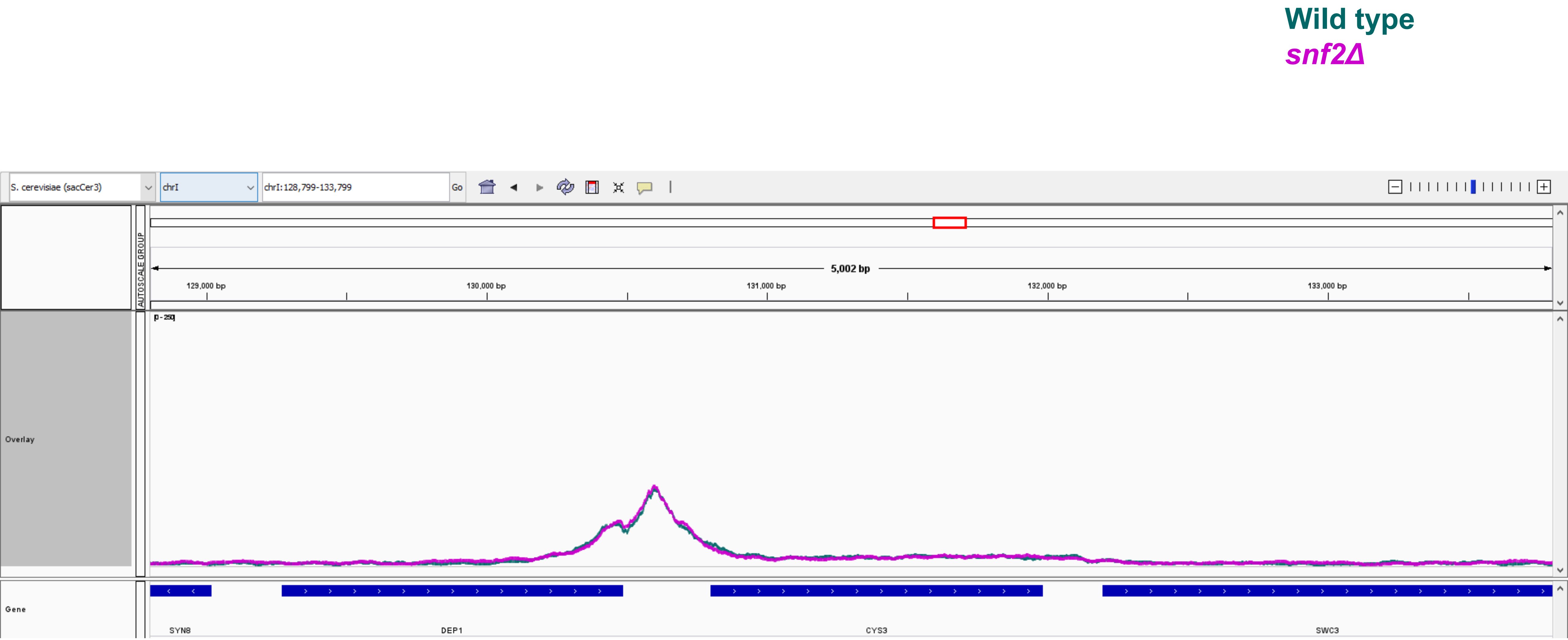
Wild type
snf2Δ
S. cerevisiae (sacCer3)
chrI
chrI:128,799-133,799
Go
5,002 bp
129,000 bp
130,000 bp
131,000 bp
132,000 bp
133,000 bp
AUTOSCALE GROUP
Overlay
Gene
SYN8
DEP1
CYS3
SWC3

Wild type
snf2Δ

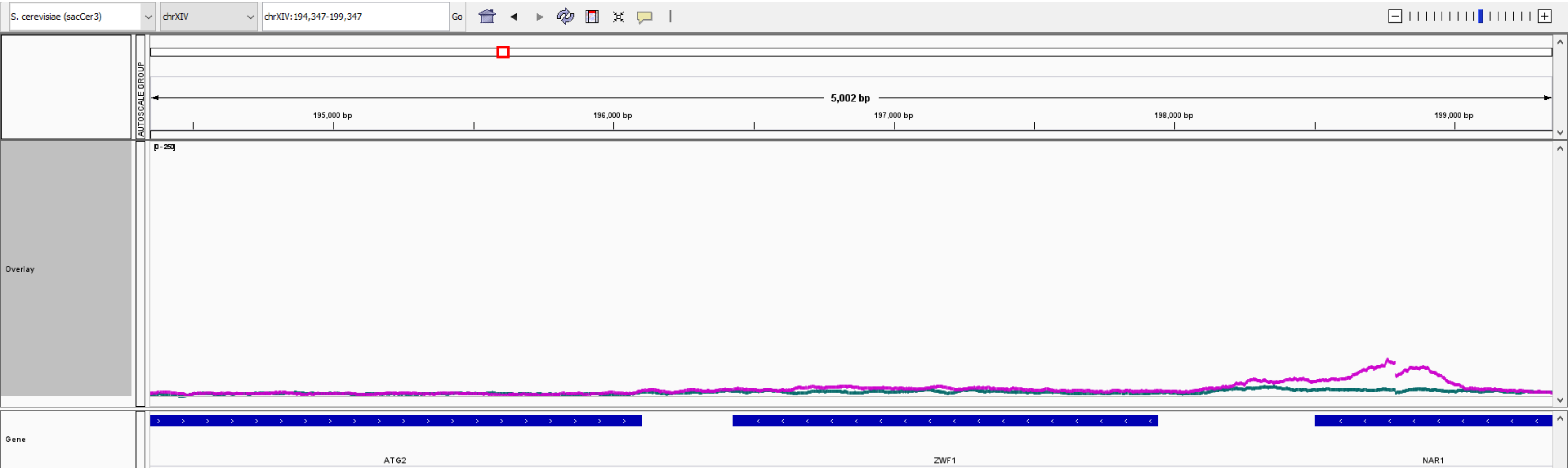

Wild type
snf2Δ

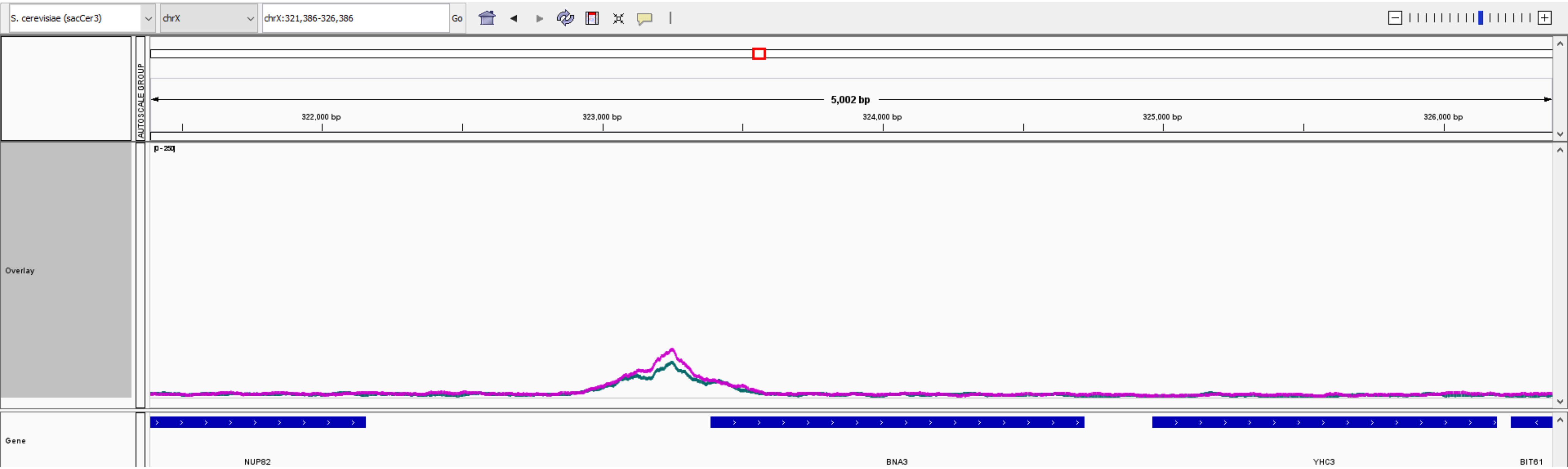

Wild type
snf2Δ

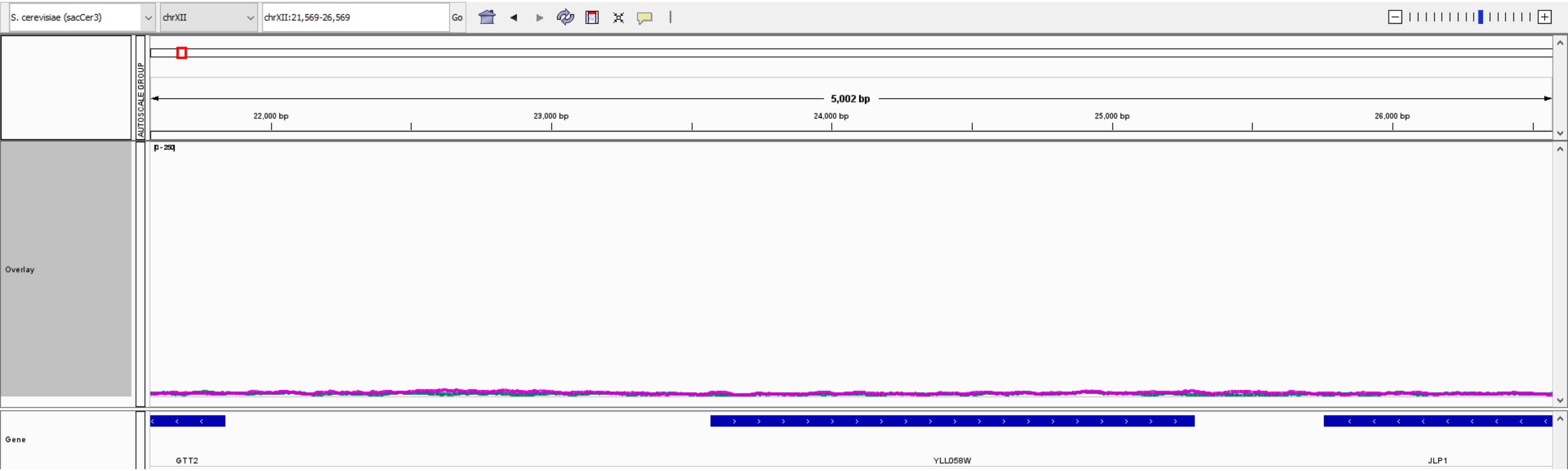

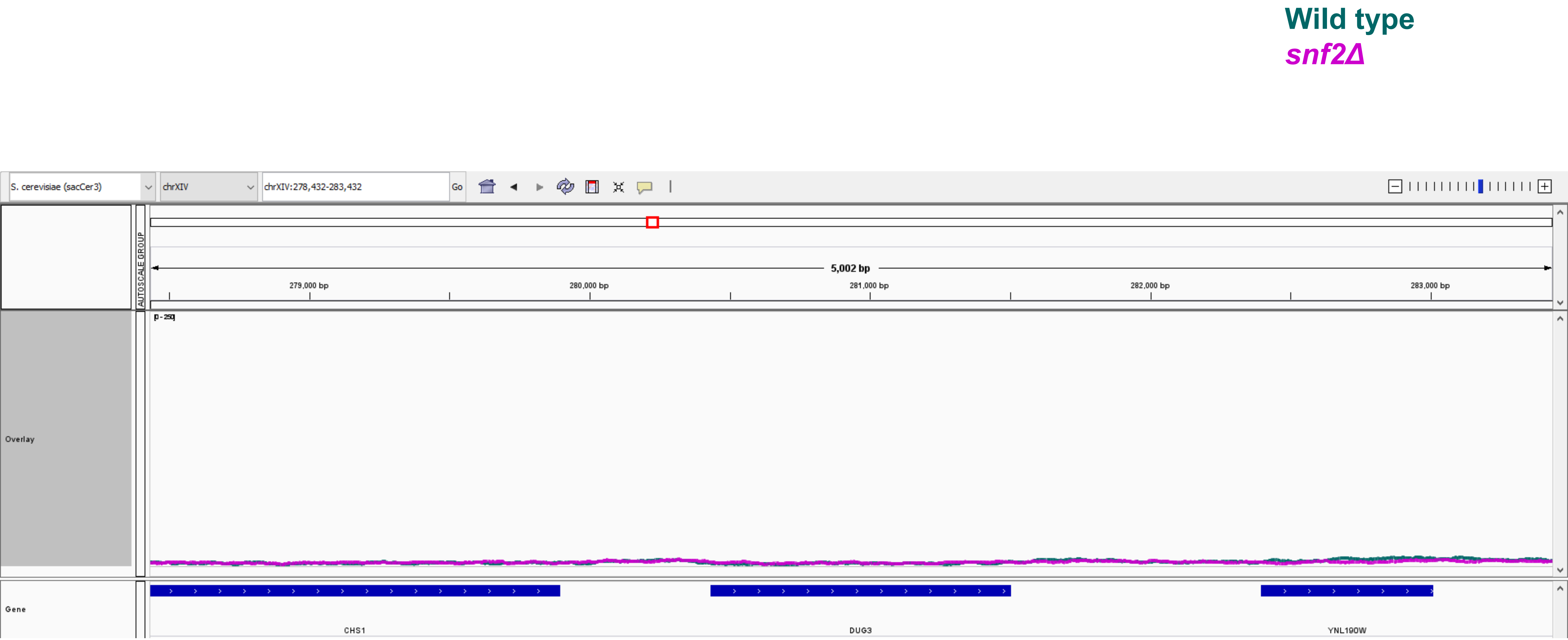
Wild type
snf2Δ
S. cerevisiae (sacCer3)
chrXIV
chrXIV:278,432-283,432
Go
AUTOSCALE GROUP
5,002 bp
279,000 bp
280,000 bp
281,000 bp
282,000 bp
283,000 bp
Overlay
Gene
CHS1
DUG3
YNL190W

Wild type
snf2Δ

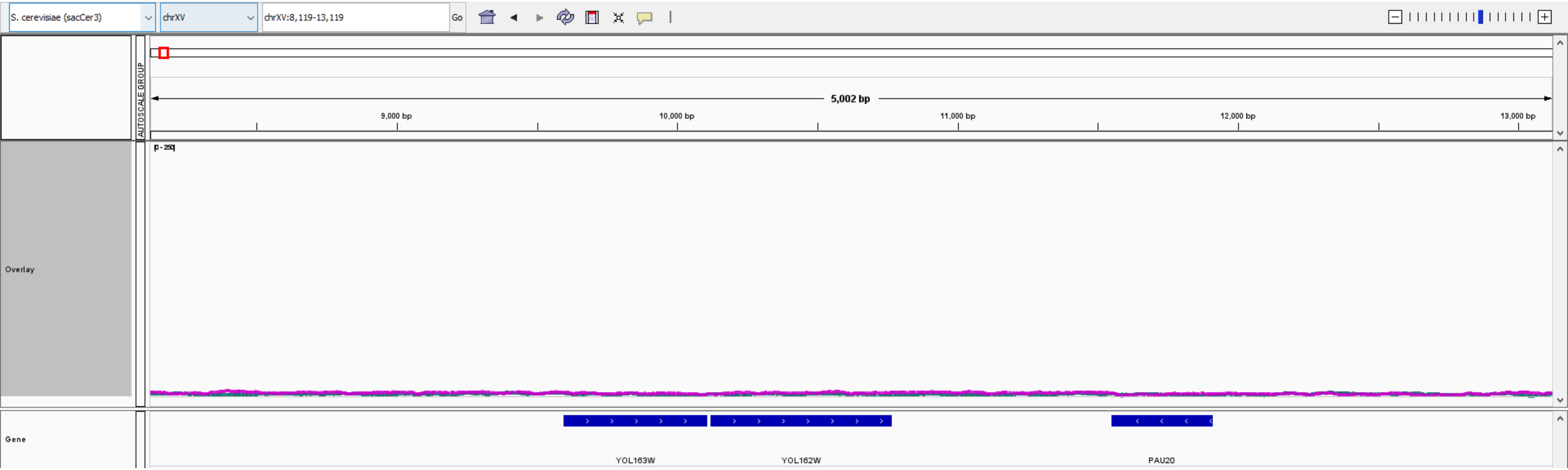

Wild type
snf2Δ

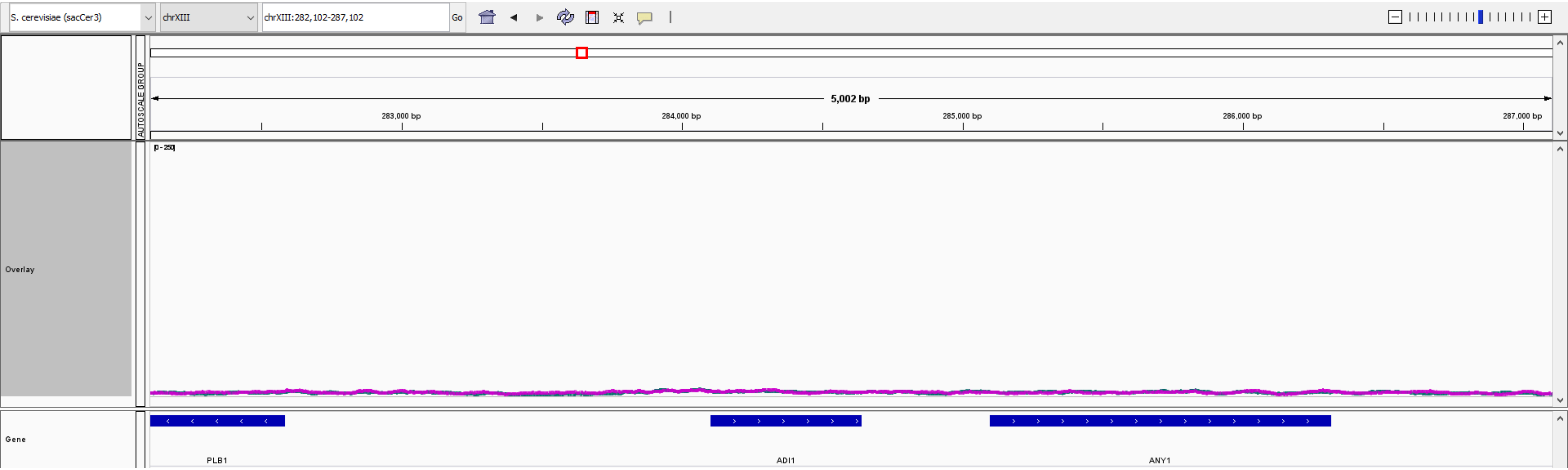

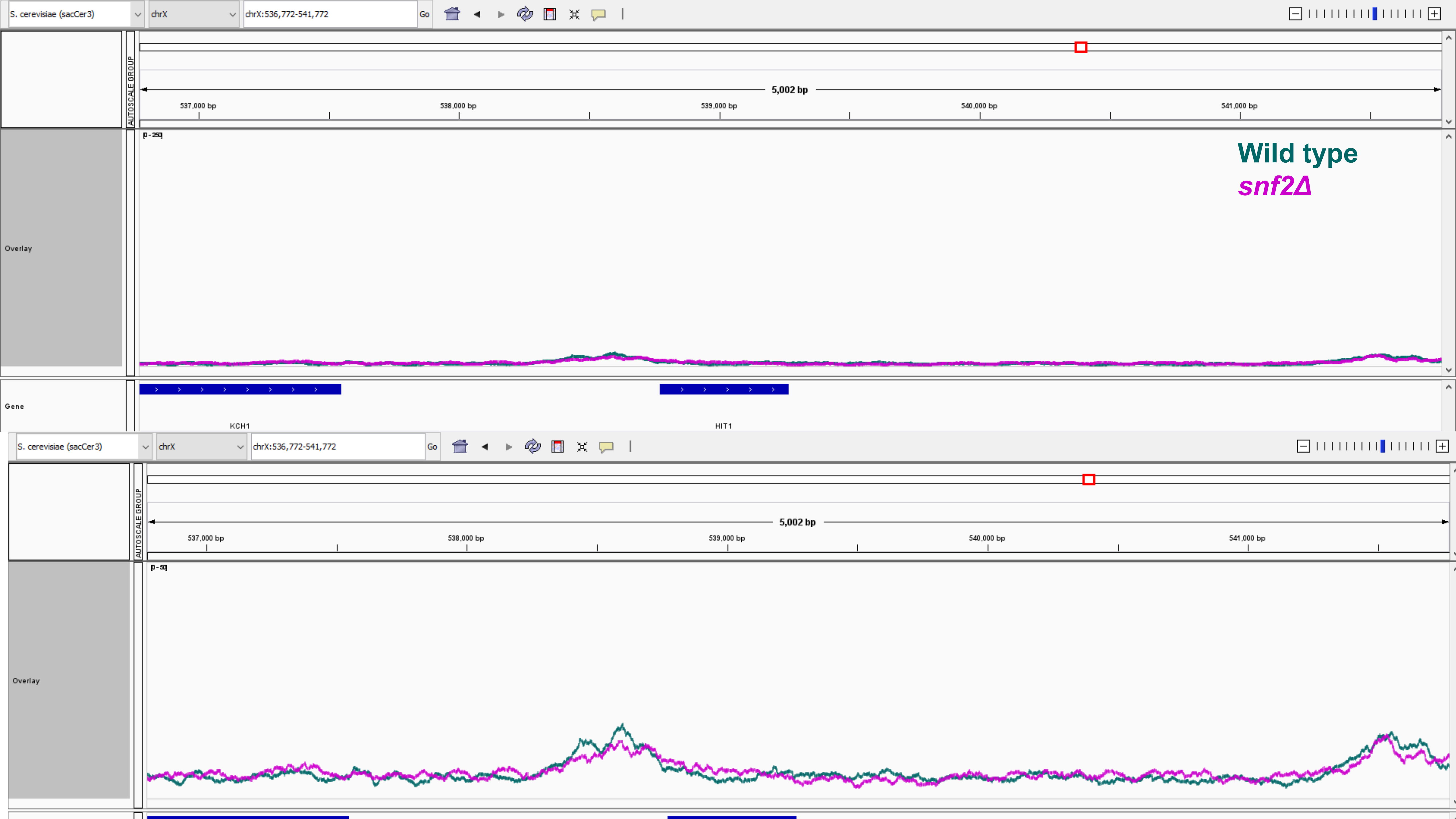

S. cerevisiae (sacCer3)
chrX
chrX:536,772-541,772
Go
AUTOSCALE GROUP
5,002 bp
537,000 bp
538,000 bp
539,000 bp
540,000 bp
541,000 bp
[0 - 250]
Wild type
snf2Δ
Overlay
Gene
KCH1
HIT1
S. cerevisiae (sacCer3)
chrX
chrX:536,772-541,772
Go
AUTOSCALE GROUP
5,002 bp
537,000 bp
538,000 bp
539,000 bp
540,000 bp
541,000 bp
[0 - 50]
Overlay

Wild type
snf2Δ

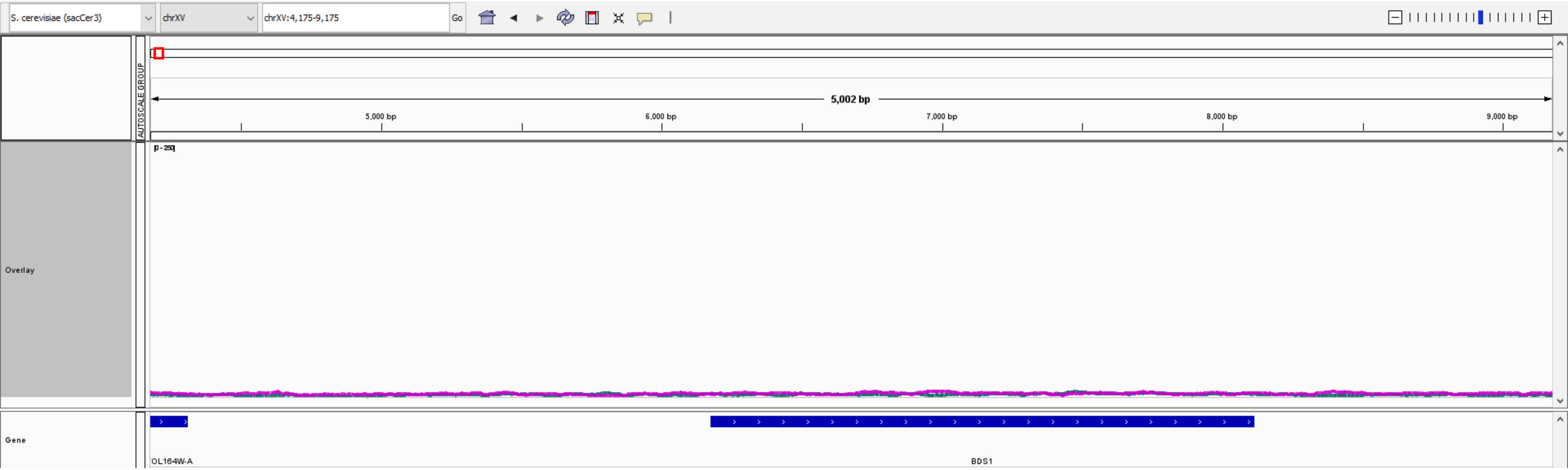

Wild type
snf2Δ

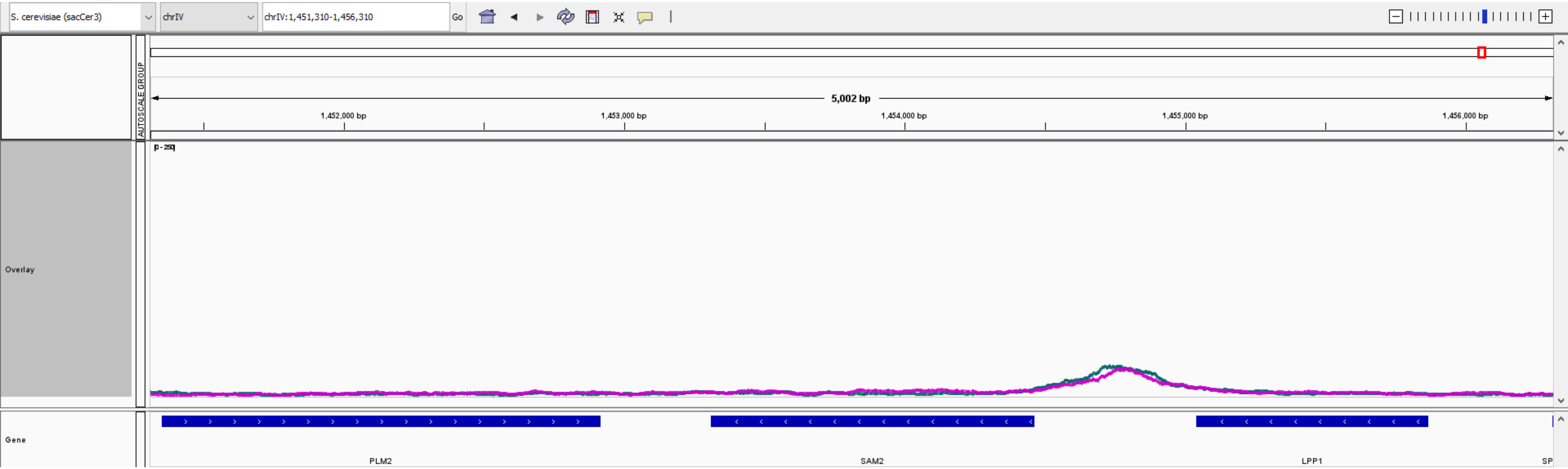

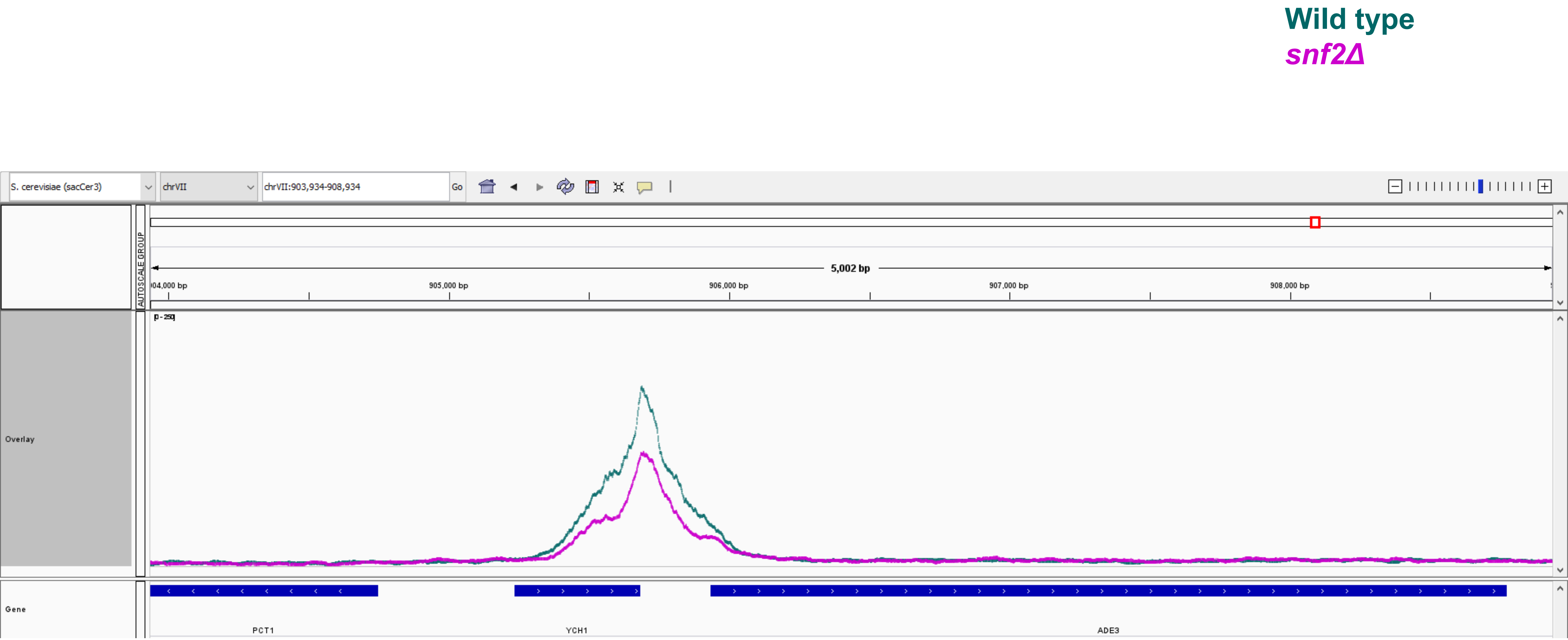

Wild type
snf2Δ
S. cerevisiae (sacCer3)
chrVII
chrVII:903,934-908,934
Go
AUTOSCALE GROUP
5,002 bp
904,000 bp
905,000 bp
906,000 bp
907,000 bp
908,000 bp
Overlay
Gene
PCT1
YCH1
ADE3

Wild type
snf2Δ

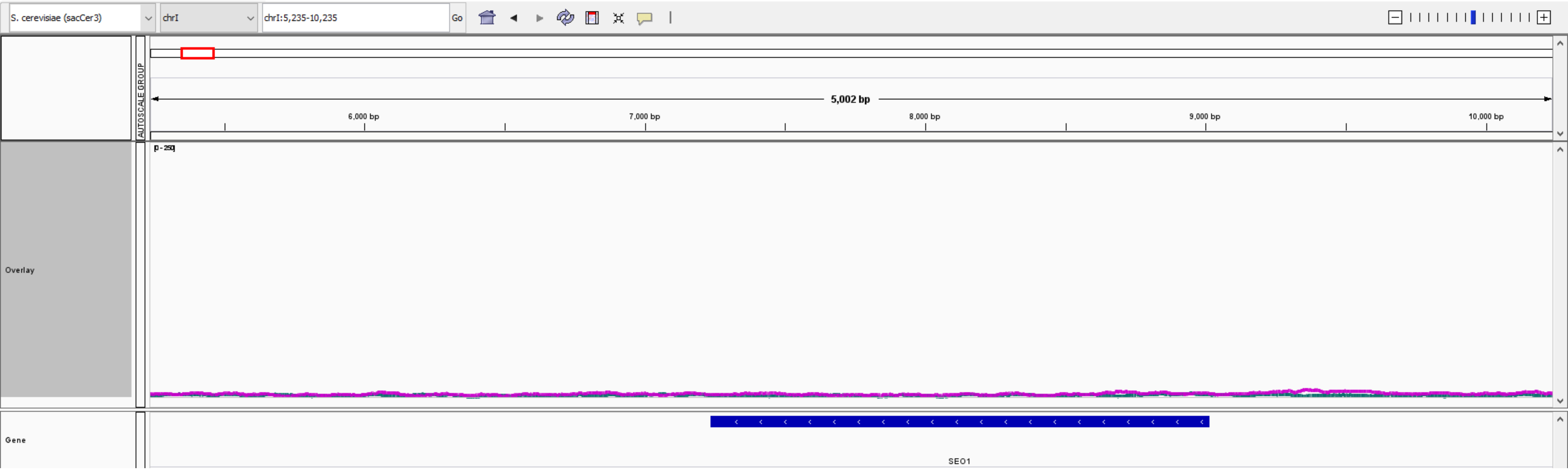

Wild type
snf2Δ

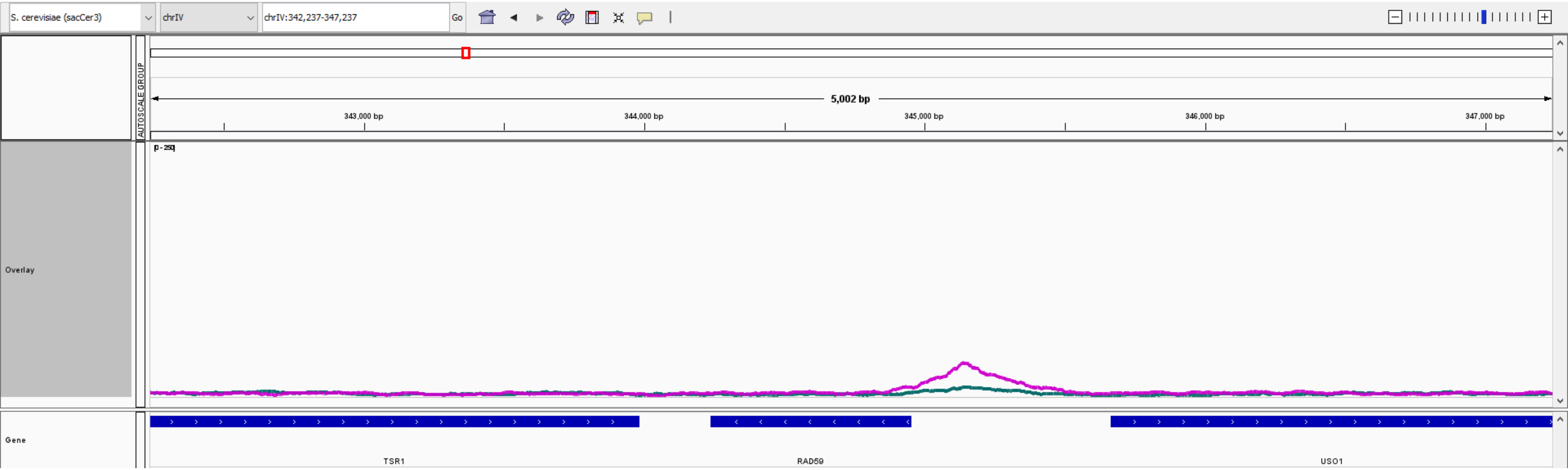

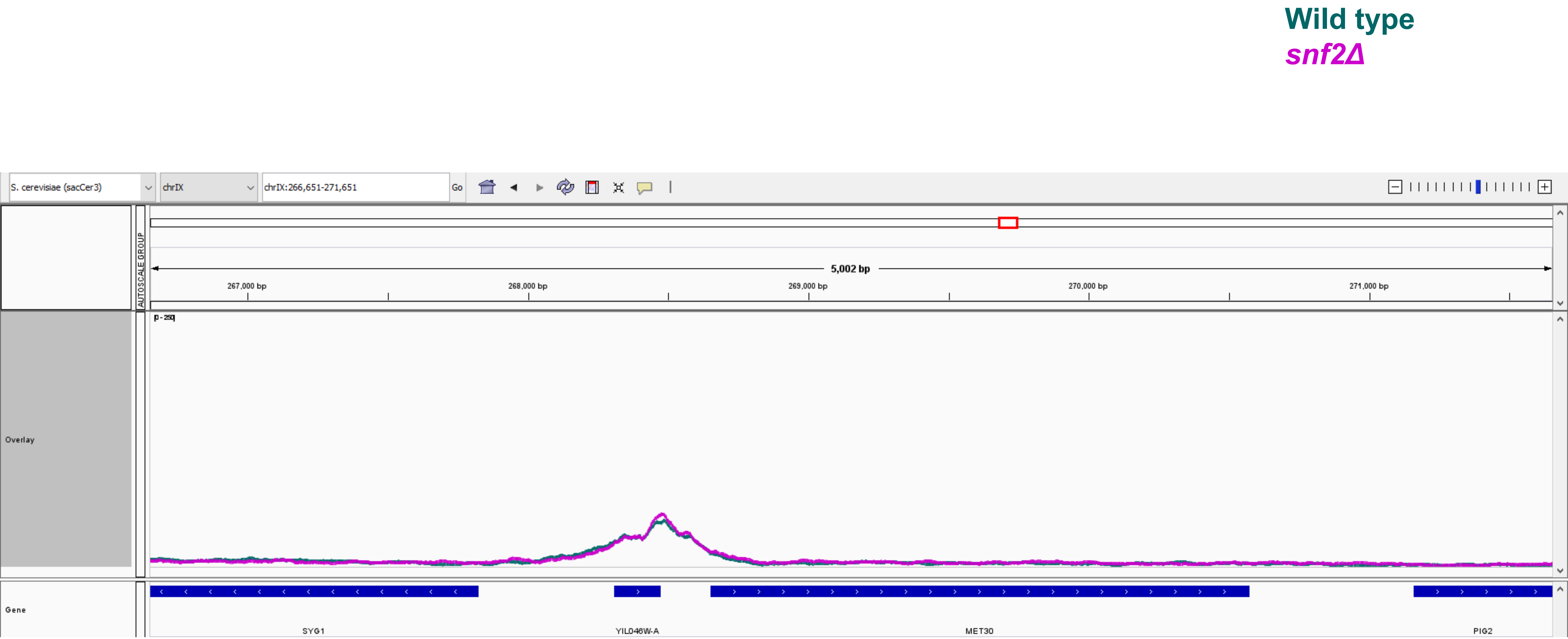
Wild type
snf2Δ
S. cerevisiae (sacCer3)
chrIX
chrIX:266,651-271,651
Go
AUTOSCALE GROUP
5,002 bp
267,000 bp
268,000 bp
269,000 bp
270,000 bp
271,000 bp
Overlay
Gene
SYG1
YIL046W-A
MET30
PIG2

Wild type
snf2Δ

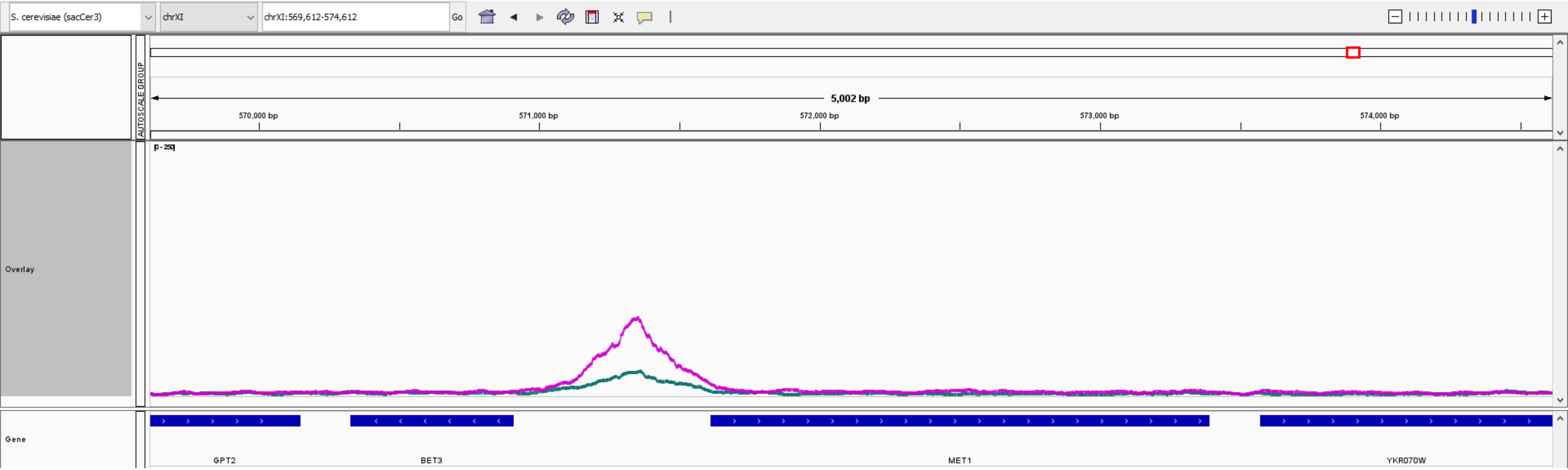

Wild type
snf2Δ

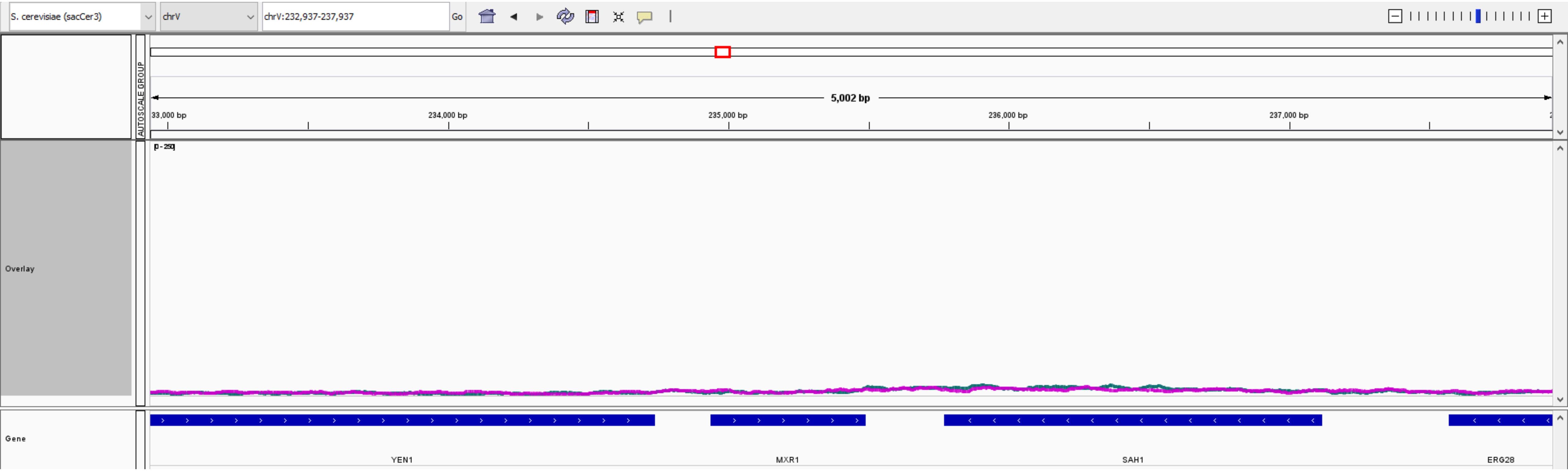

Wild type
snf2Δ

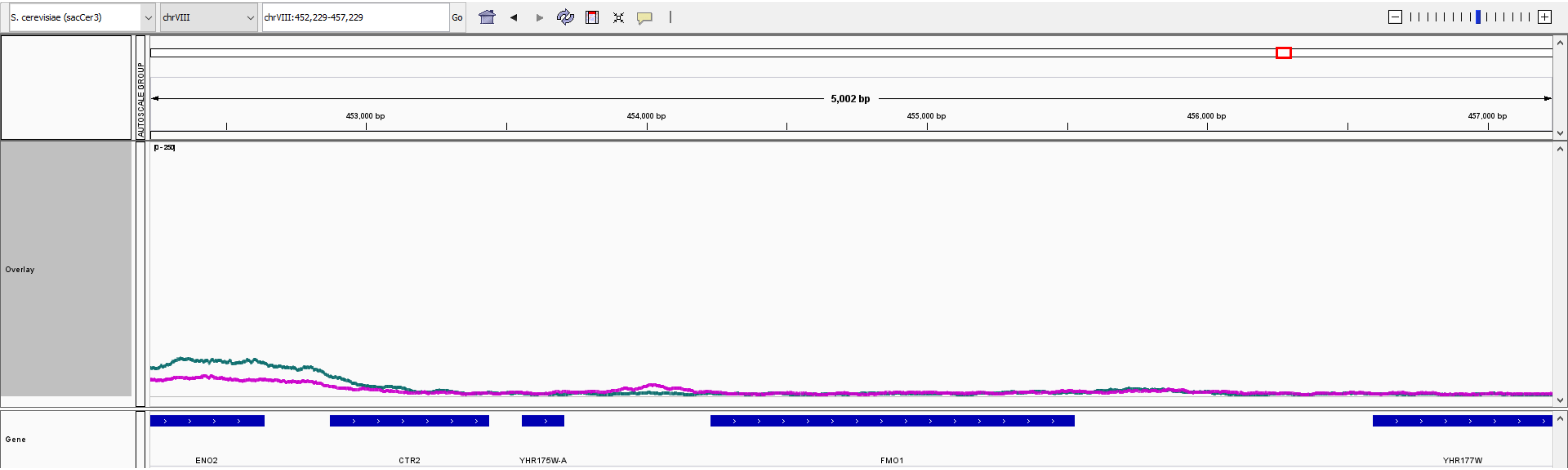

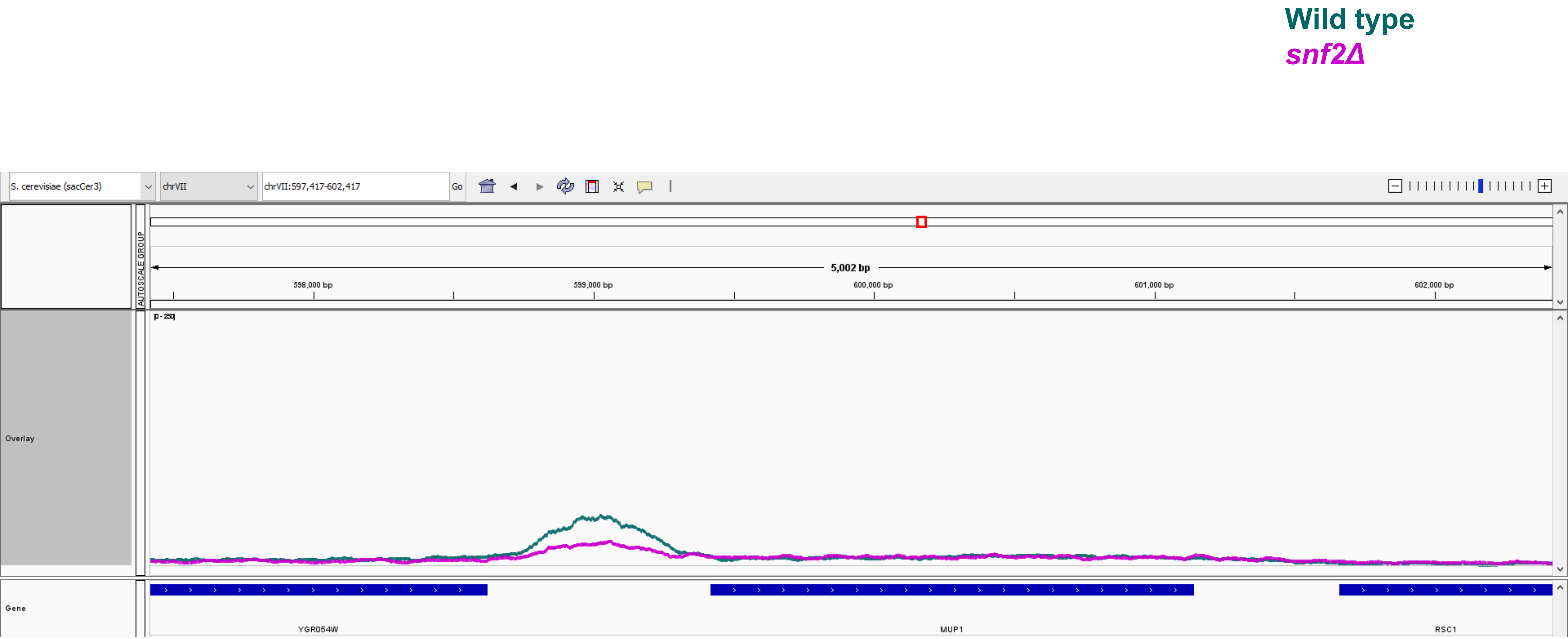

Wild type
snf2Δ
S. cerevisiae (sacCer3)
chrVII
chrVII:597,417-602,417
Go
AUTOSCALE GROUP
5,002 bp
598,000 bp
599,000 bp
600,000 bp
601,000 bp
602,000 bp
[0 - 250]
Overlay
Gene
YGR054W
MUP1
RSC1

Wild type
snf2Δ

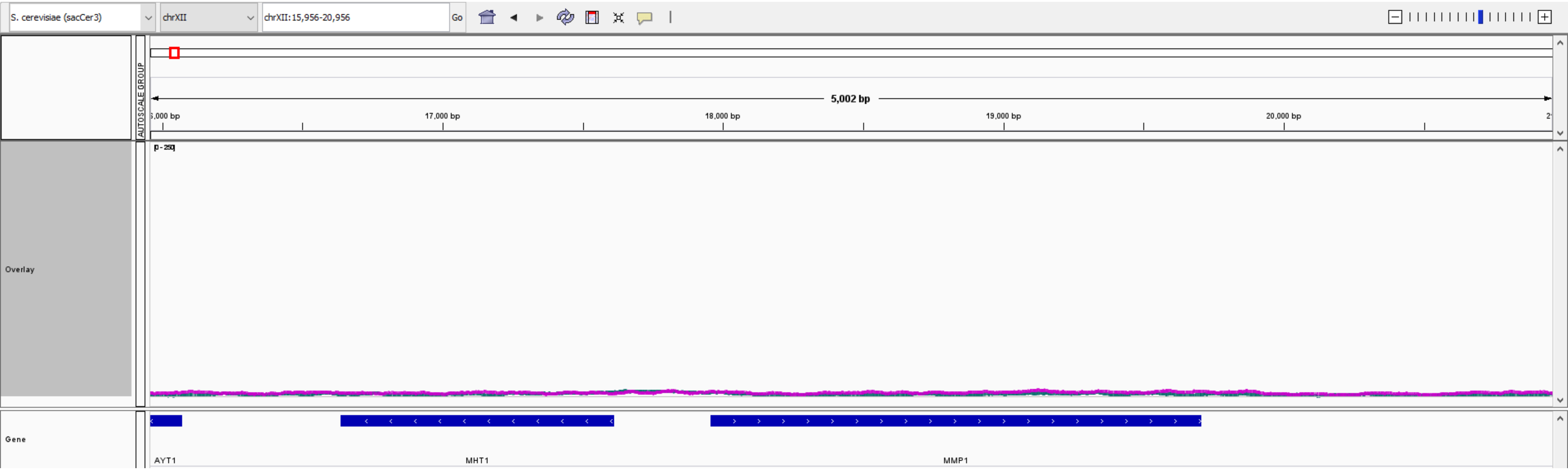

Wild type
snf2Δ

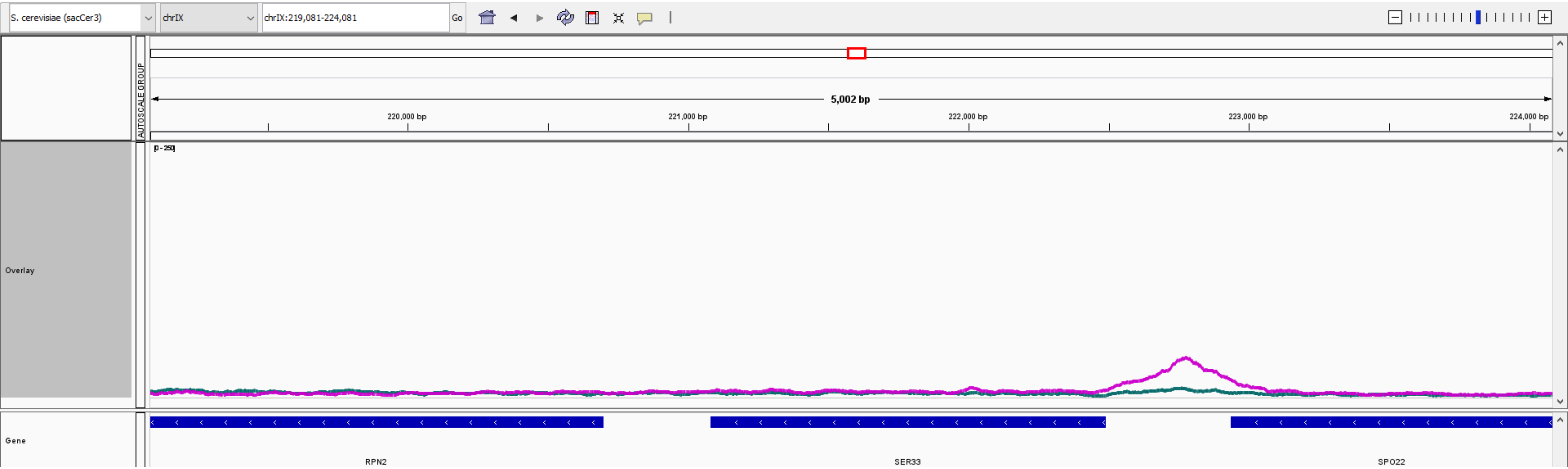

Wild type
snf2Δ

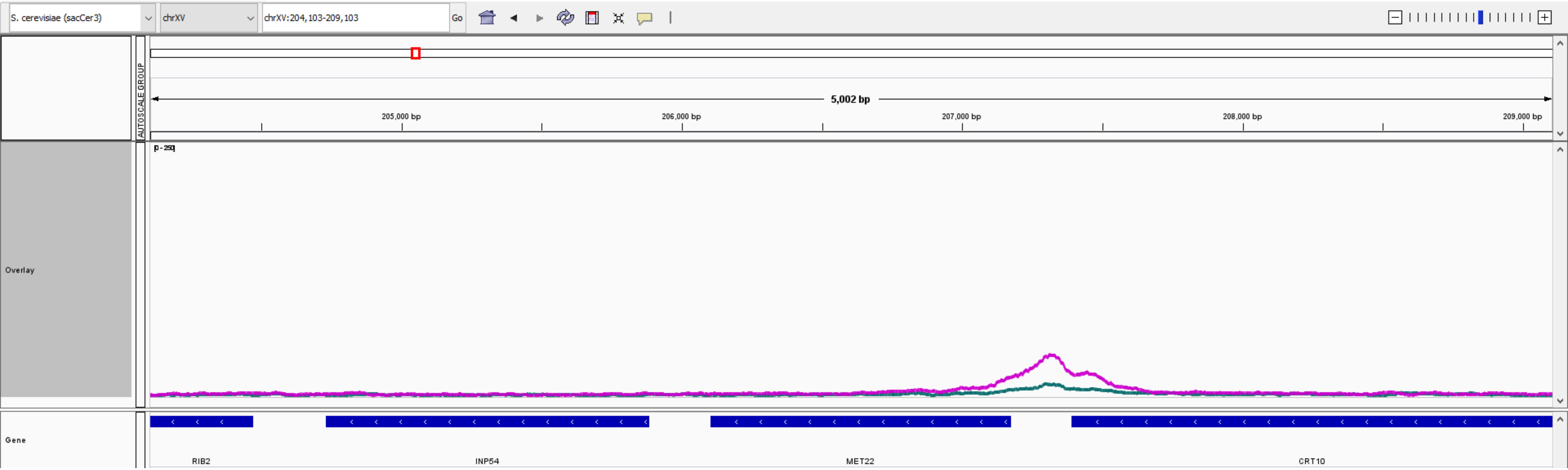

Wild type
snf2Δ

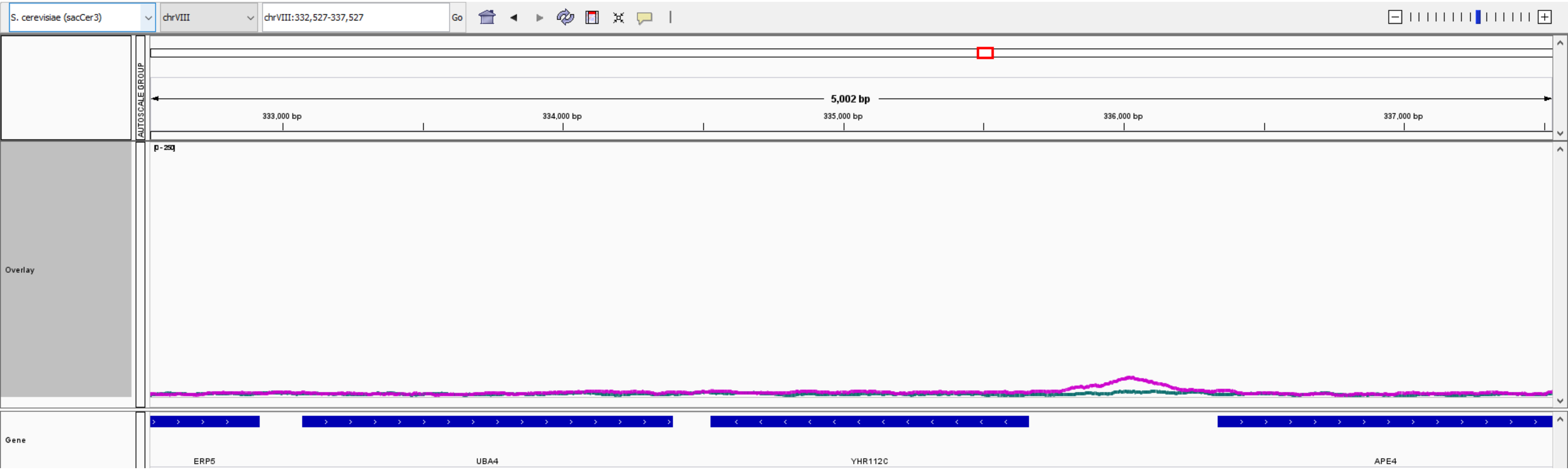

Wild type
snf2Δ

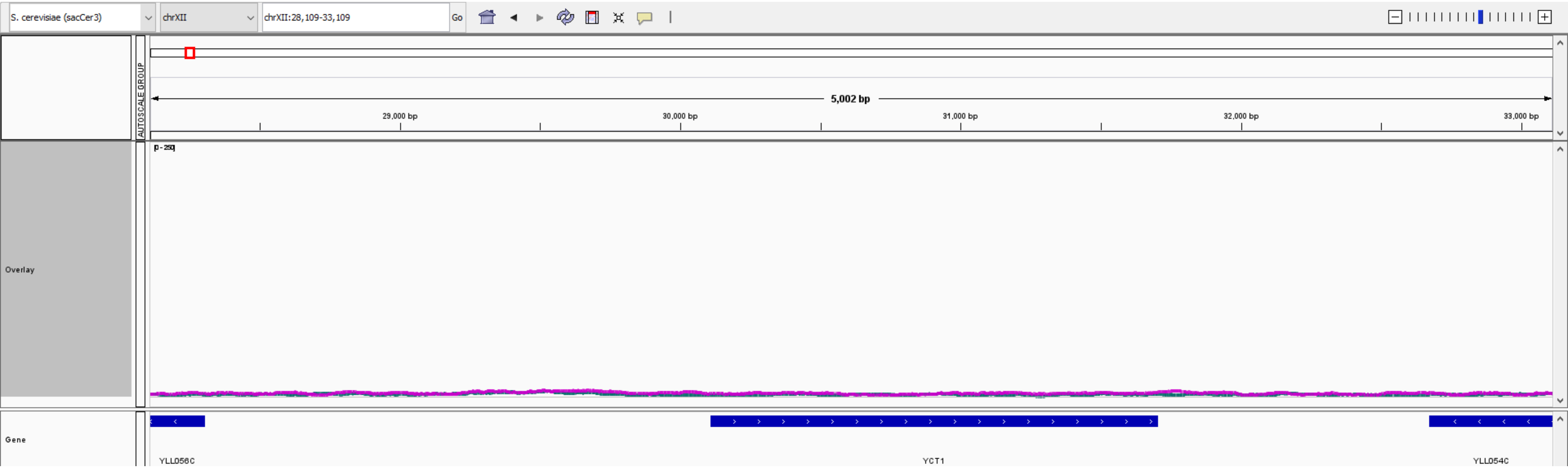

Wild type
snf2Δ

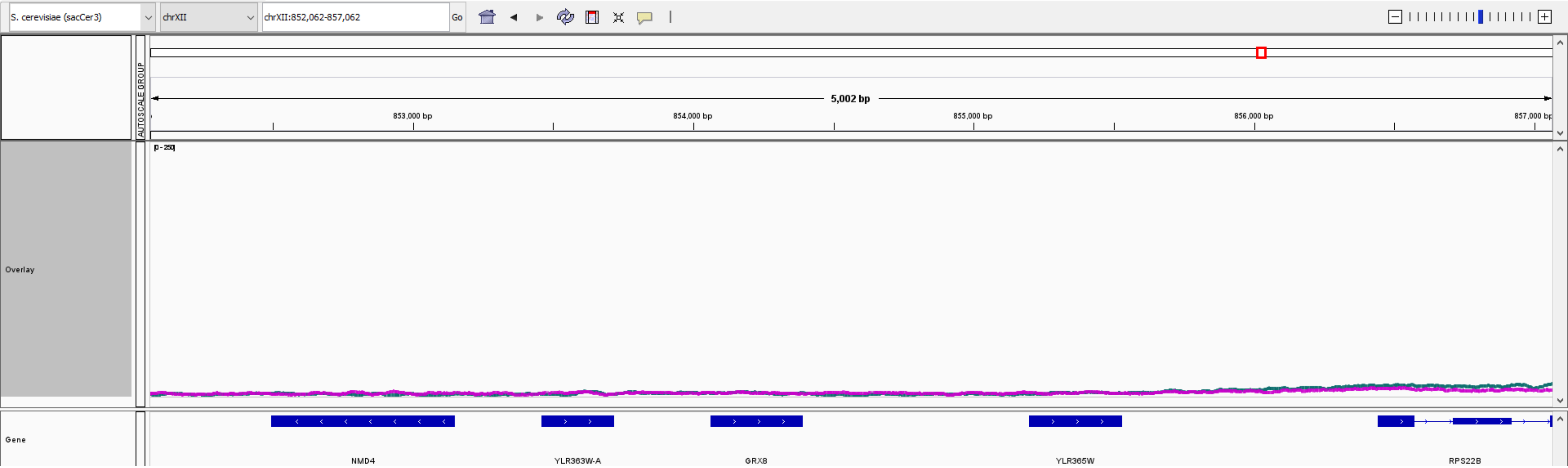

Supplement: gkad711_Supplemental_Files [file gkad711_supplemental_files.zip › Supplementary file S1.pdf]
