# Supplementary material for: The Swi-Snf chromatin remodeling complex mediates gene repression through metabolic control
Source: Nucleic Acids Res. 2023 Aug 31;51(19):10278–91. doi: 10.1093/nar/gkad711 (PMC10602859; doi:10.1093/nar/gkad711)
Supplement: gkad711_Supplemental_Files [file gkad711_supplemental_files.zip › Supplementary Files 1 & 2 Legends.docx]

**Supplementary File 1.** Met4-13Myc ChIP genome track data for wild type and *snf2∆* mutant. Each image represents bigwig data from the IGV viewer (2.8.10) for IP replicate #2 for 13Myc-tagged Met4 in both wild type (green) and *snf2Δ* mutant (magenta) strains over a distance of 5002 bp, with a focus on the TSS region of each *MET* gene. Scales are consistent at 0-250, except at *SUL1* and *HIT1,* where an additional 0-50 scale is included.

**Supplementary File 2.** RNA & ChIP seq datasets used in this study. Tab 1 contains RNA seq data from Figures 1, 2 and associated supplementary figures including log2 cpm values for wild type and *snfΔ* mutants grown in YPD with or without 3 mM exogenous cysteine. This also contains comparisons (log2 fold change – “log2fc) of *snfΔ* mutants vs wild type under each condition. Tab 2 contains RNA seq data used as part of Figure 1 for wild type cells grown in YPD and SD media without amino acids, including log2 cpm and log2 fold change comparisons between conditions. Tab 3 contains ChIP seq Diffbind values for Met4-13Myc tagged wild type (SD and YPD-grown) and YPD-grown *snf2Δ* and *snf5Δ* mutants used in Figures 4 , 5 and associated supplementary figures. Tab 4 contains average cpm values for the *MET* regulon from an RNA seq dataset for wild type vs *snf2Δ* mutants from a Met4 anchor away (Met4-AA) experiment used in Figure 6 and S6. Tab 5 contains Diffbind values for the *MET* regulon from an anti-Rpb1 ChIP seq dataset for wild type vs *snf2Δ* mutants from a Met4 anchor away (Met4-AA) experiment used in Figure S6.
